# New Insights into the Structure–Activity Relationship and Neuroprotective Profile of Benzodiazepinone Derivatives of **Neurounina-1** as Modulators of the Na^+^/Ca^2+^ Exchanger Isoforms

**DOI:** 10.1021/acs.jmedchem.1c01212

**Published:** 2021-11-30

**Authors:** Elisa Magli, Caterina Fattorusso, Marco Persico, Angela Corvino, Gianluca Esposito, Ferdinando Fiorino, Paolo Luciano, Elisa Perissutti, Vincenzo Santagada, Beatrice Severino, Valentina Tedeschi, Anna Pannaccione, Giuseppe Pignataro, Giuseppe Caliendo, Lucio Annunziato, Agnese Secondo, Francesco Frecentese

**Affiliations:** † Department of Pharmacy, University of Naples “Federico II”, Via D. Montesano 49, 80131 Naples, Italy; ‡ Department of Neuroscience, Division of Pharmacology, University of Naples “Federico II”, via Pansini 5, 80131 Naples, Italy; § IRCCS SDN, via E. Gianturco 113, 80143 Naples, Italy

## Abstract

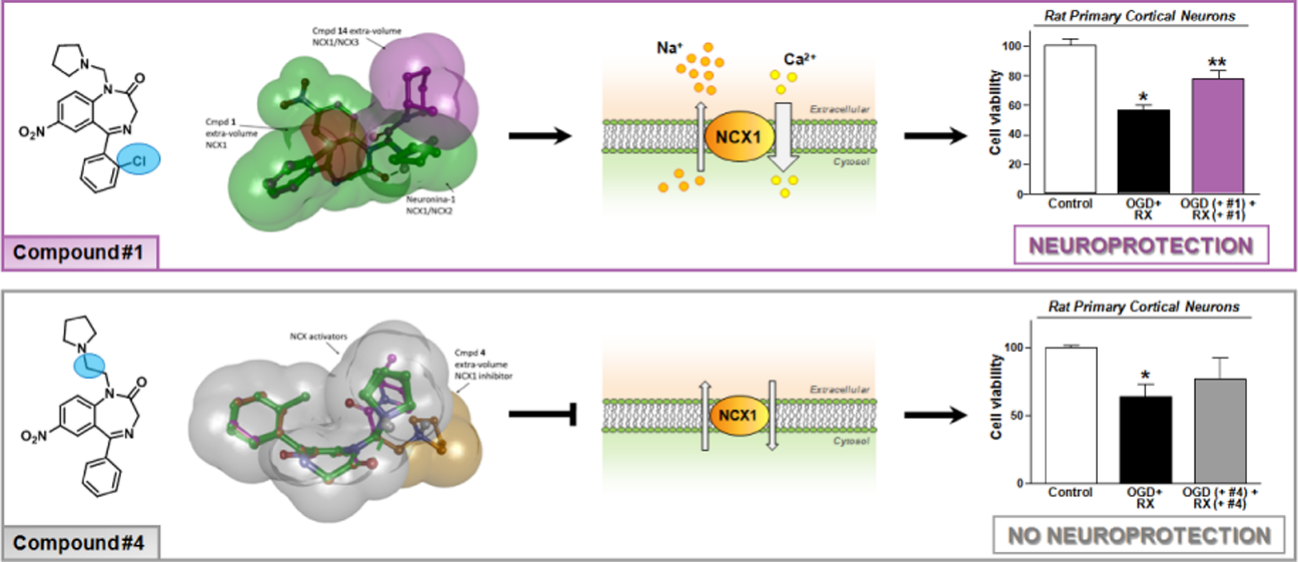

Due to the neuroprotective role of the Na^+^/Ca^2+^ exchanger (NCX) isoforms NCX1 and NCX3, we synthesized novel benzodiazepinone derivatives of the unique NCX activator **Neurounina-1**, named compounds **1–19**. The derivatives are characterized by a benzodiazepinonic nucleus linked to five- or six-membered cyclic amines via a methylene, ethylene, or acetyl spacer. The compounds have been screened on NCX1/NCX3 isoform activities by a high-throughput screening approach, and the most promising were characterized by patch-clamp electrophysiology and Fura-2AM video imaging. We identified two novel modulators of NCX: compound **4**, inhibiting NCX1 reverse mode, and compound **14**, enhancing NCX1 and NCX3 activity. Compound **1** displayed neuroprotection in two preclinical models of brain ischemia. The analysis of the conformational and steric features led to the identification of the molecular volume required for selective NCX1 activation for mixed NCX1/NCX3 activation or for NCX1 inhibition, providing the first prototypal model for the design of optimized isoform modulators.

## Introduction

Calcium (Ca^2+^) ions play an important role in the control of virtually all cellular functions. Maintenance of Ca^2+^ homeostasis is due to the concerted activity of several proteins expressed not only on plasma membrane but also in intracellular organelles storing Ca^2+^.^1^ Among the proteins handling Ca^2+^, the Na^+^/Ca^2+^ exchanger (NCX) has attracted much attention for having, among other electrophysiological features, a high transport capacity. This is of particular importance in those conditions characterized by elevated Ca^2+^ levels such as during the occurrence of neurodegeneration associated to neurological diseases^2,3^ and during myocardial ischemia–reperfusion injury.^4^

Na^+^/Ca^2+^ exchangers belong to a superfamily of Ca^2+^/cation antiporters.^5^ Three isoforms named NCX1, NCX2, and NCX3 have been identified and characterized.^6,7^ These proteins display different localizations: NCX1 is widely expressed in various organs and tissues, whereas NCX2 and NCX3 show a more restricted expression. It is worthy accredited that two of these isoforms, NCX1 and NCX3, are localized intracellularly in specific organelles, where they play an important role in handling the local Ca^2+^ level.^8,9^ Of note, calcium handling outside the mitochondria requires two consecutive steps involving the operation of the Na^+^-sensitive NCLX which may transport either Li^+^ or Na^+^ in exchange for Ca^2+^,^10^ mediating Ca^2+^ transport from the matrix to the intermembrane space, and mNCX3, promoting Ca^2+^ efflux from the intermembrane space to the cytosol.^9^

From the functional point of view, Ca^2+^ and Na^+^ ions are transported by the exchanger through the occupancy of their transport sites. However, they also exert a modulatory role on NCX activity. Accordingly, the exchanger activity is regulated in an allosteric way by the binding of Ca^2+^ and Na^+^ ions to specific NCX domains. For instance, Ca^2+^ binding at the cytosolic face of NCX1 does activate the exchanger, while Na^+^ binding at the cytosolic face exerts inhibitory modulation.^11−14^

In the last 30 years, the occurrence of [Na^+^]_*i*_ and [Ca^2+^]_*i*_ dyshomeostasis has been reported in several neurodegenerative diseases either at neuronal or glial components. Altered expression and activity of NCX isoforms have been also demonstrated in stroke, multiple sclerosis (MS), amyotrophic lateral sclerosis, SMA, Parkinson’s disease, and Alzheimer’s disease.^15−21^ Although the role of each isoform is still under examination, many lines of evidence point toward a neuroprotective effect of NCX activation in most of these neurodegenerative diseases.^21^

Concerning the molecular mechanism underlying the neuroprotection exerted by NCX1 in stroke pathogenesis, functional studies demonstrated its ability to refill the endoplasmic reticulum with Ca^2+^ ions when it works in the reverse mode during hypoxic conditions and ischemic preconditioning (PC).^22,23^

This mechanism is also shared by the NCX3 isoform whose reverse mode may contribute to delay ER stress in hippocampal neurons exposed to Aβ1-42.^20,24^

**Figure 1:**
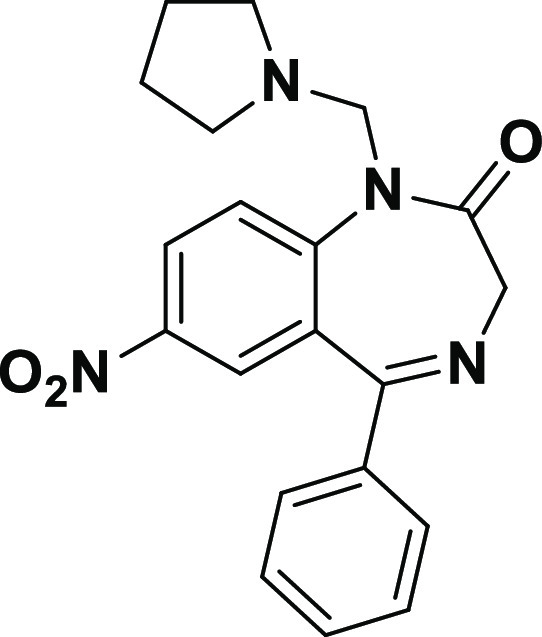
Chemical structure of **Neurounina-1**.

**Neurounina-1** (Figure 1) is an activator of both the reverse and forward mode of NCX1 and NCX2 and it was validated in preclinical models of stroke as a neuroprotective agent.^25^ Then, very recently, its pharmacokinetic profile has been screened in vivo.^26^

In the present study, we synthesized and characterized novel benzodiazepinonic derivatives of **Neurounina-1** (compounds **1–19**; Table 1) with the aim to provide new insights into the structure–activity relationship (SAR) of NCX isoform modulators. All these compounds have been screened with several approaches moving from functional high-throughput screening to patch-clamp electrophysiology and Fura-2AM video-imaging on single-cells. Furthermore, the most selective derivatives have been tested in two different in vitro models of brain ischemia on primary cortical neurons. Computational studies combined with experimental data allowed us to produce the first prototypal model for the design of new molecular entities acting on NCX proteins.

**Table 1:**
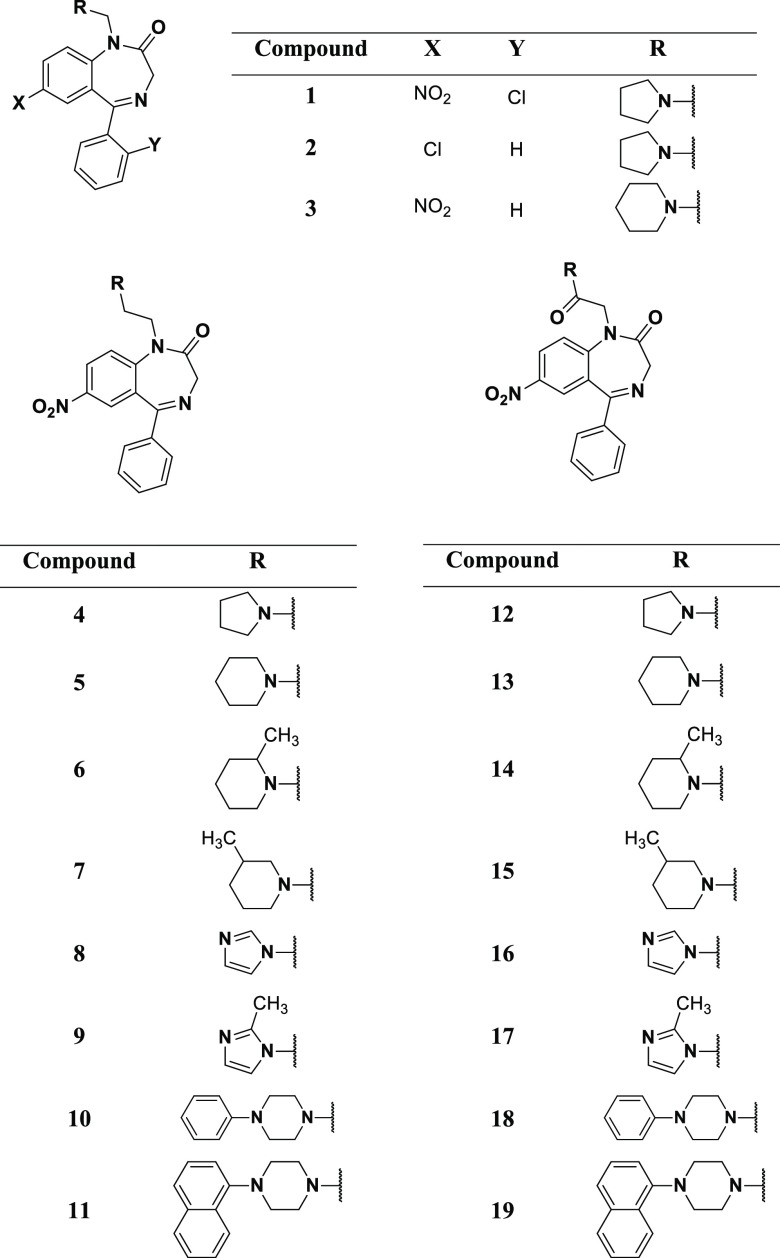
Chemical Structures of Compounds **1–19**

## Results and Discussion

### Design and Synthesis of **Neurounina-1** Derivatives (Compounds **1–19**)

**Scheme 1:**
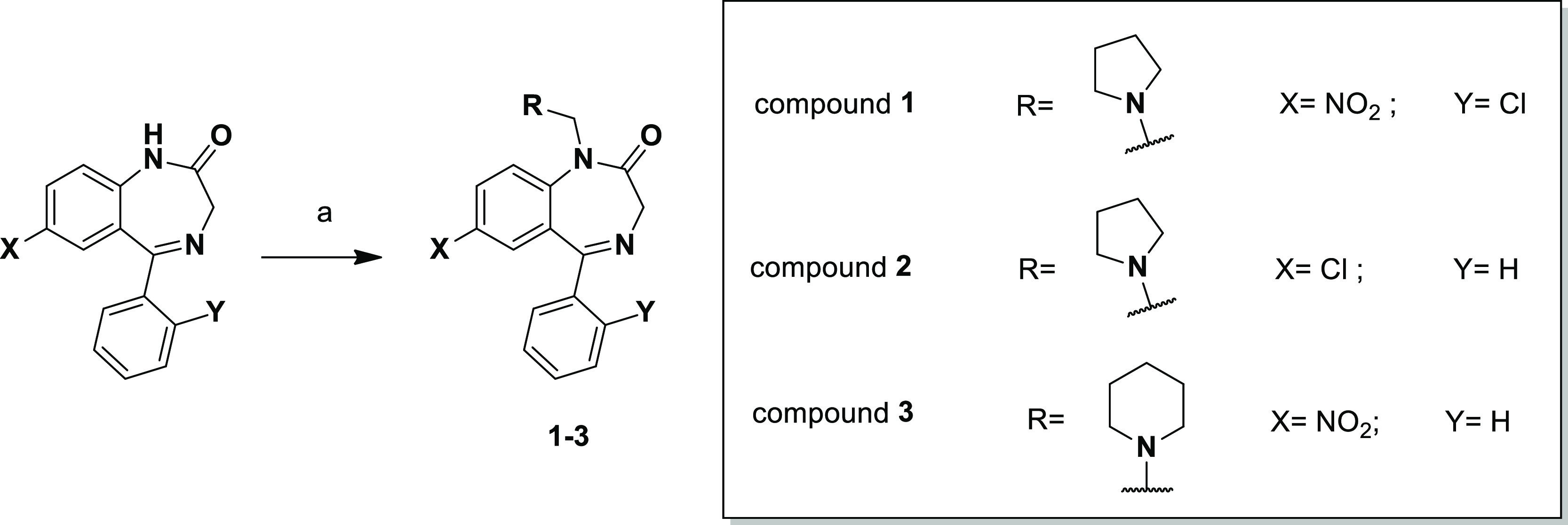
Reagents and Conditions: (a) Formaldehyde Solution 37% wt in H_2_O (10 equiv), Pyrrolidine (Compounds **1** and **2**; 10 equiv) or Piperidine (Compound **3**; 10 equiv), and Glacial Acetic Acid, MW (500 W, 15 min; *T* = 80 °C)

The chemical structures of compounds **1–19** are reported in Table 1. The synthetic strategy for the preparation of compounds **1–3** is reported in Scheme 1; compounds were obtained following, with modifications, the procedures previously reported for the synthesis of **Neurounina-1**.^25^

**Scheme 2:**
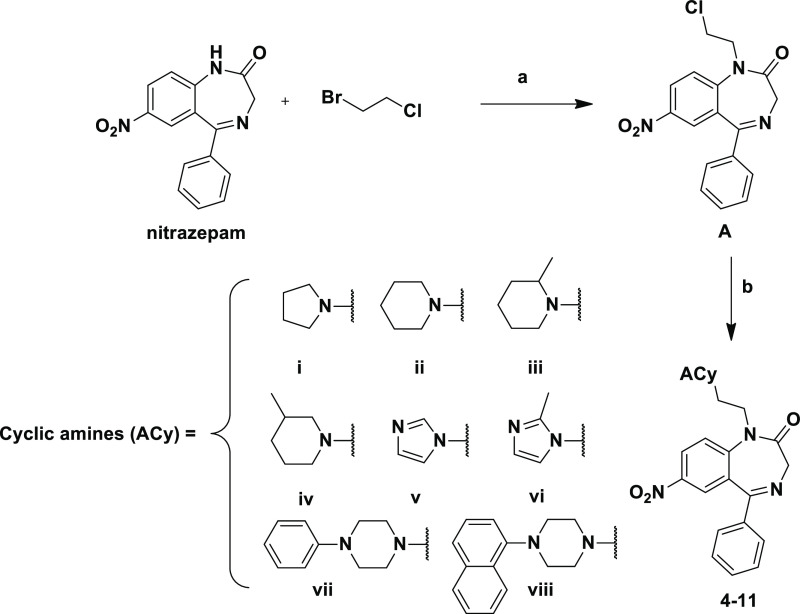
Reagents and Conditions: (a) NaH (60% Dispersion in Mineral Oil), 0 °C, DMF then 4 h, rt; (b) ACy (**i–viii**), K_2_CO_3_ (1.1 equiv), NaI (1.1 equiv), CH_3_CN, Reflux, 3 h

**Scheme 3:**
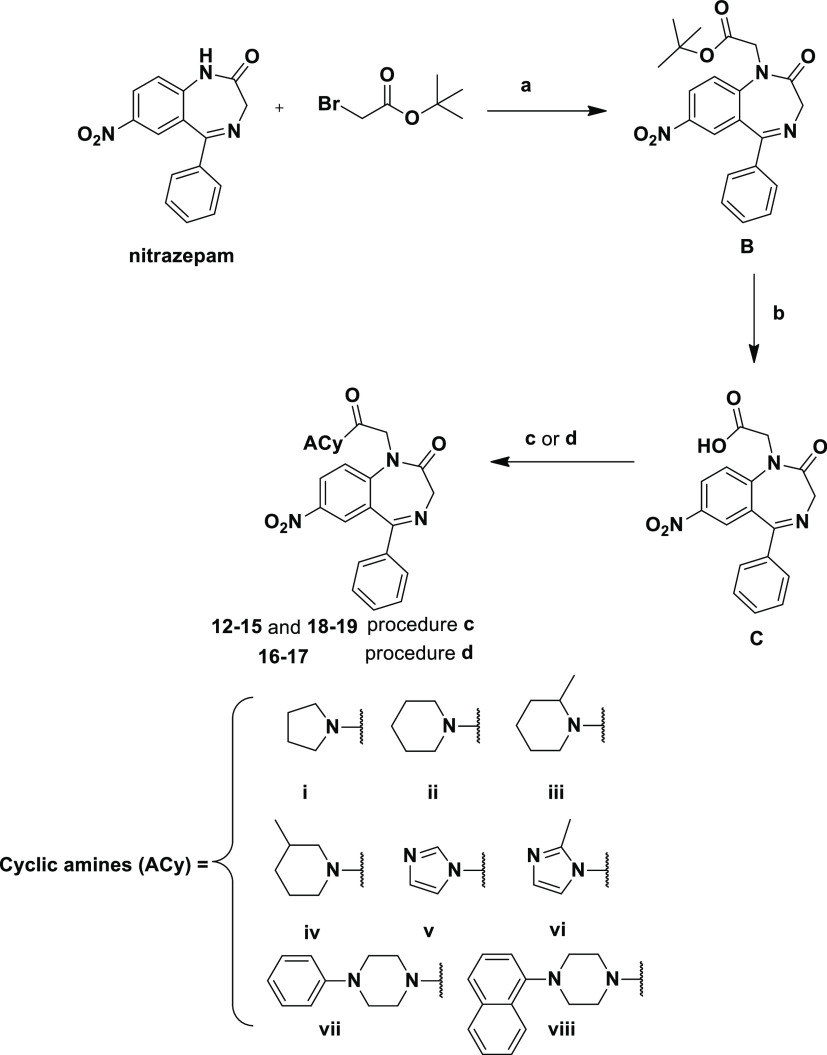
Reagents and Conditions: (a) NaH (60% Dispersion in Mineral Oil), 0 °C, DMF and Then 4 h, rt; (b) Anhydrous CH_2_Cl_2_/TFA (8:2, v/v, 20 mL), rt, 1 h; (c) ACy (**i–iv** and **vii–viii**), TBTU (1.1 equiv), HOBt (1.1 equiv), DIPEA, DMF, Overnight, rt; and (d) (1) SOCl_2_ (10 equiv), Anhydrous CH_2_Cl_2_, Reflux, 1 h and (2) ACy (**v** and **vi**, 2 equiv), Anhydrous Tetrahydrofuran, Overnight, rt

The strategies for the synthesis of the compounds **4–11** and **12–19** are summarized in Schemes 2 and 3, respectively. Compounds **4–11** (Scheme 2) were obtained by the nucleophilic substitution of nitrazepam with 1-bromo-2-chloroethane in the presence of NaH (60% dispersion in mineral oil) in dimethylformamide (DMF) that led to intermediate **A** that was subsequently condensed with the selected cyclic amines (ACy, **i–viii**) in acetonitrile in the presence of K_2_CO_3_ and NaI.

The general procedure for the synthesis of compounds **12–19** (Scheme 3) is as follows: alkylation of nitrazepam with *tert*-butyl bromoacetate in the presence of NaH (60% dispersion in mineral oil) in anhydrous DMF gave intermediate **B**. The reaction with trifluoroacetic acid in anhydrous dichloromethane (20% v/v) allowed the preparation of the carboxylic intermediate **C** that was successively coupled with the opportune cyclic amines (**i–iv** and **vii–viii**) in the presence of TBTU, HOBt, and diisopropylethylamine in DMF to give the desired compounds **12–15** and **18–19**. For the preparation of **16** and **17**, intermediate **C** was converted into the corresponding acyl chloride by reaction with thionyl chloride in anhydrous dichloromethane and was further coupled with imidazole (**v**) and 1-methyl-imidazole (**vi**) in a 1:2 M ratio at room temperature in anhydrous tetrahydrofuran.

### Molecular Characterization of the Newly Synthesized Compounds on NCX1 and NCX3 Activity

**Figure 2:**
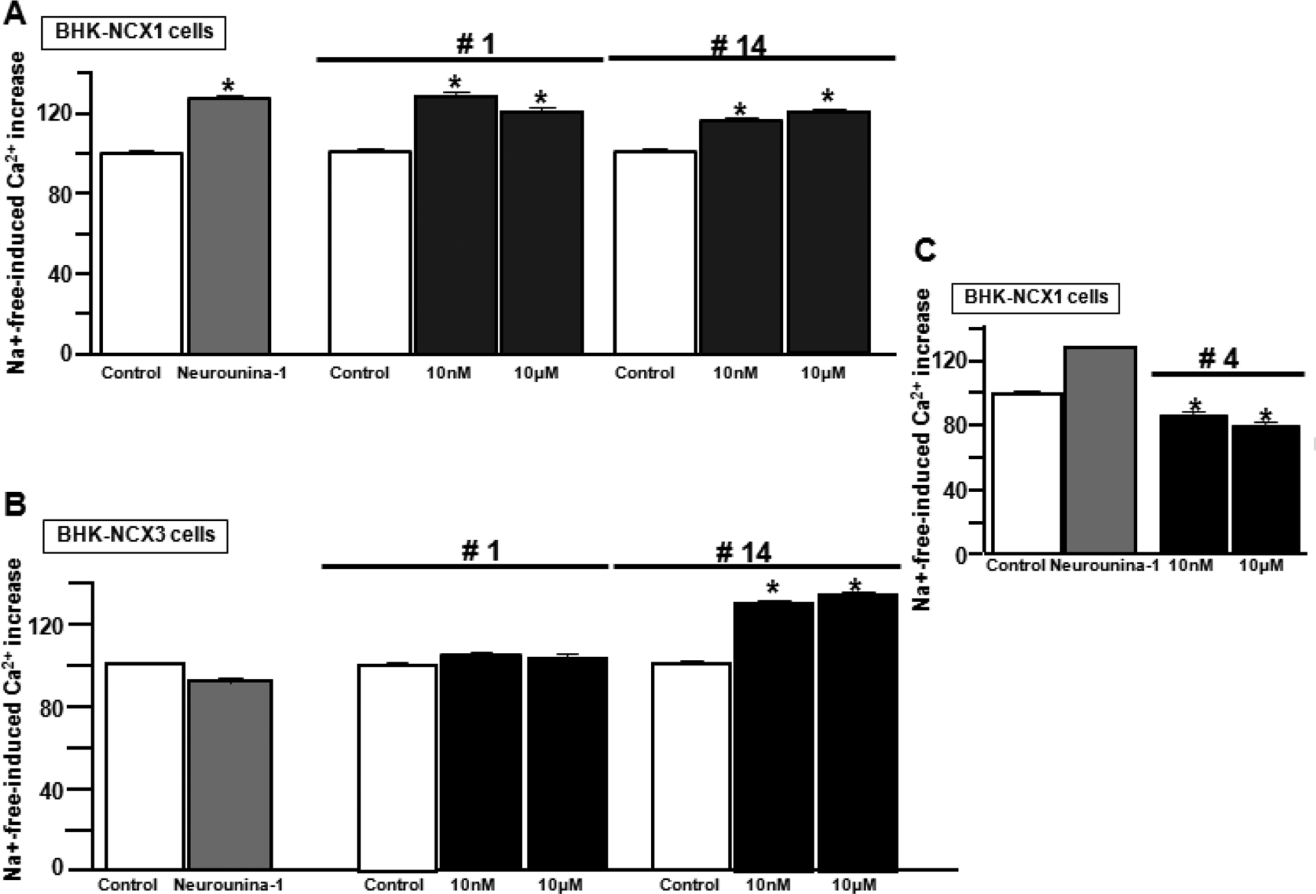
High-throughput screening of the novel benzodiazepinone derivative efficacy on NCX activity in the reverse mode in BHK-NCX1 and BHK-NCX3 cells loaded by Fluo-4. (A) Effect of compound **14** (10 nM and 10 μM), compound **1** (10 nM and 10 μM), and **Neurounina-1** (10 nM) on NCX1 in BHK-NCX1 cells. *p < 0.05 vs control. (B) Effect of the same compounds on NCX3 in BHK-NCX3 cells. *p < 0.05 vs control, **Neurounina-1**, and compound **1**. (C) Effect of two different concentrations of compound **4** on the NCX1 reverse mode in BHK-NCX1 cells. *p < 0.05 vs **Neurounina-1** and internal control (untreated cells). The effects of compounds **1–19** on NCX1 and NCX3 reverse mode activity measured in BHK-NCX1 and BHK-NCX3 loaded by Fura-2 AM have been reported in Figure S1.

In order to identify new compounds able to modulate NCX activity in the reverse mode, BHK-NCX1 and BHK-NCX3 cells were loaded with Fluo-4 (Figure 2) or Fura-2 (Figure S1) and then superfused in the high-throughput screening setting with a single pulse of Na^+^-deficient NMDG^+^ medium (Na^+^-free) in the presence of 10 nM and 10 μM concentrations of each compound in the case of Fluo-4 experiments or in the presence of 10 μM of each compound in the case of Fura2-loaded cells. Among all the 19 compounds screened, we identified a selective NCX1 inhibitor, compound **4**, and two activators, compounds **1** and **14**. In particular, compound **14** was able to stimulate NCX1 and NCX3, whereas compound **1** resulted in a selective NCX1 activator, confirming previous results obtained by single-cell Fura-2-monitored Ca^2+^ influx techniques.^27^

Interestingly, compound **1** activity has been recently linked to learning and memory function through the NCX1-dependent CaMKIIα phosphorylation.^24^ Furthermore, the involvement of NCX3 isoform in hippocampal long-term potentiation and spatial learning and memory has also been demonstrated.^28^ Therefore, the newly synthesized compound **14** was selected to compare its putative neuroprotective profile with those of compound **1** under anoxic/hypoxic conditions. In this respect, we adopted two different anoxic/hypoxic models consisting on the exposure of primary cortical neurons to oxygen and glucose deprivation (OGD), followed by reoxygenation (RX) or chemical hypoxia followed by RX (see the next paragraph).

**Figure 3:**
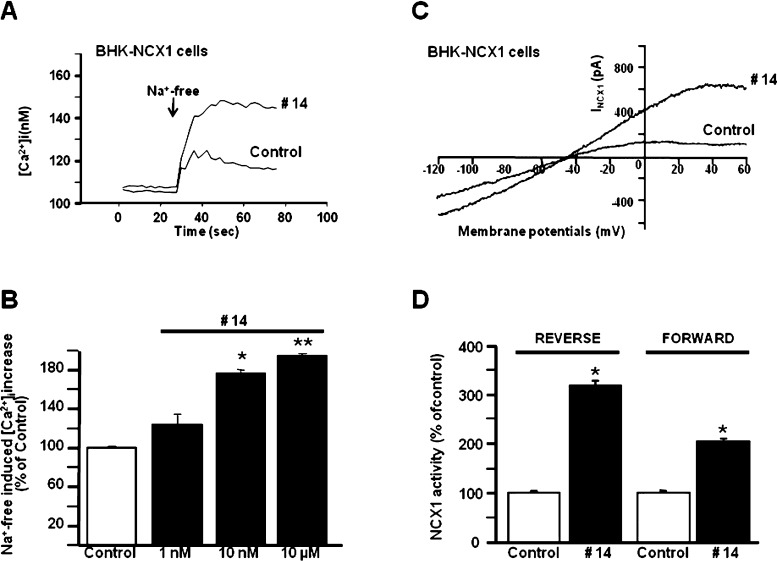
Effect of compound **14** on NCX1 activity in BHK-NCX1 cells. (A,B) Representative superimposed traces and quantification of the effect of compound **14** on Na^+^-free-induced [Ca^2+^]_*i*_ increase through NCX1 reverse mode of operation in Fura-2-loaded BHK-NCX1 cells. The bar graph reports the mean ± SEM of the maximal [Ca^2+^]_*i*_ responses measured in approximately 60 cells per group. Averaged data from four different experimental sessions were normalized as the percentage of controls. *p < 0.05 vs control and 1 nM; **p < 0.05 vs all. (C,D) Representative I_NCX_ traces recorded by whole-cell patch-clamp electrophysiology in control cells and in cells treated with compound **14** (10 nM). The bar graphs in (D) report the mean ± SEM of the forward and reverse NCX1 current densities, respectively, measured in at least 10 cells for each experimental group. Reverse I_NCX_ amplitude was measured at +60 mV, while forward I_NCX_ was measured at −120 mV. *p < 0.05 vs each internal control.

**Figure 4:**
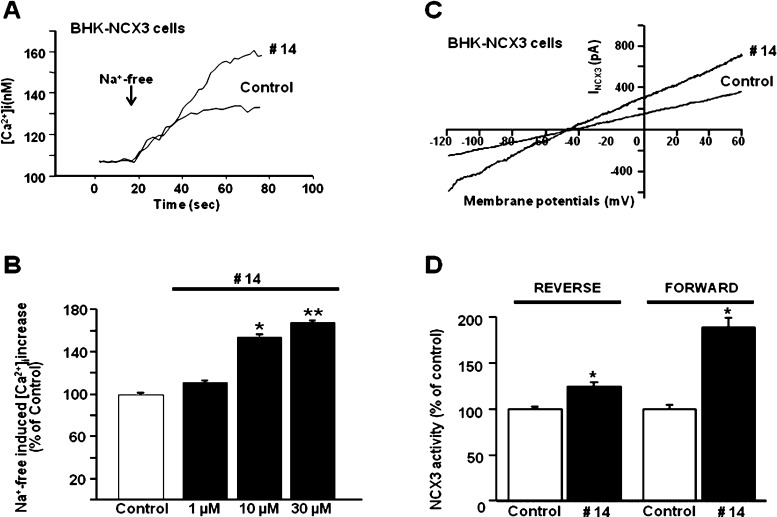
Effect of compound **14** on NCX3 activity in BHK-NCX3 cells. (A,B) Representative superimposed traces and quantification of the effect of compound **14** on Na^+^-free-induced [Ca^2+^]_*i*_ increase through NCX3 reverse mode of operation in Fura-2-loaded BHK-NCX3 cells. The bar graph reports the mean ± SEM of the maximal [Ca^2+^]_*i*_ responses measured in approximately 50 cells per group. Averaged data from four different experimental sessions were normalized as the percentage of controls. *p < 0.05 vs control and 1 μM; **p < 0.05 vs all. (C,D) Representative I_NCX_ traces recorded by whole-cell patch-clamp electrophysiology in control cells and in cells treated with compound **14** (10 μM). The bar graphs in (D) report the mean ± SEM of the forward and reverse NCX3 current densities, respectively, measured in nine cells for each experimental group. Reverse I_NCX_ amplitude was measured at +60 mV, while forward I_NCX_ was measured at −120 mV. *p < 0.05 vs each internal control.

Furthermore, NCX activity in the reverse mode of operation was also studied on single cell by Fura-2AM video-imaging in BHK-NCX1 and BHK-NCX3 cells (Figures 3 and 4). In these stably transfected cells, Na^+^-free perfusion caused a rapid rise in [Ca^2+^]_*i*_, which was significantly enhanced, in a dose-dependent way, by compound **14** (EC_50_ = 3.5 nM for NCX1 and EC_50_ = 2 μM for NCX3; Figures 3 and 4). Furthermore, patch-clamp electrophysiology in the whole-cell configuration was used to measure NCX activity both in the forward and reverse mode of operation.

The whole-cell current was measured at +60 mV (reverse mode) and −120 mV (forward mode) using a ramp-clamp protocol for NCX currents (see Methods). To isolate NCX currents (I_NCX_), cells were recorded for 5 min with the well-known NCX inhibitor NiCl_2_ (5 mM). The Ni^2+^-sensitive component, representing the isolated I_NCX_, was obtained as previously reported.^15,29^ No current corresponding to I_NCX_ was recorded in BHK-Wt cells.^29^ Besides its highest potency in modulating NCX1, compound **14** displayed a greater selectivity for the NCX3 forward mode.

Accordingly, while compound **14** strongly enhanced both the outward (reverse mode) and inward (forward mode) direction of I_NCX1_ and I_NCX3_ (Figures 3 and 4), it showed a much higher effect (i) on the I_NCX1_ reverse mode than on its forward mode and (ii) on the I_NCX3_ forward mode than on its reverse mode.

**Figure 5:**
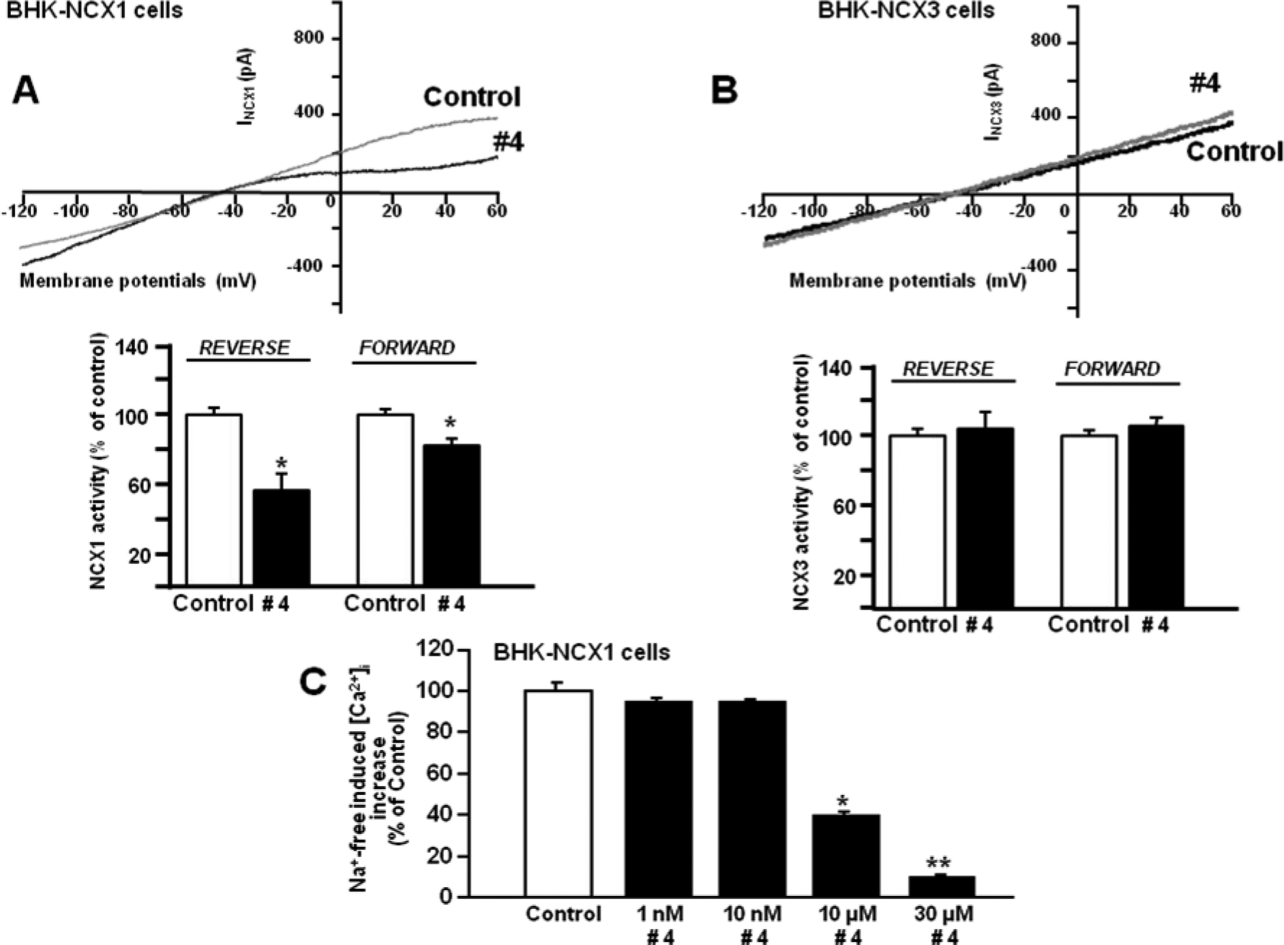
Effect of compound **4** on NCX1 and NCX3 activity in BHK-NCX1 and BHK-NCX3 cells. (A) Representative I_NCX_ traces recorded in BHK-NCX1 by whole-cell patch-clamp electrophysiology in control cells and in cells treated with compound **4** (10 μM). The bar graphs in (A) report the mean ± SEM of the reverse and forward NCX1 current densities, respectively, measured in 10 cells for each experimental group. Reverse I_NCX_ amplitude was measured at +60 mV, while forward I_NCX_ was measured at −120 mV. *p < 0.05 vs each internal control. (B) Representative I_NCX_ traces recorded in BHK-NCX3 by whole-cell patch-clamp electrophysiology in control cells and in cells treated with compound **4** (10 μM). The bar graphs in (B) report the mean ± SEM of the reverse and forward NCX1 current densities, respectively, measured in 10 cells for each experimental group. Reverse I_NCX_ amplitude was measured at +60 mV, while forward I_NCX_ was measured at −120 mV. (C) Quantification of the concentration-dependent effect of compound **4** on Na^+^-free-induced [Ca^2+^]_*i*_ increase through NCX1 reverse mode of operation in Fura-2-loaded BHK-NCX1 cells. The bar graph reports the mean ± SEM of the maximal [Ca^2+^]_*i*_ responses measured in approximately 30 cells per group. Averaged data from four different experimental sessions were normalized as the percentage of controls. *p < 0.05 vs control, 1, and 1 0 nM; **p < 0.05 vs all.

Moreover, compound **4** displayed specific inhibitory action on NCX1, thus representing a new selective inhibitor of the exchanger isoform 1 (Figure 5C). Accordingly, in BHK-NCX1 cells, compound **4** reduced, in a concentration-dependent manner (IC_50_ = 10 μM), Na^+^-free induced [Ca^2+^]_*i*_ increase mediated by the exchanger isoform reverse mode. Interestingly, compound **4** was more efficacious on the reverse mode of NCX1 than on its forward mode as measured by patch-clamp electrophysiology in the whole-cell configuration (Figure 5A,C). In contrast, it failed to modulate the NCX activity in BHK-NCX3 cells (Figures 5B and S1).

### Effect of the Benzodiazepinone Derivatives, Compounds **1** and **14**, Enhancing NCX Activity, and Compound **4**, Inhibiting NCX Activity, on Cell Survival of Primary Cortical Neurons Exposed to Hypoxic Conditions Mimicking Stroke

Once identified as NCX activity modulators, compounds **1**, **4**, and **14** were tested on cell viability of primary rat cortical neurons exposed to OGD followed by RX, an experimental condition mimicking hypoxic conditions occurring during stroke. These compounds were added during either OGD or RX phases at the corresponding median effective concentration for their NCX stimulating or inhibiting activity.

**Figure 6:**
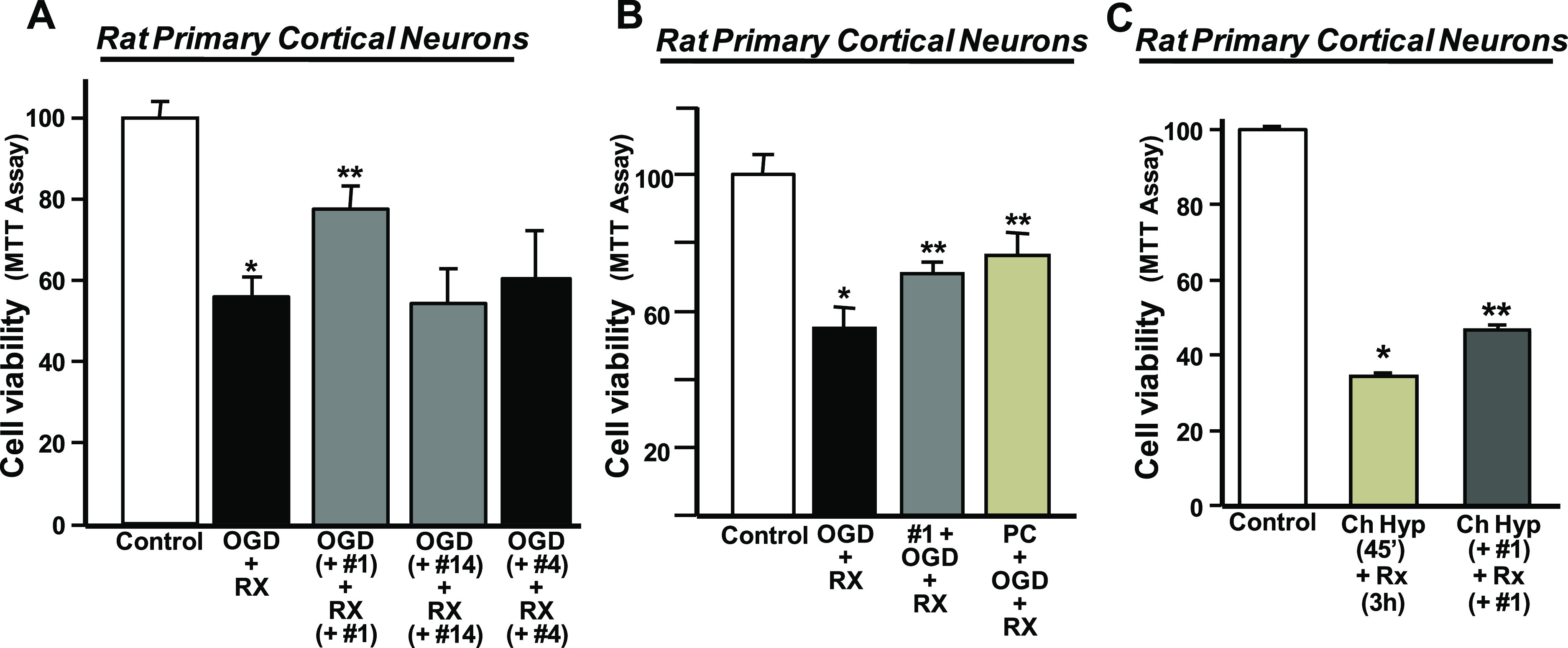
Neuroprotective effect of compound **1** in primary cortical neurons exposed to OGD followed by RX or chemical hypoxia followed by RX. (A) Bar graph depicting the effect on cell viability of compounds **1** (10 nM), **14** (10 μM), and **4** (10 μM) added during both OGD and RX. Cell viability was measured at the end of RX. Data are means ± SEM of five separate experiments and are reported as the percentage of cell viability in the control (100% viability). *p < 0.05 vs normoxic cells (control); **p < 0.05 vs OGD/Rx. (B) Bar graph depicting the effect on cell viability of compound **1** preincubated before OGD + RX. After preincubation, compound **1** was removed during OGD + RX and it was compared to ischemic PC. *p < 0.05 vs normoxic cells (control); **p < 0.05 vs OGD/Rx. (C) Bar graph depicting the effect on cell viability of compound **1** (10 nM) added during both chemical hypoxia (Ch Hyp, 45′) and RX (3 h). Cell viability was measured at the end of RX. Data are means ± SEM of three separate experiments and are reported as the percentage of cell viability in the control (100% viability). *p < 0.05 vs normoxic cells (control); **p < 0.05 vs Ch Hyp + Rx.

Under these anoxic conditions, only compound **1** showed a neuroprotective profile in cortical neurons (Figure 6A) in a concentration-dependent way (Figure S2). Furthermore, when compound **1** was preincubated in rat primary cortical neurons and then removed during the exposure to OGD and RX phases, it continued to protect neurons from hypoxic conditions (Figure 6B). Considering that this represents a more demanding experimental paradigm, compound **1** may be considered an interesting tool with a neuroprotective profile. In fact, its neuroprotective effect in cortical neurons exposed to hypoxic conditions was similar to that produced by ischemic PC,^30,31^ a short stimulus inducing ischemic tolerance in neurons (Figure 6B). Furthermore, to better investigate the neuroprotective profile of compound 1, it was tested in primary cortical neurons exposed to chemical hypoxia, followed by RX, a detrimental condition undermining the mitochondrial function. Under these conditions, compound **1** significantly reduced cell suffering (Figure 6C) in a concentration-dependent way (Figure S2), thus reinforcing the concept of its neuroprotective profile under anoxic/hypoxic conditions. Interestingly, NCX1 and NCX3 are upregulated in those brain regions protected by PC treatment.^30,31^ However, the specific role of each mode of operation in the neuroprotective effect remains obscure and very difficult to define in this experimental paradigm. In consideration of our data showing the unique neuroprotective effect of compound **1** and the lack of neuroprotection by compound **14**, we could speculate that in general, the ability to stimulate the NCX reverse mode could have a major readout than that of forward mode stimulation. In this view, the much greater effect of compound **14** on the forward mode of NCX3 than on its reverse mode could also play a role. Indeed, previous studies showed a neuroprotective effect of ER Ca^2+^ refilling mediated by NCX both during persistent anoxic conditions and ischemic PC.^22,23^ Therefore, the excessive activation of the NCX3 forward mode determining Ca^2+^ extrusion may negatively counterbalance the activation of its reverse mode, favoring Ca^2+^ entrance.

### Computer-Aided SAR Analysis

**Figure 7:**
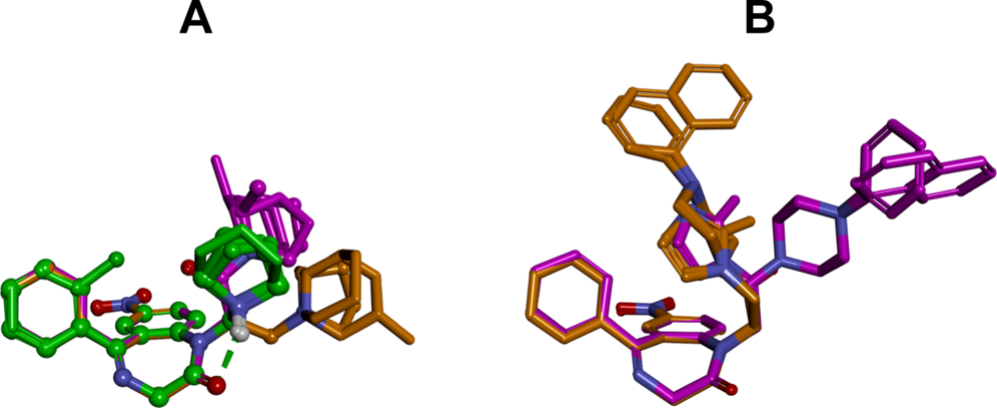
Lowest energy conformers of **Neurounina-1** and compounds **1–19** superimposed by the heavy atoms of the benzodiazepinone ring system. (A) Compounds with aliphatic R groups: **Neurounina-1**, compounds **1–3**, **4–7**, and **12–15**. (B) Compounds with an aromatic R group: **8–11** and **16–19**. Carbon atoms are colored according to the spacer group: **Neurounina-1**, compounds **1–3** (carbon atoms: green), **4–11** (carbon atoms: orange), and **12–19** (carbon atoms: magenta). The active analogues (**Neurounina-1**, compounds **1**, **4**, and **14**) are displayed as ball and sticks; the inactive analogues are displayed as sticks. Heteroatoms are colored by atom type; hydrogen atoms are omitted for clarity of presentation except for those involved in hydrogen bonds. Hydrogen bonds are displayed as green dashed lines.

In order to analyze SARs of **Neurounina-1** and compounds **1–19**, computational studies have been performed. Molecular simulations were carried out considering the compounds in their calculated prevalent ionic format blood (7.4) and cytoplasmic (7.2) pH values (Table S1); accordingly, **Neurounina-1** and compounds **1–7** were considered protonated, while compounds **8–19** were considered neutral. The conformational space of the compounds has been sampled by using a stochastic conformational search algorithm, while the generalized Born model has been applied to mimic an aqueous environment. Resulting conformers were ranked by their conformational energy, analyzed, and classified according to their conformational features (see the Experimental Section for details). The spatial orientation of the R groups with respect to the benzodiazepinone ring was mapped, and the conformers were accordingly classified into families (Tables S2–S21). This allowed the identification of the most favored conformational family of each compound (Table 2 and Figure 7) and the generation of a ligand-based pharmacophore model according to the available experimental data (Figure 7; see the Experimental Section for details).

**Table 2:**
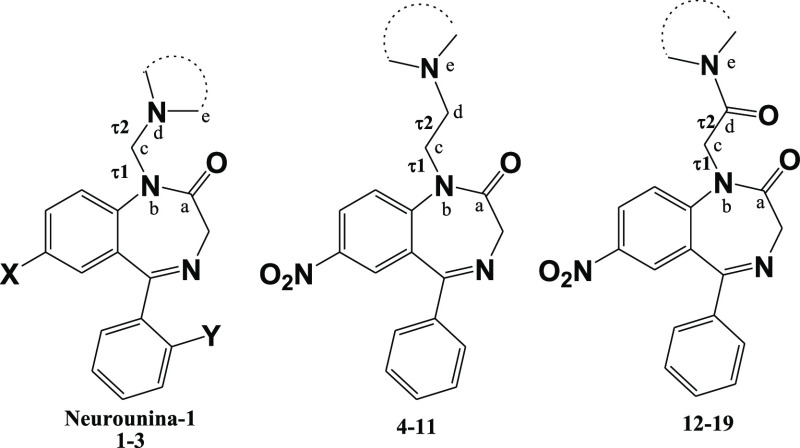
Lowest Energy Conformational Families of **Neurounina-1** and Compounds **1–19**^a^

				torsional angles^c^^,^^d^ (deg)	
compound	family	Δ*E*_GM_^b^ (kcal/mol)	occurrence rate (%)	τ_1_	τ_2_
**Neurounina-1**	Ic	0.00	11	–57	175
**1**	Ic	0.00–1.34	22	–55	177
**2**	Ic	0.00	14	–58	179
**3**	Ic	0.00	20	–64	173
**4**	IIc	0.00–0.92	33	74	–179
**5**	IIc	0.00–0.82	26	74	–175
**6**	IIc	0.00–3.0 2	15	74	–171
**7**	IIc	0.00–3.00	22	74	–175
**8**	Ib	0.00–0.11	32	–67	–59
**9**	Ib	0.00–0.01	36	–67	–55
**10**	Ib	0.00–4.77	42	–80	–61
**11**	Ib	0.00–4.56	57	–75	–63
**12**	IIIc	0.00–0.31	50	–133	–165
**13**	IIIc	0.00–0.35	57	–130	–168
**14**	IIIc	0.00–0.63	42	–130	–169
**15**	IIIc	0.00–0.56	31	–128	–166
**16**	IIIa	0.00–0.97	37	–143	59
**17**	IIIa	0.00	37	–143	60
**18**	IIIc	0.00–3.51	56	–127	–172
**19**	IIIc	0.00–4.97	59	–111	–145

a Torsional angle, Δ*E*_GM_, and occurrence rate values are reported.

b The values reported refer to the lowest and the highest energy conformers of the family, the presence of just one value means that all conformers converged to the same structure.

c The conformational/configurational enantiomer with the opposite flip present the same absolute values of all torsional angles with the opposite sign.

d The values reported refer to the lowest energy conformers of each family.

All compounds were characterized by the presence of two possible flips of the benzodiazepinone system, which produced two sets of isoenergetic conformers (named A and B) with nonsuperimposable, specular structures (conformational enantiomers; Figure S3); in the case of the chiral derivatives **6**, **7**, **14**, and **15**, tested as racemic mixtures, the specular conformers corresponded to those of the two configurational enantiomers. The torsion angle values in Table 2 are referred to the A set of conformers (τ_flip_ = −65°; S-chirality), and the B set of conformers (τ_flip_ = 65°; R-chirality) presented the same energy values and the torsion angle values with the opposite sign. Currently, we have no information to speculate about the stereospecificity of the compounds.

**Figure 8:**
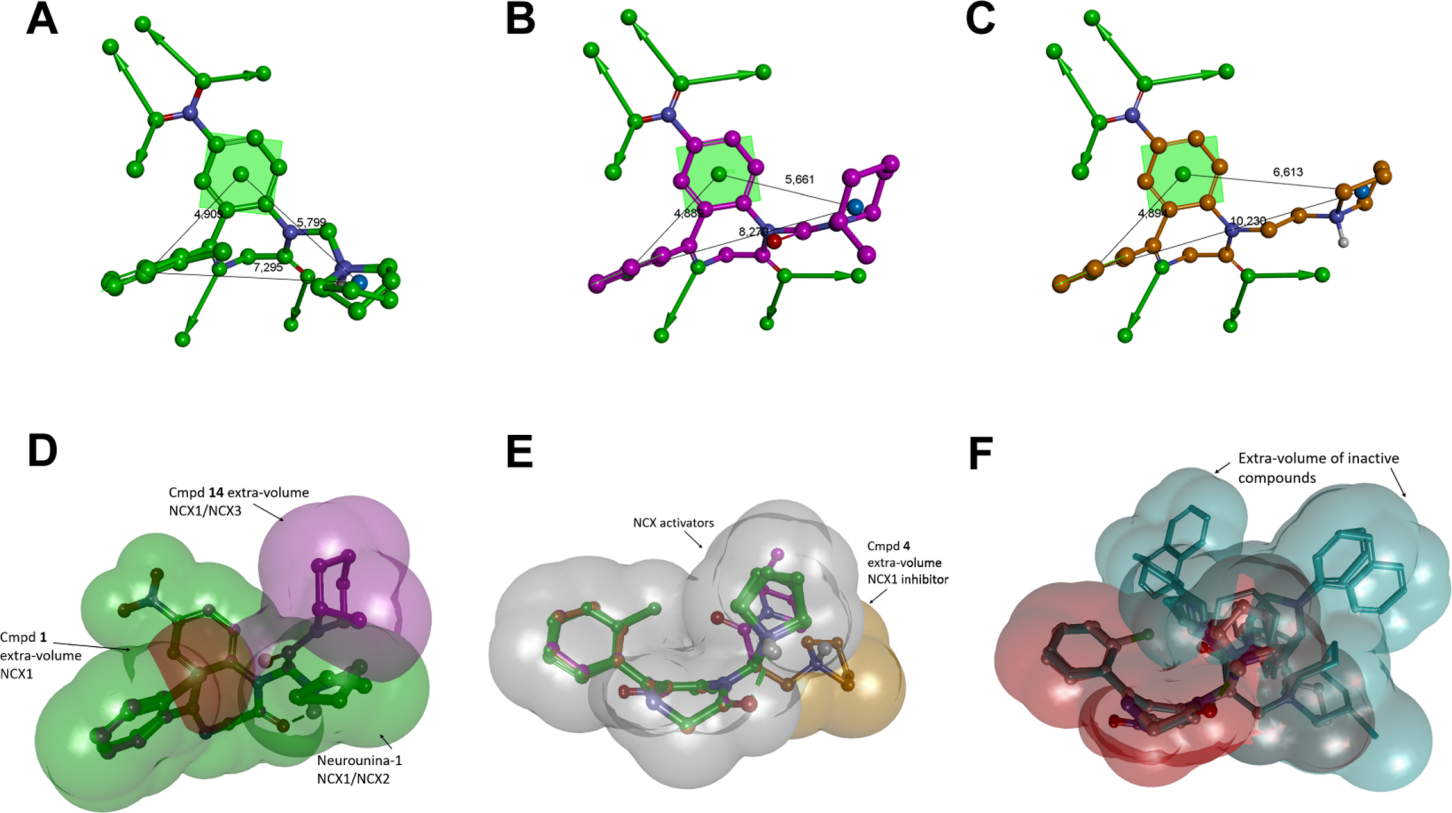
(A) **Neurounina-1** and compound **1** (NCX1 activators); (B) compound **14** (NCX1/3 activator); and (C) compound **4** NCX1 (inhibitor); pharmacophore features: aromatic ring (centroid and plane; green), hydrophobic-aliphatic group (centroid; blue), hydrogen bond acceptor head/tail (balls and arrows; green), distances (Å; black). (D–F): shape features. All structures are superimposed by the carbon atoms of the benzodiazepinone moiety and displayed as ball and sticks. Heteroatoms are colored by atom type: carbon atoms are colored: **Neurounina-1** and compound **1**, green; compound **4**, orange; and compound **14**, magenta. Hydrogen atoms are omitted for the clarity of presentation.

Some interesting considerations can be done on the base of the obtained results. **Neurounina-1** and compounds **1–3**, which present a methylene group as the spacer and a pyrrolidine or piperidine ring as R, showed the same conformational preference, characterized by the *g*^–^and anticonformation of τ_1_ and τ_2_, respectively (Table 2). The formation of a six-membered intramolecular hydrogen bond between the protonated nitrogen of R and the carbonyl oxygen of the benzodiazepinone ring represented the driving force for the resulting conformational behavior (Figure 7A). Accordingly, the selectivity of compound **1** toward NCX1^27^ compared to **Neurounina-1**, which stimulates both NCX1 and NCX2,^25^ is not due to conformational effects but to the steric hindrance introduced by the chlorine atom at the ortho position of the pendant phenyl ring, which is not tolerated by the NCX2 binding site (Figure 8D).

Importantly, the complete loss of activity of **3**, presenting a piperidine ring in place of the pyrrolidine substituent of **Neurounina-1**, revealed the very strict shape features required by the NCX cleft accommodating the R group (Figure 8F). Finally, the inactivity of **2** indicated that the nitro substituent at the benzo-fused ring of **Neurounina-1** is involved in key interactions with the protein binding site, which cannot be any more established when it is replaced by a chlorine atom. This suggested the involvement of the hydrogen bond acceptor features of the nitro group at the benzo-fused ring (Figure 8A–C).

Interestingly, when the spacer of **Neurounina-1** is extended to an ethylene group, the resulting compound **4** was still able to bind to NCX1, but the effect changed from activation to inhibition. In this case, the lowest energy conformational family is characterized by the *g*^+^ conformation of τ_1_ and the anticonformation of τ_2_, respectively (colored in orange in Figures 7A and 8C). Consequently, as evidenced in Figure 8E, the pyrrolidine ring of the NCX inhibitor **4** is differently oriented with respect to those of the activators **Neurounina-1** and compound **1**.

This suggested that **4** binds to (stabilizes) a different NCX1 conformation with respect to that bound by activators, in which the protein binding partner of the pyrrolidine ring is the same, but differently oriented. In support of this hypothesis, similarly to what was observed for **3** with respect to **Neurounina-1**, regardless of preserving the same conformational preference (Table 2), the enlargement of the pyrrolidine ring of the NCX1 inhibitor **4** to a piperidine ring (**5**) as well as to methyl-substituted piperidine rings (**6** and **7**) resulted in inactive compounds, thus confirming the very strict shape requirements for the R group observed for activators.

On the other hand, the replacement of the pyrrolidine ring of **4** with an imidazole ring (**8**) also led to a complete loss of activity. This structural modification changed both the electronic nature of R and the conformational preference of the resulting compound (**8**; Table 2; Figure 7B). The same conformational preference is shared by **9–11** (colored in orange in Figure 7B), where the ethylene spacer is combined with other aromatic R groups, which also resulted to be inactive. As evidenced in Figures 7 and 8F, when compared to those of the active compounds, the R groups of **8–11** occupy different regions of the space with respect to the benzodiazepinone system.

A third type of spacer was tested by replacing a methylene unit with a carbonyl group (**12–19**) and, by consequence, changing the amine function of the R groups into an amide function, not protonated at physiological pH.

Due to the sp^2^ hybridization of the introduced carbonyl group and the attraction between the carbonyl oxygen and the hydrogen atoms of the pendant phenyl ring, the introduction of such a spacer strongly affected the value of τ_1_ (about −120°; family III, Table 2) and, by consequence, the orientation of the R group (colored in magenta in Figure 7). In particular, the lowest energy conformational family of compounds **12–15** and **18–19** presented an anticonformation of τ_2_ (family IIIc), while that of **16** and **17** presented a *g*^+^ conformation of τ_2_ (family IIIa). Accordingly, the pyrrolidine ring of **12** occupies a region other than those occupied by the pyrrolidine rings of either NCX activators (**Neurounina-1** and compound **1**) or the NCX inhibitor **4** (Figures 7A and 8F). The same is valid for the bulkier R groups of the other derivatives presenting the same spacer, with the only important exception of compound **14**.

Interestingly, while all the other analogues were inactive, **14** resulted in an NCX activator with a peculiar activity profile. Contrarily to the other active compounds, **14** showed selectivity toward NCX1 and NCX3. Molecular volume comparisons showed that only the methyl substituent on the piperidine ring of **14** is able to partially cover the volume occupied by the pyrrolidine group on **Neurounina-1** and its active analogue **1** (Figure 8B). Therefore, even though the region to be occupied by NCX1 and NCX3 activators is not the same, it has to partially overlap to show any activity.

Moreover, **14** was found to activate NCX1 with a potency comparable to that of **Neurounina-1** and compound **1**; however, while these latter showed the same activity both in the forward and reverse mode of action of the transporter, **14** resulted more effective in activating NCX1 working in the reverse mode. On the contrary, in the case of NCX3, the compound is more effective when tested on the forward mode. Thus, according to its peculiar molecular shape (pharmacophore), **14** resulted selective for a specific conformation of NCX1 and NCX3, namely, the outward-facing (OF) bound to calcium and sodium ions, respectively (see the next paragraph). Finally, because compound **14**, contrarily to the other active derivatives, lacks the amine function, the protonation of the R group is not necessary for both NCX1 and NCX3 binding; however, this structural difference could contribute to the selectivity profile showed by the compound.

In summary, the active compounds share the same pharmacophore moieties, such as the benzodiazepinone scaffold, the nitro substituent on the benzo-fused ring, and a nitrogen-containing aliphatic ring as R group characterized by very strict steric requirements. The changing in the nature of the spacer is responsible for the different pharmacophore distances and molecular shapes, thus determining the different NCX selectivities and intrinsic activity profiles showed by **Neurounina-1**, **1**, **4**, and **14**.

### Investigation of the Putative Binding Site for **Neurounina-1** and Its Active Analogues

The atomic structure of the human NCX transporters has not been experimentally determined, with the exception of their intracellular calcium binding sites (CBD1 and CBD2) on the large cytosolic regulatory loop (f-loop) between TM5 and TM6.^32,33^ However, the structure of the homologous NCX transporter of *ArchaebacteriumMethanococcus jannaschii* (NCX_Mj) was crystallized in different conformational states of the OF orientation (Na^+^-bound, Ca^2+^-bound, apo form, open, semi-open, and occluded).^34,35^ Indeed, as demonstrated by kinetic analyses,^36−38^ under steady-state conditions, the membrane-bound NCX_Mj and its mammalian orthologues preferentially adopt the OF orientation.

Compared to human NCXs, NCX_Mj lacks the f-loop but preserves the 10 membrane-spanning segments comprising two homologous halves, which constitute the basic functional unit for ion transport.^12,36,39,40^ In particular, NCX_Mj preserves the highly conserved α1- and α2-repeats, that is, internal repeat sequences (presumably the product of intragenic duplication) located between the putative transmembrane (TM) helices TM2 and TM3 and TM7 and TM8, respectively, and involved in ion translocation and apparent ion affinity.^41−45^ Three Na^+^ and one Ca^2+^ are transported in separate steps, and the ion pocket encompasses the highly conserved α1 and α2 repeats with inverted topology, with 12 ion-coordinating residues and two helix-breaking signature sequences (on TM2C and TM7C) each containing a highly conserved proline residue (NCX_Mj P53 and P212, corresponding to P147 and P848 in hNCX1, respectively) actively involved in ion transport activities.^41−45^

**Figure 9:**
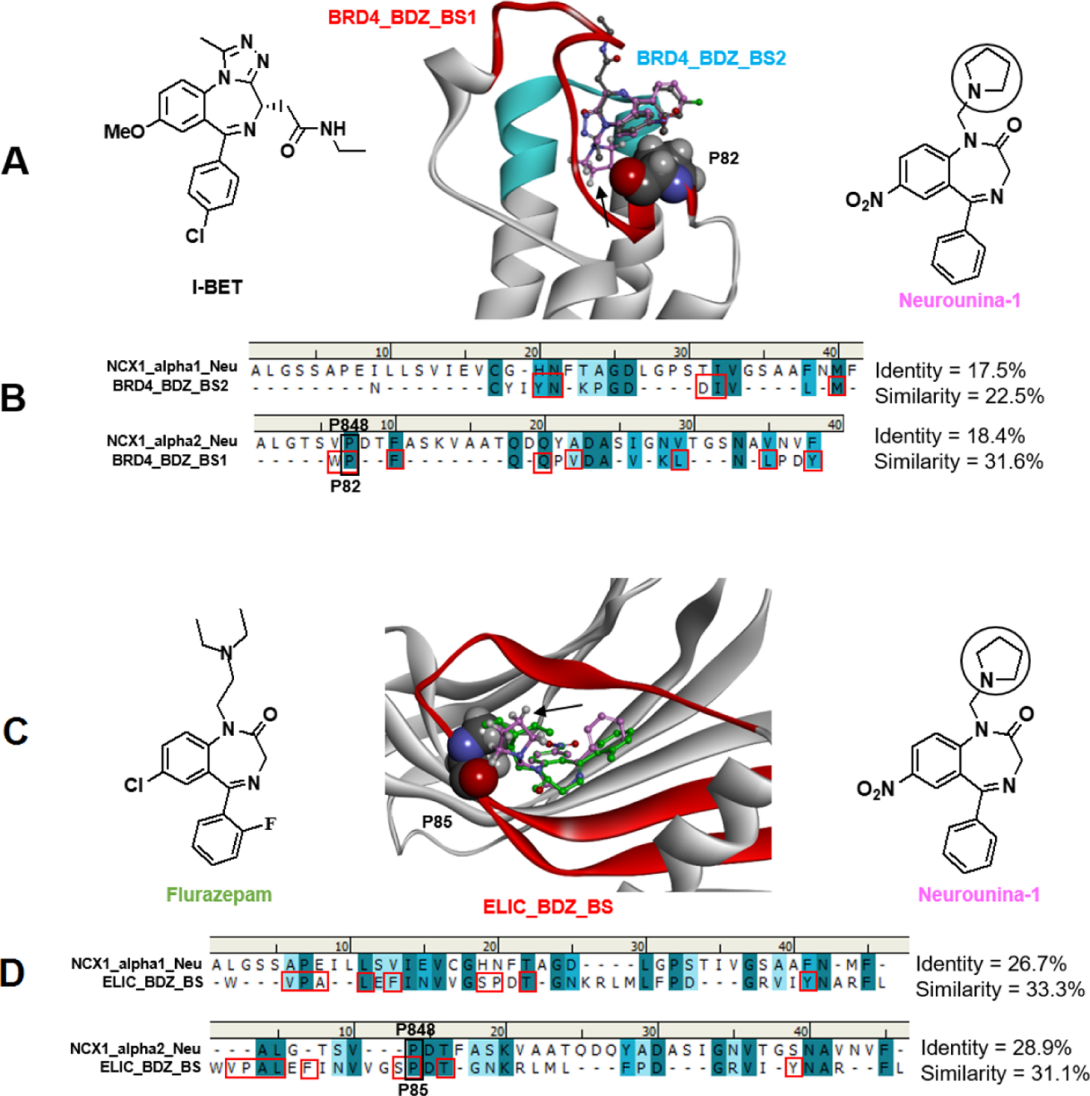
(A) Calculated **Neurounina-1** lowest energy conformation (carbons: pink) superimposed on the X-ray structure of I-BET (carbons: dark gray) in complex with the BRD4 bromodomain (PDB ID: 3P5O). The I-BET binding site is colored in red (BRD4_BDZ_BS_1) and cyan (BRD4_BDZ_BS_2). P85 is evidenced in CPK. The pyrrolidine substituent of **Neurounina-1** is indicated by a black arrow. (B) Sequence alignments of the α1 and α2 repeat regions suggested to be involved in **Neurounina-1** binding with the BRD4 alprazolam binding site. BRD4 residues establishing interactions with the benzodiazepine ligand are evidenced with red squares. (C) Calculated **Neurounina-1** lowest energy conformation (carbons: pink) superimposed on the X-ray structure of flurazepam (carbons: green) in complex with ELIC (PDB ID: 2YOE). The binding site is colored in red (ELIC_BDZ_BS). P82 is evidenced in CPK. The pyrrolidine substituent of **Neurounina-1** is indicated by a black arrow. (D) Sequence alignments of the α1 and α2 repeat regions suggested to be involved in **Neurounina-1** binding with the ELIC flurazepam binding site. ELIC residues establishing interactions with the benzodiazepine ligand are evidenced with red squares. NCX1 P848, BRD4 P82, and ELIC P85 proline residues are evidenced and labeled.

Previous mutagenesis studies^25^ allowed the identification of two regions within the NCX1 α1- and α2-repeats involved in **Neurounina-1** binding (namely, NCX1_alpha1_Neu: aa141–180 and NCX1_alpha2_Neu: aa842–879; hNCX1 numbering). Sequence alignments show that these regions are conserved in all NCX proteins and present full identity among human, dog, and rat (the species used in the present study) while presenting some differences among NCX1, NCX2, and NCX3 (Figure S4). With the aim of identifying the NCX1 putative binding site for **Neurounina-1** and its active analogues, we checked the similarity of NCX1_alpha1_Neu and NCX1_alpha2_Neu with the binding motifs contained in experimentally determined benzodiazepine binding sites by performing a structural and bioinformatic analysis (see the Experimental Section for details). Results evidenced a significant (>30%) similarity with the following: (1) the GSK525762A (BET-I) binding site (within 5 Å from the ligand) on the human BRD4 bromodomain (PDB ID: 3P5O);^46^ (2) the flurazepam binding site on the pentameric ligand-gated ion channel from *Erwinia chrysanthemi* (ELIC) (Figures 9 and S5).

In particular, the BRD4 I-BET binding site is composed by two regions: aa 81–97, herein named BDZ_BS_1 (colored in cyan in Figure 9), and aa 135–149, herein named BDZ_BS_2 (colored in red in Figure 9). BRD4_BDZ_BS1, which contains the ^81^WPF^83^ I-BET interacting motif,^46^ showed significant sequence similarity with NCX1_alpha2_Neu. Importantly, the proline residue of the ^81^WPF^83^ motif (P82) resulted aligned with NCX1 P848 (human numbering), the conserved proline residue within the signature sequence motif of the α-repeats (Figure 9).^34^

**Figure 10:**
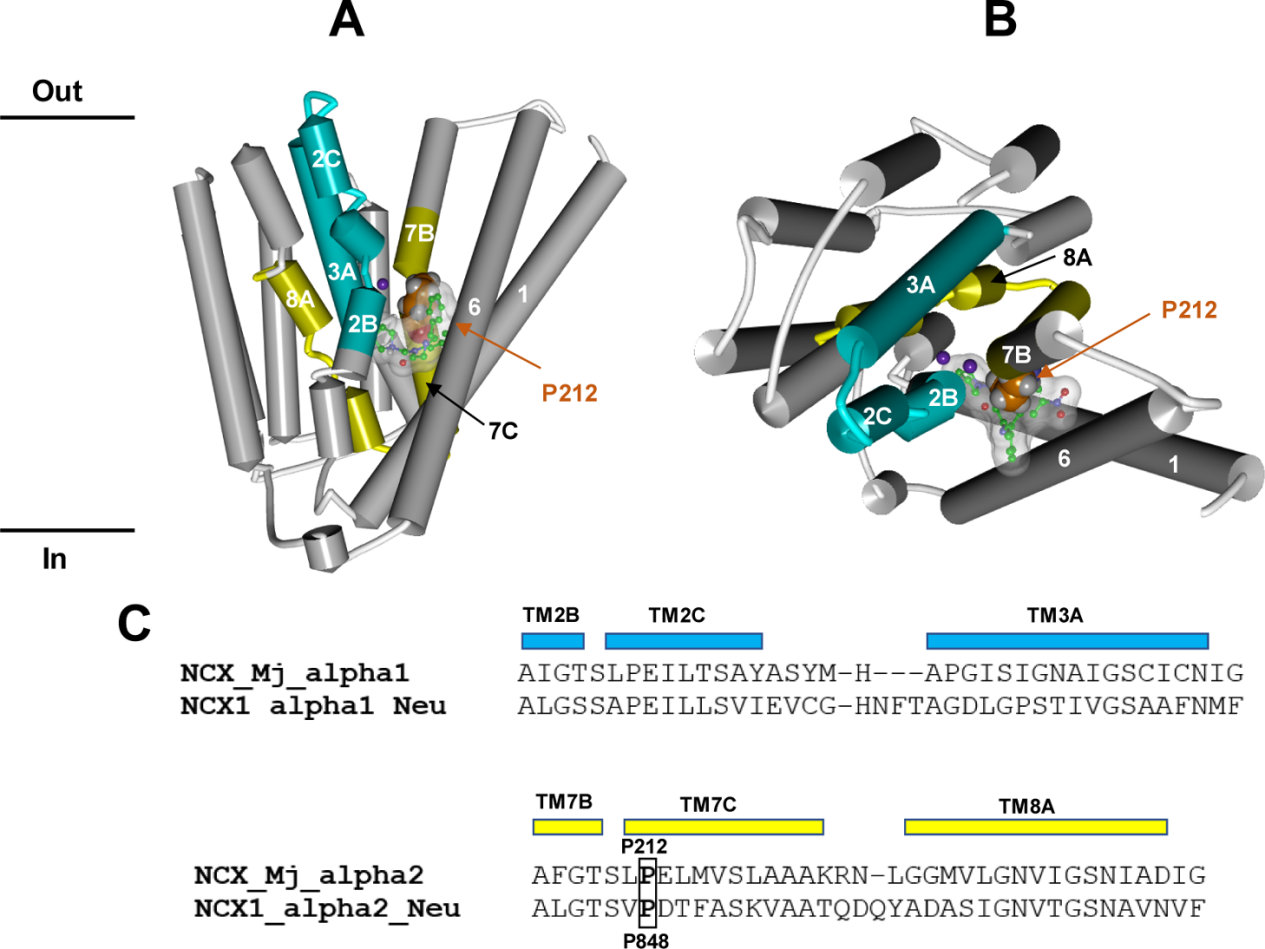
(A,B). Molecular interaction model between **Neurounina-1** and NCX_Mj resulting from our bioinformatic and structural analysis. The X-ray structure of the NCX_Mj transporter in the sodium-loaded semi-open conformation (PDB ID: 5HWY) is colored in white with NCX1_alpha1_Neu and NCX1_alpha2_Neu evidenced in cyan and yellow, respectively. The protein structure is displayed as follows: helical structures as wide cylinders, β-sheets as arrows, and coil and turn regions as tubes. The sodium atoms are displayed in ball and stick and colored in violet. The putative bioactive conformer of **Neurounina-1** is displayed in ball and stick and colored by atoms (C = green, O = red, and N = blue). **Neurounina-1** solvent accessible surface is showed and colored in white/transparent. Proline P212 is evidenced in CPK and colored in orange. (C) Sequence alignments of the α1 and α2 repeat regions of human NCX1 suggested to be involved in **Neurounina-1** binding with the corresponding segments of NCX_Mj.^31^ NCX_Mj P212 and NCX1 P848 proline residues are evidenced and labeled.

On the other hand, BRD4_BDZ_BS2 resulted best aligned with NCX1_alpha2_Neu (with which it shares a helix-loop-helix arrangement), showing the conservation of some residues contacting the benzodiazepine ligand but with an overall sequence similarity <30% (Figure 10).

The flurazepam binding site on ELIC (ELIC_BDZ_BS: aa72–107; colored in red in Figure 10) is formed by β-sheets and showed a significant sequence similarity with both NCX1_alpha1_Neu and NCX1_alpha2_Neu. It also included a proline residue (P85) in close contact with the benzodiazepine ligand (Figure 9). Interestingly, also P85 of ELIC resulted aligned with NCX1 P848, the key proline residue at the N-terminal end of the TM7B α-helix of the NCX1 transporter (α2 repeat). Moreover, the superimposition (by the common phenyl-benzodiazepine skeleton) of the energetically most favored conformer of **Neurounina-1** on the co-crystallized ligands of the BRD4/BET-I and ELIC/flurazepam complexes, places the key pyrrolidine R substituent in contact with P82 and P85, respectively (Figure 9).

The above results prompted us to speculate that the R groups of **Neurounina-1** and its active analogues could establish a Pro–Pro-like interaction with P848 on the α2-repeat of NCX transporters.

This hypothesis was supported by the very strict conformational and shape features required for the R substituent resulting from SAR analysis. Because P848 (TM7B) in human NCX1 corresponds to P212 (TM7B) in NCX_Mj (Figure 10C),^34^ we challenged our hypothesis by superimposing the heavy atoms of the P212 residue of NCX_MJ on the heavy atoms of the P84 residue of the BET–I binding motif. Strikingly, this placed the **Neurounina-1** putative bioactive conformer (previously superimposed on BET-I; Figure 9A) just behind the ion translocation pore (Figure 10A,B).

In particular, the pyrrolidine substituent resulted positioned between the conserved helix-breaking regions of TM2B-C and TM7B-C, while the 5-phenyl-1,4-benzodiazepinone scaffold was placed at the interface between TM2/TM7 and TM1/TM6.

Interestingly, other positive allosteric modulators of transporters/channels, such as Bay K 8644,^47^ citalopram,^48^ and ethanol,^49^ characterized by comparable or lower clog *P*/clog *D*_7.4_ values (Table S1), are able to bind to similar sites close to the pore center.

As evidenced by our 3-D SAR analysis, depending on the different linkers present in the structure, the R groups of **Neurounina-1** and compounds **1**, **4**, and **14** assume different orientations with respect to the benzodiazepinone skeleton. Accordingly, we have (i) NCX1 activators as **Neurounina-1** (equally potent on the reverse and forward modes)^25^ and compound **1** (more effective on the reverse mode),^27^ (ii) the NCX1 (reverse mode) inhibitor **4**, and (iii) the NCX1 (reverse mode) and NCX3 (forward mode) activator **14** (Figure 8). According to the observed pharmacological profile, the NCX1 inhibitor **4** should select/induce the OF calcium-loaded occluded conformation of the transporter (i.e., working in the reverse mode; PDB: 5HXR, Figure S6). Interestingly, the superimposition of **4** on **Neurounina-1** in the NCX_Mj (PDB: 5HXR) interaction model placed the R group of **4** at the center of the ion channel (Figure S6). On the other hand, in the case of **14**, the R group pointed more downward and resulted closer to TM7C-B compared to **Neurounina-1** (Figures S7 vs 10). NCX activators should select/induce the open state of the transporter, in particular, according to the observed pharmacological profile, **14** should preferentially bind/stabilize NCX1 in the OF calcium-loaded open conformation (i.e., working in the reverse mode; Figure S7) and NCX3 in the OF sodium-loaded open conformation (i.e., working in the forward mode).

The transition (open) state of NCX transporters has not been characterized; however, it should be situated between the two OF and inward-facing (IF) occluded states.^50^ The symmetry of NCX_Mj and its ion-binding sites, as well as the bidirectional ion exchange, suggests that the IF model should maintain similar symmetry and ion accessibility but with ion-permeation pathways directed toward the intracellular side, analogous to an inverted structure of the OF state (Figure S8).^34,51^ It was proposed that in the transition (open) state, the ion bound species stabilize the hydrophobic patch between TM2C (P53) and TM7C [P212, corresponding to P848 in human NCX1 (hNCX1)], allowing the relocation (sliding) of the gating bundle (TM1/TM6) toward the OF/IF swapping.^52^ Accordingly, we hypothesize that our compounds, by binding between TM2B-C and TM7C-B (NCX_Mj P212/hNCX1 P848) and occupying the interface between TM2/TM7 and TM1/TM6, may affect the interactions between the core domain and the gating bundle and prevent (**4**) or favor (**Neuronina-1**, **1**, and **14**) the sliding motion of this latter. In particular, our NCX activators could select/induce the open state of the transporter: (i) by stabilizing a specific relative orientation of the core domain with respect to the gating bundle corresponding to the transition (open) state of the channel or (ii) by interacting with TM2 and TM7 in place of TM1 and TM6, loosening the connection between the core and the gating bundle, and lowering the energetic barrier (Δ*G*) of the OF/IF transition. In support of this hypothesis, close to the putative binding region of **Neuronina-1** and its active analogues, we found a number of protein–protein interaction motifs (LxxLL-like and heptad repeat), whose interplay could drive the conformational transition. The identified motifs are present on TM1, TM2, TM6, and TM7 of both NCX_Mj and mammalian NCX transporters (Figure S9) and are conserved among the NCX transporters used in the experimental studies (i.e., *Canis lupus familiaris* NCX1, *Rattus* NCX2, and *Rattus* NCX3) and their human homologues.

Although it is not appropriate to speculate on the structural details underlying the NCX subtype selectivity shown by our ligands based on the experimental structure of NCX_Mj, nevertheless, some general considerations can be made. With this aim, the differences in the amino acid composition (excluding the f-loop) of the NCX isoforms used in the experimental studies were mapped on the NCX_Mj structure. Results showed that six residues are not conserved within the putative ligand binding region (within 5 Å from any ligand atom), while several residues are mutated at the interfaces between the TM domains as well as in the connecting loops (Figures S10 and S11). Similar results were also obtained considering the three human NCX isoforms (Figure S12).

Subtype selectivity of ligands which bind among TM domains is strongly affected by mutations of residues involved in interdomain interactions.^53^ Accordingly, besides the residue differences in the putative binding region, we hypothesize that the subtype selectivity of **Neurounina-1** and its active derivatives could depend on a slightly different structural arrangement of the TM domains among the three isoforms, which, in turn, could determine different ligand binding pocket shapes, similarly to what was reported for G-protein-coupled receptor ligands.^54^

In summary, the putative binding region on the NCX transporters for **Neurounina-1** and its active derivatives which resulted from our bioinformatic and structural analysis could really account for the observed pharmacological activities of the tested compounds and matched the results of our 3-D SAR study.

## Conclusions

In the present paper, we reported the synthesis and pharmacological characterization of three functional modulators of the Na^+^/Ca^2+^ exchanger (compounds **1**, **4**, and **14**), two of which are completely new (compounds **4** and **14**). From the functional point of view, compound **14** strongly activated NCX1 and NCX3 isoforms, while compound **4** selectively inhibited NCX1. Interestingly these compounds displayed a good propensity to differently modulate Ca^2+^ and Na^+^ fluxes associated with the reverse and forward modes of operation of each exchanger isoform. Accordingly, compound **4** reduced the NCX1 reverse mode associated to Ca^2+^ entrance/Na^+^ efflux with much more efficacy than the forward mode associated to Ca^2+^ extrusion/Na^+^ entrance. On the other hand, besides its highest potency in stimulating NCX1, compound **14** displayed a greater efficacy for the NCX3 forward mode. However, only compound **1** showed a neuroprotective profile in primary cortical neurons exposed to anoxic/hypoxic conditions. Therefore, we could speculate that a good stimulatory agent of NCX with a neuroprotective profile might have a greater ability to stimulate NCX1 or NCX3 reverse mode associated to Ca^2+^ entrance/Na^+^ efflux and a less ability to potentiate NCX3 forward mode associated to Ca^2+^ extrusion/Na^+^ entrance. In fact, our results suggest that the excessive activation of NCX3 forward mode determining Ca^2+^ extrusion/Na^+^ entrance may negatively counterbalance the activation of its reverse mode, favoring Ca^2+^ entrance/Na^+^ extrusion.

The results of an in-depth conformational analysis showed that the different orientations of the R group of **Neurounina-1**, **1**, **4**, and **14** could account for their different activities and selectivity profiles. Furthermore, in agreement with our 3-D SAR analysis, the predicted binding site of **Neurounina-1** and its active analogues includes a conserved proline residue responsible for key NCX conformational changes during ion translocation. The ligand-based pharmacophore model and the molecular interaction model between NCX_Mj and the analyzed active compounds provide useful tools for the design of novel and specific NCX modulators as potential neuroprotective agents. Future directions of the present study will include the homology building of full length human NCX1-3 transporters and their use in dynamic molecular docking studies in complex with **Neuronina-1** and its derivatives.

## Experimental Section

### Materials and Methods

Nitrazepam and clonazepam were purchased from F.I.S. (Fabbrica Italiana Sintetici); nordiazepam was synthesized following an already published procedure;^55^ all the reagents, solvents, or other chemicals were commercial products purchased from Sigma-Aldrich. Solutions were concentrated with a Buchi R-114 rotary evaporator at low pressure. All reactions were followed by TLC carried out on Merk Silica Gel 60 F254 plates with fluorescent indicator on the plates visualized with UV light (254 nm). Preparative chromatographic purifications were performed using the silica gel column (Kieselgel 60). Melting points, determined using a Buchi melting point B-540 instrument, are uncorrected and represent values obtained on re-crystallized or chromatographically purified material. Mass spectra of final products were recorded on a LTQ Orbitrap XL Fourier transform mass spectrometer equipped with an ESI ION MAX (Thermo Fisher, San José, USA) source operating in the positive mode. All synthesized compounds are >95% pure (elemental analysis). Elemental analyses were carried out on a Carlo Erba model 1106; analyses indicated by the symbols of the elements were within ±0.4% of the theoretical values. NMR spectra were recorded on a Bruker AVANCE Neo 700 (^1^H-700 MHz e ^13^C-175 MHz) instrument. All spectra were recorded in CDCl_3_. Chemical shifts are reported in ppm. The following abbreviations are used to describe peak patterns when appropriate: s (singlet), d (doublet), dd (double doublet), t (triplet), and m (multiplet).

#### 5-(2-Chlorophenyl)-7-nitro-1-(pyrrolidin-1-ylmethyl)-1*H*-benzo[e][1,4]diazepin-2(3*H*)-one (**1**)

The compound was prepared as already reported in our patent WO2012/072620.^56^

#### 7-Chloro-5-phenyl-1-(pyrrolidin-1-ylmethyl)-1*H*-benzo[e][1,4]diazepin-2(3*H*)-one (**2**)

The compound was prepared as already reported in our patent WO2012/072620.^56^

#### 7-Nitro-5-phenyl-1-(piperidin-1-ylmethyl)-1*H*-benzo[e][1,4]diazepin-2(3*H*)-one (**3**)

The compound was prepared as already reported in our patent WO2012/072620.^56^

#### 1-(2-Chloroethyl)-7-nitro-5-phenyl-1*H*-benzo[e][1,4]diazepin-2(3*H*)-one (**A**)

Commercially available NaH (Merck, 60% suspension in mineral oil, 427 mg, 17.8 mmol) was suspended on anhydrous DMF (40 mL) and stirred at 0 °C in a two-neck flask. A solution of nitrazepam (FIS, 5 g, 17.8 mmol) in anhydrous DMF (30 mL) was added dropwise over 15 min. After 30 min, a solution of 1-bromo-2-chloroethane (1.78 mL, 21.3 mmol) in anhydrous DMF (5 mL) was added. The mixture was stirred at room temperature for 4 h. Successively, 10 mL of H_2_O and 80 mL of ethyl acetate were added. The mixture was extracted with brine (3 × 20 mL). The organic phase was dried on anhydrous Na_2_SO_4_ and concentrated in vacuo. The residue was purified by column chromatography [dichloromethane/methanol 9.75:0.25 (v/v)]. The combined and evaporated product fractions were crystallized from diethyl ether, yielding 2.95 g of the desired intermediate **A** as an orange solid. Yield: 59%. Anal. Calcd for C_17_H_14_ClN_3_O_3_: C, 59.40; H, 4.10; N, 12.22. Found: C, 59.51; H, 4.11; N, 12.26. ^1^H NMR (CDCl_3_): δ 8.42 (dd, 1H, *J* = 2.6, 9.1), 8.22 (d, 1H, *J* = 2.6), 7.62 (d, 2H, *J* = 7.4), 7.57 (d, 1H, *J* = 9.1), 7.53 (t, 1H, *J* = 7.4), 7.45 (t, 2H, *J* = 7.4), 4.92 (d, 1H, *J* = 10.7), 4.76 (m, 1H), 4.01 (m, 1H), 3.80 (d, 1H, *J* = 10.7), 3.77 (m, 1H), 3.69 (m, 1H). ESI-MS: 344.7[M + H]^+^; 366.4 [M + Na]^+^.

#### 7-Nitro-5-phenyl-1-[2-(pyrrolidin-1-yl)ethyl]-1*H*-benzo[e][1,4]diazepin-2(3*H*)-one (**4**)

Intermediate **A** (300 mg, 0.87 mmol) and sodium iodide (197 mg; 1.31 mmol) were dissolved in acetonitrile (20 mL) and heated at 70 °C for 1 h. Then, pyrrolidine (73 μL, 63 mg, 0.87 mmol) and K_2_CO_3_ (181 mg, 1.31 mmol) were added, and the resulting suspension was stirred overnight at room temperature. The reaction mixture was filtered and dried under reduced pressure. The residue was dissolved in ethyl acetate (80 mL) and was extracted twice with brine. The organic phase was then dried over Na_2_SO_4_, filtered, and evaporated under reduced pressure. The residue was purified by silica gel column chromatography [dichloromethane/methanol, 9.75:0.25 (v/v)]. Crystallization from diethyl ether yielded 170 mg of the desired product **4** as an intense orange solid. Yield: 55%. Anal. Calcd for C_21_H_22_N_4_O_3_: C, 66.65; H, 5.86; N, 14.81. Found: C, 66.73; H, 5.87; N, 14.82. mp 70–71 °C. ^1^H NMR (CDCl_3_): δ 8.40 (dd, 1H, *J* = 2.6, 9.1), 8.19 (d, 1H, *J* = 2.6), 7.71 (d, 1H, *J* = 9.1), 7.59 (d, 2H, *J* = 7.4), 7.53 (t, 1H, *J* = 7.4), 7.45 (t, 2H, *J* = 7.4), 4.90 (d, 1H, *J* = 10.7), 4.48 (m, 1H), 3.92 (m, 1H), 3.78 (d, 1H, *J* = 10.7), 2.74 (m, 2H), 2.47 (m, 4H), 1.63 (m, 4H). ^13^C NMR (CDCl_3_):δ 169.0, 168.4, 147.9, 143.1, 137.7, 131.1, 130.5, 129.3, 128.6, 126.0, 125.8, 123.3, 56.9, 54.0, 53.5, 46.2, 23.4. ESI-MS: 379.6 [M + H]^+^; 401.6 [M + Na]^+^

#### 7-Nitro-5-phenyl-1-[2-(piperidin-1-yl)ethyl]-1*H*-benzo[e][1,4]diazepin-2(3*H*)-one (**5**)

The desired compound was obtained with the same procedure adopted for **4**, starting from intermediate **A** and piperidine. Yield: 59%. Orange solid. Anal. Calcd for C_22_H_24_N_4_O_3_: C, 67.33; H, 6.16; N, 14.28. Found: C, 66.52; H, 6.15; N, 14.32. mp 121–122 °C ^1^H NMR (CDCl_3_): δ 8.40 (dd, 1H, *J* = 2.6, 9.1), 8.20 (d, 1H, *J* = 2.6), 7.88 (d, 1H, *J* = 9.1), 7.62 (d, 2H, *J* = 7.4), 7.53 (t, 1H, *J* = 7.4), 7.45 (t, 2H, *J* = 7.4), 4.90 (d, 1H, *J* = 10.7), 4.38 (m, 1H), 3.87 (m, 1H), 3.78 (d, 1H, *J* = 10.7), 2.56 (m, 1H), 2.46 (m, 1H), 2.31 (m, 4H), 1.40 (m, 4H), 1.36 (m, 2H). ^13^C NMR (CDCl_3_): δ 168.9, 168.2, 148.1, 143.0, 137.6, 131.1, 130.1, 129.4, 128.6, 126.0, 125.7, 123.7, 57.0, 56.9, 54.6, 45.1, 25.7, 24.0. ESI-MS: 393.4 [M + H]^+^; 415.0 [M + Na]^+^.

#### 1-[2-(2-Methylpiperidin-1-yl)ethyl]-7-nitro-5-phenyl-1*H*-benzo[e][1,4]diazepin-2(3*H*)-one (**6**)

The desired compound was obtained with the same procedure adopted for **4**, starting from intermediate **A** and 2-methyl-piperidine. Yield: 61%. Orange solid. Anal. Calcd for C_23_H_26_N_4_O_3_: C, 67.96; H, 6.45; N, 13.78. Found: C, 68.16; H, 6.44; N, 13.77. mp 115–116 °C. ^1^H NMR (CDCl_3_): δ 8.40 (dd, 1H, *J* = 2.6, 9.1), 8.21 (d, 1H, *J* = 2.6), 7.89 (d, 1H, *J* = 9.1), 7.62 (d, 2H, *J* = 7.4), 7.54 (t, 1H, *J* = 7.4), 7.45 (t, 2H, *J* = 7.4), 4.90 (d, 1H, *J* = 10.7), 4.22 (m, 1H), 3.94 (m, 1H), 3.77 (d, 1H, *J* = 10.7), 2.82 (m, 2H), 2.50 (m, 1H), 2.32 (m, 1H), 2.20 (m, 1H), 1.76 (m, 2H) 1.64 (m, 2H), 1.63 (m, 2H), 0.95 (d, 3H, *J* = 5.8). ^13^C NMR (CDCl_3_): δ 168.9, 168.2, 148.0, 143.0, 137.5, 131.1, 130.1, 129.4, 128.7, 126.1, 125.7, 123.7, 57.0, 55.6, 55.2, 54.6, 47.3, 34.2, 25.7, 24.0, 18.7. ^.^ESI-MS: 407.3 [M + H].

#### 1-[2-(3-Methylpiperidin-1-yl)ethyl]-7-nitro-5-phenyl-1*H*-benzo[e][1,4]diazepin-2(3*H*)-one (**7**)

The desired compound was obtained with the same procedure adopted for **4**, starting from intermediate **A** and 3-methyl-piperidine. Yield: 60%. Orange solid. Anal. Calcd for C_23_H_26_N_4_O_3_: C, 67.96; H, 6.45; N, 13.78. Found: C, 68.38; H, 6.45; N, 13.74. mp 109–110 °C. ^1^H NMR (CDCl_3_): δ 8.37 (dd, 1H, *J* = 2.6, 9.1), 8.20 (d, 1H, *J* = 2.6), 7.85 (d, 1H, *J* = 9.1), 7.60 (d, 2H, *J* = 7.4), 7.51 (t, 1H, *J* = 7.4), 7.42 (t, 2H, *J* = 7.4), 4.88 (d, 1H, *J* = 10.7), 4.37 (m, 1H), 3.87 (m, 1H), 3.75 (d, 1H, *J* = 10.7), 2.68 (overlapped, 2H), 2.54 (m, 1H), 2.46 (m, 1H), 1.85 (m, 1H), 1.78 (m, 2H), 1.58 (m, 2H), 1.51 (m, 1H), 1.35 (m, 1H), 0.75 (d, 3H, *J* = 6.5). ^13^C NMR (CDCl_3_): δ 168.9, 168.3, 148.1, 143.1, 137.7, 131.1, 130.1, 129.4, 128.7, 126.1, 125.7, 123.7, 61.9, 57.0, 54.2, 53.8, 45.1, 32.6, 31.0, 25.3, 19.6. ESI-MS: 407.3 [M + H]^+^.

#### 1-[2-(1*H*-Imidazole-1-yl)ethyl]-7-nitro-5-phenyl-1*H*-benzo[e][1,4]diazepin-2(3*H*)-one (**8**)

The desired compound was obtained with the same procedure adopted for **4**, starting from intermediate **A** and imidazole. Yield: 63%. Orange solid. Anal. Calcd for C_20_H_17_N_5_O_3_: C, 63.99; H, 4.56; N, 18.66. Found: C, 64.20; H, 4.56; N, 18.70. mp 102–103 °C. ^1^H NMR (CDCl_3_): δ 8.28 (dd, 1H, *J* = 2.6, 9.1), 8.14 (d, 1H, *J* = 2.6), 7.55–7.44 (overlapped, 6H), 7.02 (d, 1H *J* = 9.1), 6.87 (s, 1H), 6.74 (s, 1H), 4.94 (d, 1H, *J* = 10.7), 4.50 (m, 1H), 4.29 (m, 2H), 4.08 (m, 1H), 3.74 (d, 1H, *J* = 10.7). ^13^C NMR (CDCl_3_): δ 169.2, 169.1, 147.5, 143.4, 136.9, 131.6, 131.2, 129.9, 129.6, 129.4, 128.7, 126.4, 126.3, 122.3, 56.8, 50.2, 44.8. ESI-MS: 376.3 [M + H]^+^.

#### 1-[2-(2-Methyl-1*H*-imidazole-1-yl)ethyl]-7-nitro-5-phenyl-1*H*-benzo[e][1,4]diazepin-2(3*H*)-one (**9**)

The desired compound was obtained with the same procedure adopted for **4**, starting from intermediate **A** and 2-methyl-imidazole. Yield: 64%. Orange solid. Anal. Calcd for C_21_H_19_N_5_O_3_: C, 64.77; H, 4.92; N, 17.98. Found: C, 64.90; H, 4.93; N, 18.02. mp 96–97 °C. ^1^H NMR (CDCl_3_): δ 8.32 (dd, 1H, *J* = 2.6, 9.1), 8.15 (d, 1H, *J* = 2.6), 7.55–7.44 (overlapped, 5H), 7.04 (d, 1H *J* = 9.1), 6.73 (s, 1H), 6.63 (s, 1H), 4.93 (d, 1H, *J* = 10.7), 4.39 (m, 1H), 4.21 (t, 2H, *J* = 6.2), 4.06 (m, 1H), 3.74 (d, 1H, *J* = 10.7), 2.26 (s, 3H). ^13^C NMR (CDCl_3_): δ 169.2, 169.1, 147.5, 144.4, 143.4, 136.9, 131.6, 131.2, 129.9, 129.6, 129.4, 128.7, 126.4, 126.3, 122.4, 119.0, 56.8, 49.7, 43.6, 12.7. ESI-MS: 390.3 [M + H]^+^; 412.2 [M + Na]^+^.

#### 7-Nitro-5-phenyl-1-[2-(4-phenylpiperazin-1-yl)ethyl]-1*H*-benzo[e][1,4]diazepin-2(3*H*)-one (**10**)

The desired compound was obtained with the same procedure adopted for **4**, starting from intermediate **A** and 1-phenylpiperazine. Yield: 63%. Orange solid. Anal. Calcd for C_27_H_27_N_5_O_3_: C, 69.07; H, 5.80; N, 14.92. Found: C, 68.83; H, 5.78; N, 14.93. mp 91–92 °C. ^1^H NMR (CDCl_3_): δ 8.38 (dd, 1H, *J* = 2.6, 9.1), 8.21 (d, 1H, *J* = 2.6), 7.76 (d, 1H, *J* = 9.1), 7.60 (d, 2H, *J* = 7.4), 7.48 (t, 1H, *J* = 7.4), 7.38 (t, 2H, *J* = 7.4), 7.22 (t, 2H, *J* = 7.4), 6.83 (t, 1H, *J* = 7.4), 6.80 (d, 2H, *J* = 7.4), 4.90 (d, 1H, *J* = 10.7), 4.52 (m, 1H), 3.85 (m, 1H), 3.77 (d, 1H, *J* = 10.7), 2.92 (m, 4H), 2.65–2.40 (overlapped, 6H). ^13^C NMR (CDCl_3_): δ168.7, 168.5, 151.1, 147.8, 143.2, 137.4, 131.2, 130.4, 129.4, 129.0, 128.6, 126.2, 125.8, 123.4, 119.8, 116.0, 57.0, 56.0, 53.2, 48.8, 44.5. ESI-MS: 470.2 [M + H]^+^.

#### 1-{2-[4-(Naphthalen-1-yl)piperazin-1-yl]ethyl}-7-nitro-5-phenyl-1*H*-benzo[e][1,4]diazepin-2(3*H*)-one (**11**)

The desired compound was obtained with the same procedure adopted for **4**, starting from intermediate **A** and 1-(1-naphthyl)piperazine hydrochloride. Yield: 60%. Orange solid. Anal. Calcd for C_31_H_29_N_5_O_3_: C, 71.66; H, 5.63; N, 13.48. Found: C, 71.76; H, 5.65; N, 13.52. mp 110–111 °C.^1^H NMR (CDCl_3_): δ 8.40 (dd, 1H, *J* = 2.6, 9.1), 8.23 (d, 1H, *J* = 2.6), 8.08 (m, 1H), 7.80–7.79 (m, 2H), 7.65 (m, 2H), 7.55–7.40 (overlapped, 6H), 7.35 (t, 1H, *J* = 7.4), 6.83 (d, 1H, *J* = 7.4), 4.91 (d, 1H, *J* = 10.7), 4.58 (m, 1H), 3.88 (m, 1H), 3.79 (d, 1H, *J* = 10.7), 2.87 (m, 4H), 2.67 (m, 2H), 2.57 (m, 4H). ^13^C NMR (CDCl_3_): δ 168.7, 168.5, 149.4, 147.8, 143.2, 137.5, 134.7, 131.3, 130.5, 129.6, 128.8, 128.7, 128.4, 126.2, 125.8, 125.3, 123.5, 123.4, 114.7, 57.1, 56.0, 53.7, 52.6, 44.4. ESI-MS: 520.3 [M + H]^+^.

#### *tert*-Butyl-2-(7-nitro-2-oxo-5-phenyl-2,3-dihydro-1*H*-benzo[e][1,4]diazepin-1-yl)-acetate (**B**)

Commercially available NaH (Merck, 60% suspension in mineral oil, 427 mg, 17.8 mmol) was suspended on anhydrous DMF (40 mL) and stirred at 0 °C in a two-neck flask. A solution of nitrazepam (FIS, 5 g, 17.8 mmol) in anhydrous DMF (30 mL) was added dropwise over 15 min. After 30 min, a solution of *tert*-butyl bromoacetate (3.16 mL, 21.4 mmol) in anhydrous DMF (5 mL) was added dropwise. The mixture was stirred at room temperature for 4 h. Successively, 10 mL of H_2_O and 80 mL of ethyl acetate were added. The mixture was extracted with brine (3 × 20 mL). The organic phase was dried on anhydrous Na_2_SO_4_ and concentrated in vacuo. The residue was purified by column chromatography [*n*-hexane/ethyl acetate 5:5 (v/v)]. The combined and evaporated product fractions were crystallized from diethyl ether, yielding 3.87 g of the desired intermediate **B** as a yellow pale solid. Yield: 59%. Anal. Calcd for C_21_H_21_N_3_O_5_: C, 63.79; H, 5.35; N, 10.63. Found: C, 64.03; H, 5.35; N, 10.67. ^1^H NMR (CDCl_3_): δ 8.40 (dd, 1H, *J* = 2.6, 9.1), 8.23 (d, 1H, *J* = 2.6), 7.62 (d, 2H, *J* = 7.4), 7.53 (t, 1H, *J* = 7.4), 7.45 (overlapped, 2H), 7.44 (overlapped, 1H), 4.97 (d, 1H, *J* = 10.7), 4.65 (d, 1H, *J* = 17.2), 4.33 (d, 1H, *J* = 17.2), 3.88 (d, 1H, *J* = 10.7), 1.50 (s, 9H). ESI-MS: 396.3[M + H]^+^; 418 [M + Na]^+^.

#### 2-(7-Nitro-2-oxo-5-phenyl-2,3-dihydro-1*H*-benzo[e][1,4]diazepin-1-yl)acetic Acid (**C**)

Intermediate **B** (3.5 g, 8.86 mmol) was reacted with a solution of trifluoroacetic acid in dichloromethane (2:8, v/v, 20 mL) for 1 h. The reaction mixture was then added with diethyl ether (60 mL) and the obtained precipitate was collected by centrifugation and dried, obtaining 2.95 g of intermediate **C** as an intense yellow solid. Yield: 98%. Anal. Calcd for C_17_H_13_N_3_O_5_: C, 60.18; H, 3.86; N, 12.38. Found: C, 60.02; H, 3.85; N, 12.37. ^1^H NMR (CDCl_3_): δ 8.45 (dd, 1H, *J* = 2.6, 9.1), 7.98 (d, 1H, *J* = 2.6), 7.76 (d, 1H, *J* = 9.1), 7.59 (d, 2H, *J* = 7.4), 7.53 (t, 1H, *J* = 7.4), 7.48 (t, 2H, *J* = 7.4), 4.69 (d, 1H, *J* = 10.7), 3.92 (d, 1H, *J* = 10.7), 2.50 (m, 2H). ESI-MS: 340.2 [M + H]^+^; 362.1 [M + Na]^+^.

#### 7-Nitro-1-[2-Oxo-2-(pyrrolidin-1-yl)ethyl]-5-phenyl-1*H*-benzo[e][1,4]diazepin-2(3*H*)-one (**12**)

Intermediate **C** (300 mg, 0.88 mmol) was dissolved in dry DMF (20 mL). TBTU (311 mg, 0.97 mmol), HOBt (131 mg, 0.97 mmol), pyrrolidine (73 μL, 63 mg, 0.88 mmol), and DIPEA (317 μL, 239 mg, 1.85 mmol) were added, and the resulting solution was stirred overnight at room temperature. The reaction mixture was dried under reduced pressure and the residue dissolved in ethyl acetate (80 mL) was extracted twice with 5% NaHCO_3_ (20 mL), 10% citric acid (20 mL), and brine. The organic phase was then dried over Na_2_SO_4_, filtered, and evaporated under reduced pressure. The residue was purified by silica gel column chromatography [*n*-hexane/ethyl acetate 2:8 (v/v)]. Crystallization from diethyl ether yielded 190 mg of the desired product **12** as an intense yellow solid. Yield: 55%. Anal. Calcd for C_21_H_20_N_4_O_4_: C, 64.28; H, 5.14; N, 14.28. Found: C, 64.48; H, 5.15; N, 14.31. mp 209–210 °C. ^1^H NMR (CDCl_3_): δ 8.34 (dd, 1H, *J* = 2.6, 9.1), 8.20 (d, 1H, *J* = 2.6), 7.70 (d, 1H, *J* = 9.1), 7.60 (d, 2H, *J* = 7.4), 7.51 (t, 1H, *J* = 7.4), 7.43 (t, 2H, *J* = 7.4), 4.93 (d, 1H, *J* = 10.7), 4.81 (d, 1H, *J* = 16.4), 4.27 (d, 1H, *J* = 16.4), 3.90 (d, 1H, *J* = 10.7), 3.61 (m, 1H), 3.58 (m, 1H), 3.50 (m, 1H), 3.43 (m, 1H), 2.04 (m, 1H), 2.00 (m, 1H), 1.89 (m, 2H). ^13^C NMR (CDCl_3_): δ 169.2, 168.8, 165.1, 148.3, 143.1, 137.9, 131.1, 129.4, 129.3, 128.6, 126.1, 126.0, 123.0, 56.7, 50.6, 46.3, 46.0, 26.2, 24.1. ESI-MS: 393.2 [M + H]^+^; 415.2[M + Na]^+^.

#### 7-Nitro-1-[2-Oxo-2-(piperidin-1-yl)ethyl]-5-phenyl-1*H*-benzo[e][1,4]diazepin-2(3*H*)-one (**13**)

The desired compound was obtained with the same coupling procedure adopted for **12**, starting from intermediate **C** and piperidine. Yield: 63%. Yellow solid. Anal. Calcd for C_22_H_22_N_4_O_4_: C, 65.01; H, 5.46; N, 13.78. Found: C, 64.89; H, 5.46; N, 13.81. mp 125–126 °C. ^1^H NMR (CDCl_3_): δ 8.33 (dd, 1H, *J* = 2.6, 9.1), 8.20 (d, 1H, *J* = 2.6), 7.60 (d, 2H, *J* = 7.4), 7.53 (d, 1H, *J* = 9.1), 7.50 (t, 1H, *J* = 7.4), 7.42 (t, 2H, *J* = 7.4), 4.92 (overlapped, 2H), 4.39 (d, 1H, *J* = 16.4), 3.89 (d, 1H, *J* = 10.7), 3.61 (m, 1H), 3.54 (m, 1H), 3.44 (m, 1H), 3.39 (m, 1H), 1.68 (m, 1H), 1.66 (m, 2H), 1.59 (overlapped, 3H). ^13^C NMR (CDCl_3_): δ 169.2, 168.8, 164.6, 148.2, 143.0, 137.9, 131.0, 129.5, 129.4, 128.6, 126.1, 126.0, 122.6, 56.8, 49.5, 46.3, 43.6, 26.3, 25.5, 24.3. ESI-MS: 407.3 [M + H]^+^.

#### 1-[2-(2-Methylpiperidin-1-yl)-2-oxoethyl]-7-nitro-5-phenyl-1*H*-benzo[e][1,4]-diazepin-2(3*H*)-one (**14**)

The desired compound was obtained with the same coupling procedure adopted for **12**, starting from intermediate **C** and 2-methyl-piperidine. Yield: 51%. Yellow solid. Anal. Calcd for C_23_H_24_N_4_O_4_: C, 65.70; H, 5.75; N, 13.33. Found: C, 65.56; H, 5.74; N, 13.31. mp 126–127 °C. ^1^H NMR (CDCl_3_): δ 8.33 (dd, 1H, *J* = 2.6, 9.1), 8.19 (d, 1H, *J* = 2.6), 7.60 (d, 2H, *J* = 7.4), 7.55 (d, 1H, *J* = 9.1), 7.50 (t, 1H, *J* = 7.4), 7.43 (t, 2H, *J* = 7.4), 4.94 (overlapped, 1H), 4.93 (overlapped, 1H), 4.90 (overlapped, 1H), 4.88 (overlapped, 1H), 4.40 (m, 1H), 4.33 (m, 1H), 3.90 (d, 1H, *J* = 10.7), 1.73 (overlapped, 1H), 1.68 (overlapped, 2H), 1.66 (overlapped, 2H), 1.43 (m, 1H), 1.18 (d, 3H, *J* = 5.6). ^13^C NMR (CDCl_3_): δ 169.3, 168.9, 164.9, 148.4, 143.0, 137.9, 131.0, 129.5, 129.4, 128.6, 126.1, 126.0, 122.5, 56.7, 49.9, 48.3, 44.8, 29.7, 25.3, 18.6, 18.5. ESI-MS: 421.3 [M + H]^+^.

#### 1-3-[(3-Methylpiperidin-1-yl)-2-oxoethyl]-7-nitro-5-phenyl-1*H*-benzo[e][1,4]-diazepin-2(3*H*)-one (**15**)

The desired compound was obtained with the same coupling procedure adopted for **12**, starting from intermediate **C** and 3-methyl-piperidine. Yield: 58%. Yellow solid. Anal. Calcd for C_23_H_24_N_4_O_4_: C, 65.70; H, 5.75; N, 13.33. Found: C, 65.58; H, 5.75; N, 13.34. mp 119–120 °C. ^1^H NMR (CDCl_3_): δ 8.33 (dd, 1H, *J* = 2.6, 9.1), 8.20 (d, 1H, *J* = 2.6), 7.60 (d, 2H, *J* = 7.4), 7.54 (d, 1H, *J* = 9.1), 7.50 (t, 1H, *J* = 7.4), 7.43 (t, 2H, *J* = 7.4), 4.93 (overlapped, 1H), 4.91 (overlapped, 1H), 4.34 (m, 1H), 3.90 (d, 1H, *J* = 10.7), 3.87 (m, 1H), 3.62 (m, 2H), 2.34 (m, 1H), 1.79 (m, 1H), 1.66 (m, 1H), 1.53 (m, 1H), 1.47 (overlapped, 1H), 1.45 (overlapped, 1H), 0.91 (d, 3H, *J* = 5.6). ^13^C NMR (CDCl_3_): δ 169.3, 168.9, 164.7, 148.2, 143.1, 137.9, 131.0, 129.5, 129.4, 128.6, 126.1, 126.0, 122.5, 56.7, 49.7, 46.0, 43.3, 31.0, 24.7, 22.7, 19.0. ESI-MS: 421.3 [M + H]^+^.

#### 1-[2-(1*H*-Imidazole-1-yl)-2-oxoethyl]-7-nitro-5-phenyl-1*H*-benzo[e][1,4]diazepin-2(3*H*)-one (**16**)

Intermediate **C** (300 mg, 0.88 mmol) was dissolved in anhydrous dichloromethane (10 mL) in a two-neck flask. An excess of SOCl_2_ (642 μL; 8.80 mmol) was added. The mixture was heated at reflux for 1 h. The solvent was evaporated under reduced pressure. The light yellow residue was dissolved in dichloromethane and re-evaporated three times. The residue was used in the next step without further purification. The acyl chloride was obtained as a light yellow powder and was dissolved in anhydrous tetrahydrofuran (10 mL). Imidazole (120 mg, 1.66 mmol) was added, and the reaction was stirred overnight at room temperature. The reaction resulted in the precipitation of imidazolium chloride. The mixture was filtered and the solvent was evaporated under reduced pressure, yielding the crude product. Crystallization from diethyl ether and *n*-hexane yielded 149 mg of the desired product **16** as an intense yellow solid. Yield: 48%. Anal. Calcd for C_20_H_15_N_5_O_4_: C, 61.69; H, 3.88; N, 17.99. Found: C, 61.83; H, 3.89; N, 18.04. mp 120–121 °C. ^1^H NMR (CDCl_3_): δ 8.33 (dd, 1H, *J* = 2.6, 9.1), 8.20 (d, 1H, *J* = 2.6), 7.60 (d, 2H, *J* = 7.4), 7.53 (t, 1H, *J* = 7.4), 7.40 (t, 2H, *J* = 7.4), 7.31 (s, 1H), 7.02 (d, 1H *J* = 9.1), 6.72 (s, 1H), 6.63 (s, 1H), 4.92 (d, 1H, *J* = 10.7), 4.85 (d, 1H, *J* = 16.7), 4.38 (d, 1H, *J* = 16.7) 3.78 (d, 1H, *J* = 10.7). ^13^C NMR (CDCl_3_): δ 169.2, 168.8, 165.1, 148.3, 143.2, 137.8, 136.4, 131.0, 129.5, 129.4, 128.7, 126.1, 126.0, 123.0, 116.2, 56.6, 50.4. ESI-MS: 390.3 [M + H]^+^.

#### 1-[2-(2-Methyl-1H-imidazole-1-yl)-2-oxoethyl]-7-nitro-5-phenyl-1*H*-benzo[e][1,4]diazepin-2(3*H*)-one (**17**)

The desired compound was obtained with the same coupling procedure adopted for **16**, starting from intermediate **C** and 2-methyl-imidazole. Yield: 54%. Yellow solid. Anal. Calcd for C_21_H_17_N_5_O_4_: C, 62.53; H, 4.25; N, 17.36. Found: C, 62.37; H, 4.26; N, 17.41. mp 113–114 °C. ^1^H NMR (CDCl_3_): δ 8.34 (dd, 1H, *J* = 2.6, 9.1), 8.19 (d, 1H, *J* = 2.6), 7.60 (d, 2H, *J* = 7.4), 7.53 (t, 1H, *J* = 7.4), 7.40 (t, 2H, *J* = 7.4), 7.02 (d, 1H *J* = 9.1), 6.72 (s, 1H), 6.63 (s, 1H), 4.90 (overlapped 1H), 4.91 (overlapped 1H), 4.39 (d, 1H, *J* = 16.7), 3.78 (d, 1H, *J* = 10.7), 2.26 (s, 3H). ^13^C NMR (CDCl_3_): δ 169.2, 168.8, 165.4, 155.7, 148.3, 143.2, 137.8, 131.1, 130.9, 129.5, 129.4, 128.7, 126.1, 123.2, 117.6, 56.6, 50.2, 16.9. ESI-MS: 404.2 [M + H]^+^.

#### 7-Nitro-1-[2-Oxo-2-(4-phenylpiperazin-1-yl)ethyl]-5-phenyl-1*H*-benzo[e][1,4]diazepin-2(3*H*)-one (**18**)

The desired compound was obtained with the same coupling procedure adopted for **12**, starting from intermediate **C** and 1-phenylpiperazine. Yield: 64%. Yellow solid. Anal. Calcd for C_27_H_25_N_5_O_4_: C, 67.07; H, 5.21; N, 14.48. Found: C, 67.21; H, 5.21; N, 14.53. mp 153–154 °C. ^1^H NMR (CDCl_3_): δ 8.38 (dd, 1H, *J* = 2.6, 9.1), 8.22 (d, 1H, *J* = 2.6), 7.60 (overlapped, 1H), 7.60 (overlapped, 2H), 7.52 (t, 1H, *J* = 7.4), 7.45 (t, 2H, *J* = 7.4), 7.30 (t, 2H, *J* = 7.4), 6.94 (overlapped, 1H), 6.93 (overlapped, 2H), 4.96 (overlapped, 1H), 4.95 (overlapped, 1H), 4.52 (d, 1H, *J* = 16.7), 3.93 (d, 1H, *J* = 10.7), 3.83 (m, 2H), 3.71 (m, 1H), 3.66 (m, 1H), 3.29 (m, 1H), 3.22 (m, 3H). ^13^C NMR (CDCl_3_): δ 169.3, 168.8, 150.7, 148.0, 137.8, 131.2, 131.2, 129.7, 129.5, 129.3, 128.7, 126.2, 126.1, 122.6, 120.8, 116.8, 56.6, 49.6, 49.4, 49.3, 44.5, 42.4. ESI-MS: 484.3 [M + H]^+^.

#### 1-{2-[4-(Naphthalen-1-yl)piperazin-1-yl]-2-oxoethyl}-7-nitro-5-phenyl-1*H*-benzo[e][1,4]-diazepin-2(3*H*)-one (**19**)

The desired compound was obtained with the same coupling procedure adopted for **12**, starting from intermediate **C** and 1-(1-naphthyl)-piperazine hydrochloride. Yield: 59%. Yellow solid. Anal. Calcd for C_31_H_27_N_5_O_4_: C, 69.78; H, 5.10; N, 13.13; Found: C, 67.60; H, 5.09; N, 13.15. mp 178–179 °C.^1^H NMR (CDCl_3_): δ 8.40 (dd, 1H, *J* = 2.6, 9.1), 8.23 (d, 1H, *J* = 2.6), 8.18 (d, 1H, *J* = 7.4), 7.87 (d, 1H, *J* = 7.4), 7.64 (overlapped, 2H), 7.63 (overlapped, 2H), 7.54 (overlapped, 1H), 7.52 (overlapped, 2H), 7.47 (t, 2H, *J* = 7.4), 7.42 (t, 1H, *J* = 7.4), 7.05 (s, 1H), 4.99 (overlapped, 1H), 4.98 (overlapped, 1H), 4.58 (m, 1H), 3.95 (d, 1H, *J* = 10.7), 3.82 (m, 4H), 3.22 (m, 4H). ^13^C NMR (CDCl_3_): δ169.3, 168.9, 165.3, 148.6, 148.0, 143.2, 137.8, 134.7, 131.3, 129.7, 129.5, 128.8, 128.7, 128.6, 126.2, 126.1, 126.0, 125.8, 125.7, 124.3, 123.0, 122.7, 115.2, 56.7, 52.9, 52.7, 49.4, 45.8, 43.1. ESI-MS: 534.3 [M + H]^+^.

### Biological Tests

#### Cell Cultures

##### Stably Transfected Baby Hamster Kidney Cells

Baby hamster kidney (BHK) cells stably expressing dog cardiac NCX1 and NCX3 were grown as previously reported.^57^ For video-imaging and electrophysiological studies, cells were seeded on glass coverslips coated with poly-l-lysine (Sigma, St. Louis, MO).

##### Rat Primary Cortical Neurons

Rat cortical neurons were prepared and grown as previously reported.^22,23^ During the exposure to hypoxic conditions, primary cortical neurons at 7/10 DIV were incubated with different concentrations of each previously identified compound. Italian Ministry of Health and the local Animal Care Committee of “Federico II” University of Naples (Italy) approved all animal procedures adopted (D. Lgs. March 4th, 2014 from Italian Ministry of Health; DIR 210/63 UE; 12/2018-UT7).

#### High-Throughput Screening to Test the Biological Activity with Fluorescent Probes

High-throughput assay was aimed to screen libraries of compounds for their activity on the NCX reverse mode. This analysis was performed by microplate screening applications in semi-automated experiments measuring changes in the intracellular Ca^2+^ level after the addition of Na^+^-free solution to BHK-NCX1 and BHK-NCX3 cells loaded with Fluo-4AM or Fura 2AM. Before testing the compounds, cells were separately loaded with Fluo-4 AM or Fura 2AM (10 μM each for 30′ at 37 °C). Identification of newly synthesized compounds enhancing NCX reverse mode or inhibiting NCX activity was done by measuring their ability to increase, or decrease, the Na^+^-free-dependent Ca^2+^ level above the mean of basal value of almost ±2 S.D.

#### [Ca^2+^]_*i*_ Measurement on Single Cell

[Ca^2+^]_*i*_ was measured by single-cell computer-assisted video-imaging using Fura-2 acetoxymethyl ester (Fura-2AM). Neurons were loaded with Fura 2AM (10 μM for 30′ at 37 °C). Experiments were carried out with a MicroMax 512BFT cooled CCD camera (Princeton Instruments, Trenton, NJ, USA), LAMBDA 10-2 filter wheeler (Sutter Instruments, Novato, CA), and Meta-Morph/MetaFluor Imaging System software (Universal Imaging, West Chester, PA). Fura-2 fluorescence intensity was measured every 3 s. From each coverslip, 40–60 individual cells were selected and monitored simultaneously. Assuming that the KD for FURA-2 was 224 nM, the equation of Grynkiewicz was used to for calibration.^58^

The NCX reverse mode was determined by switching the normal Krebs medium to Na^+^-deficient NMDG+ medium (Na^+^-free), as previously reported.^57^

#### Patch-Clamp Electrophysiology

All currents were recorded by patch-clamp technique in the whole-cell configuration using a Digidata 1322A interface (Molecular Devices). Data were acquired and analyzed using pClamp software (version 9.0, Molecular Devices). The currents were recorded by fire-polished borosilicate electrodes with a final resistance of 2.5–4 MΩ filled with a specific internal solution.

NCX currents filtered at 5 kHz were recorded from BHK wild type (Wt), BHK-NCX1, and BHK-NCX3 stably transfected cells.^29^ Briefly, NCX currents were recorded starting from a holding potential of −60 mV up to a short-step depolarization at +60 mV (60 ms).^15,29^ Then, a descending voltage ramp from +60 to −120 mV was applied. The current recorded in the descending portion of the ramp (from +60 to −120 mV) was used to plot the current–voltage (*I*–*V*) relation curve. The magnitudes of NCX currents were measured at the end of +60 mV (reverse mode) and at the end of −120 mV (forward mode), respectively.

The Ni^2+^-insensitive components were subtracted from total currents to isolate NCX currents. The external Ringer solution contained (in mM) the following: 126 NaCl, 1.2 NaHPO_4_, 2.4 KCl, 2.4 CaCl_2_, 1.2 MgCl_2_, 10 glucose, and 18 NaHCO_3_ adjusted to pH 7.4 with NaOH. Moreover, 20 mM tetraethylammonium (TEA), 10 nM tetrodotoxin (TTX), and 10 μM nimodipine were added to the external solution to block delayed outward rectifier KV, L-type CaV, and TTX-sensitive NaV channels. The dialyzing pipette solution contained (mM) the following: 100 K-gluconate, 10 TEA, 20 NaCl, 1 Mg-ATP, 0.1 CaCl_2_, 2 MgCl_2_, 0.75 EGTA, and 10 HEPES, adjusted to pH 7.2 with CsOH. TEA and Cs were included in the dialyzing solution to block delayed outward rectifier K^+^ components. The values of NCX currents were normalized for membrane capacitance.

#### Oxygen and Glucose Deprivation

Hypoxic conditions were induced by exposing rat primary cortical neurons to a oxygen- and glucose-free medium in a humidified atmosphere containing 95% nitrogen and 5% CO_2_, as previously reported.^22,23^ Cell injury was assessed by MTT analysis. Briefly, neurons were incubated with 1 mL of MTT solution (0.5 mg/mL in PBS). After 1 h of incubation at 37 °C, neurons were dissolved in dimethyl sulfoxide and the rate of MTT variation was measured spectrophotometrically at the wavelength of 540 nm.^22,57^

#### Chemical Hypoxia and RX

Chemical hypoxia was reproduced by exposing primary cortical neurons for 45 min to a glucose-free medium containing oligomycin (5 μg/mL) plus 2-DG (2 mM), followed by RX (3 h). The hypoxic medium was composed of (in mM) 145 NaCl, 5.5 KCl, 1.2 MgCl_2_, 1.5 CaCl_2_, and 10 Hepes, pH 7.4. Control neurons were exposed to the neuronal growth medium. Cell injury was assessed by MTT analysis at the end of each experiment.

#### Drugs, Statistical Analysis, and Determination of EC_50_’s and IC_50_

Compounds were solubilized in dimethyl sulfoxide at concentrations of 1 mM, and stock solutions were kept at −20 °C. Appropriate drug dilutions were prepared daily. Statistical analysis was performed with two-way ANOVA, followed by the Newman–Keuls test. Statistical significance was accepted at the 95% confidence level (*p* < 0.05). Values are expressed as means ± SEM. To obtain EC_50_’s of newly synthesized compounds and IC_50_ of compound 4, all data were fitted to the following binding isotherm: *y* = max/(11*X*/IC_50_ or EC_50_)_*n*_, where *X* is the drug concentration and *n* is the Hill coefficient.

#### Molecular Modeling

Molecular modeling calculations were performed on an E4 Server Twin 2 x Dual Xeon-5520, equipped with two nodes. Each node: 2 x Intel Xeon QuadCore E5520-2.26GHz, 36 GB RAM. The molecular modeling graphics were carried out on a personal computer equipped with an Intel Core (TM) i7-4790 processor and SGI Octane 2 workstations.

The apparent p*K*_a_ and log *D* values (pH 7.4 and 7.2) of all compounds were calculated by using the ACD/p*K*_a_ classic algorithm of ACD/Percepta software (ACD/Percepta, Advanced Chemistry Development, Inc., Toronto, ON, Canada, 2017, http://www.acdlabs.com). Then, the percentage of neutral/ionized forms was computed at pH 7.4 (blood pH value) and pH 7.2 (cytoplasm pH value) using the Handerson–Hasselbalch equation. The compounds were considered in their prevalent (>50%) ionic form at the considered pHs in the subsequent calculations.

BIOVIA Discovery Studio 2017 was used to perform the calculations and to generate the graphical results (DassaultSystèmes Vélizy-Villacoublay France).

**Neurounina-1** and compounds **1–19** were built using the Small Molecule tool, and atomic potentials and charges were assigned using the CFF forcefield.^59^

The conformational space of the generated structures was sampled using the stochastic conformational search algorithm BEST for the random generation of a maximum of 1000 conformations (Search Small Molecule Conformations; Discovery Studio 2017). An energy threshold value of 10^6^ kcal/mol was applied as selection criteria in order to ensure a wide variance of the resulting structures. The generalized Born implicit solvent model with a solvent dielectric constant value of 80 was used to mimic an aqueous environment.^60^ The generated conformations were then subjected to energy minimization until the maximum RMS derivative was less than 0.01 kcal/Å using conjugate gradient as the minimization algorithm.^61^ The resulting conformers were ranked by their potential energy values [that is, energy difference from the global minimum (Δ*E*_GM_)] and the low-energy conformers (Δ*E*_GM_ ≤ 5 kcal/mol) were analyzed.

First, the structures were grouped according to the flip of the benzodiazepinone ring system, that is, positive or negative value of the torsional angle related to the rotation around the CH_2_–C=O bond and having at the 1.4 positions the nitrogen atoms (namely, τ_flip_ = ±65°). This generated two sets of specular conformers having the same energy values and the opposite sign of their torsion angles (for chiral compounds, they corresponded to the conformers of the two configurational enantiomers).

Then, the two resulting sets were classified into families according to the values of the first two dihedral angles of the spacer (namely, τ_1_ and τ_2_). Family classification corresponded to the following criteria: I (−30° < τ_1_ < −90°), II (30° < τ_1_ < 90°), III (−90° < τ_1_ < −150°), IV (90° < τ_1_ < 150°), a (30° < τ_2_ < 90°), b (−30° < τ_2_ < −90°), c (τ_2_ ∼ ±180°), d (τ_2_ ∼ 0°), e (−90° < τ_2_ < −150°), and f (90° < τ_2_ < 150°).

The ranges of torsional angle values refer to the conformers with τ_flip_ = −65°, those applied to the specular conformers (τ_flip_ = 65°) present the same absolute values with the opposite sign. Resulting conformational families of each compound were ranked according to their Δ*E*_GM_ values and analyzed by fitting the following: (i) the centroid of the benzo-fused ring, (ii) the centroid of the pendant phenyl ring, (iii) the carbonyl oxygen atom of the benzodiazepinone ring, and (iv) the first carbon atom of the spacer group. Pharmacophore points and shape features were generated by using the Pharmacophores module of Discovery Studio 2017. Molecular shapes were combined by using Boolean operators.

#### Bioinformatic and Structural Analysis

The sequences of sodium/calcium exchanger 1 of *C. lupus familiaris* (entry P23685), sodium/calcium exchanger 1 of *Homo sapiens* (entry P32418), sodium/calcium exchanger 2 of *Rattus* (entry P48768), sodium/calcium exchanger 2 of *H. sapiens* (entry Q9UPR5), sodium/calcium exchanger 3 of *Rattus* (entry P70549), and sodium/calcium exchanger 3 of *H. sapiens* (entry P57103) were downloaded from the UniProtKB/Swiss-Prot Data Bank (http://www.uniprot.org).

NCX sequence alignments were performed using the PROMALS3D server (http://prodata.swmed.edu/promals3d/promals3d.php)^62^ using the following parameters: identity threshold: 0.6; weight for sequence-based constraints: 1; weight for amino acid scores: 0.8; weight for predicted secondary structure scores: 0.2; PSI-BLAST iteration number: 3; PSI-BLAST e-value inclusion threshold: 0.001; identity cutoff below which distant homologues are removed: 0.25; maximum number of homologues kept for each blast run: 300; PSI-BLAST e-value cutoff against structural database: 0.001; and identity cutoff below which 3D structure templates are not used: 0.2.

Secondary structure predictions were performed using the PredictProtein server (www.predictprotein.org).^63^

The presence of linear functional motifs, such as heptad repeats and LxxLL-like protein recognition motifs were predicted using the following consensus sequences: (i) heptad repeat: [VLIFYWM]xx[VLIFYWM]xxx[VLIFYWM]xx[VLIFYWM]xxx; (ii) LxxLL-like motifs: [VLIFYWM]xx[VLIFYWM][VLIFYWM], [VLIFYWM][VLIFYWM]xx[VLIFYWM], [VLIFYWM] xx[VLIFYWM] [VLIFYWM]xx[VLIFYWM] (Predict Sequence Properties protocol, BIOVIA Discovery Studio software, Dassault Systèmes).

Experimentally determined structures of the following: (i) human bromodomain-containing protein 4 (BRD4) in complex with I-BET (PDB ID: 3P5O)^64^ and with alprazolam (PDB ID: 3U5J);^65^ (ii) pentameric ligand-gated ion channel from *E. chrysanthemi* (ELIC) in complex with flurazepam and bromoflurazepam (PDB IDs: 2YOE and 4A98);^66^ and (iii) GABA_A_ receptor in complex with diazepam and GABA (PDB: 6HUP)^67^ were downloaded from the Protein Data Bank (PDB, http://www.rcsb.org/pdb/) and analyzed using the Macromolecules tool of Discovery Studio 2020 (DassaultSystèmes BIOVIA, San Diego). The sequences of the NCX1 α1- and α2-repeats reported to be involved in **Neurounina-1** binding (i.e., aa141–180 and aa842–879, respectively^25^) were aligned with the sequences of the benzodiazepine binding sites (i.e., residues with at least one atom within 5 Å from any ligand atom) of the above-listed PDB complexes.

Sequence alignments were performed using the BIOVIA Discovery Studio 2020 software (Dassault Systèmes) using the following parameters: Pairwise Alignment Algorithm: Slow; Scoring Matrix: BLOSUM 30; Gap Open Penalty: 1; Gap Extension Penalty: 0.01; Gap Penalty 5.0; and Window Size: 5. The lowest energy conformer of **Neurounina-1** was superimposed on BET-I (3U5J) and flurazepam by fitting the heavy atoms of the common benzodiazepine skeleton.

The X-ray structures of the sodium/calcium exchanger from *M. jannaschii* (PDB IDs: 3V5U, 5HYA, 5HXC, 5HXE, 5HXR, 5HXS, 5HXH, 5HWX, 5HWY, 5JDF, 5JDG, 5JDH, 5JDL, 5JDM, 5JDN, and 5JDQ)^34,35^ were downloaded from the PDB and analyzed using the Macromolecules tool of Discovery Studio 2020 (DassaultSystèmes BIOVIA, San Diego). In order to obtain molecular models of interaction with *M. jannaschii* NCX, first, the heavy atoms of the proline residue P82 of BRD4 (3U5J) were superimposed on the heavy atoms of the proline residue P212 of Mj_NCX X-ray structures (PDB IDs: 5HWY, 5JDF, and 5HYA), corresponding to P848 in human NCX, and then, the calculated lowest energy conformation of **Neurounina-1**, **4**, and **14** on the X-ray structure of alprazolam by the common phenyl-benzodiazepine skeleton.

The amino acids not conserved at the level of 10 TM helices (TM1–TM10) among the three different isoforms of NCX (i.e., NCX1 Canis, NCX2 Rat, and NCX3 Rat) were displayed on the molecular interaction model between **Neurounina-1** and *M. jannaschii* NCX (PDB ID: 5HWY) by comparing the alignments of the NCX sequences (isoforms and species) with that published by Liao et al. between *M. jannaschii* NCX and human NCX1.^31^

The topology model of the IF sodium/calcium exchanger from *M. jannaschii* (PDB ID: 5HWY) was generated by superimposing the α carbon atoms of the terminal residues of helices 2B, 2C, and 3A on those of helices 7B, 7C, and 8A, as described by Liao et al.^34^

## Supporting Information Available

Calculated prevalent ionic forms, clog *D* and clog *P* of **Neurounina-1**, compounds **1–19**, ethanol, citalopram, nifedipine, and Bay K 8644; Δ*E*_GM_ values (kcal/mol) and torsional angle values (degrees) of conformational families; comparison of the global minimum conformers (Δ*E*_GM_ = 0 kcal/mol) of **1**; α1 and α2 repeats as putative **Neurounina-1** binding sites; X-ray structures of the BRD4/I-BET and ELIC/flurazepam complexes; molecular interaction model between compound **4** and NCX_Mj; molecular interaction model between compound **14** and NCX_Mj; topology model of the OF and IF NCX_Mj; predicted LxxLL-like motifs and heptad repeats on TM1, TM2, TM6, and TM7 of *M. jannaschii* NCX and *C. lupus familiaris* NCX1; mapping of the residues not conserved among the three different isoforms of NCX (*C. lupus familiaris* NCX1, *Rattus* NCX2 and *Rattus* NCX3) on the molecular interaction model between **Neurounina-1** and NCX_Mj; multiple sequence alignment of the TM domains of *C. lupus familiaris* NCX1, *Rattus* NCX2, and *Rattus* NCX3; and multiple sequence alignment of the TM domains of human NCX1, NCX2, and NCX3 (PDF)
Molecular formula strings (CSV)
PDB coordinates of Model_Cmp4_5HXR (PDB)
PDB coordinates of Model_Cmp14_5JDF (PDB)
PDB coordinates of Model_Neu1_5HWY (PDB)

## Supplementary Material

jm1c01212_si_002.csv

jm1c01212_si_003.pdb

jm1c01212_si_004.pdb

jm1c01212_si_005.pdb

## Abbreviations

BHK: baby hamster kidney
BRD4: human bromodomain-containing protein 4
CBD1: calcium binding site 1
CBD 2: calcium binding site 2
ELIC: Erwinia chrysanthemi ligand-gated ion channel
FURA 2-AM: Fura-2-acetoxymethyl ester
HOBt: 1-hydroxybenzotriazole
MS: multiple sclerosis
NCX: Na^+^/Ca^2+^ exchanger
NCX_Mj: Na^+^/Ca^2+^ exchanger of *Archaebacterium**Methanococcus jannaschii*
NMDG: *N*-methyl-d-glucamine
OGD: oxygen and glucose deprivation
PC: ischemic preconditioning
RX: reoxygenation
SMA: spinal muscular atrophy
TBTU: 2-(1*H*-benzotriazole-1-yl)-1,1,3,3-tetramethylaminium tetrafluoroborate
