# Supplementary material for: New Insights into the Structure–Activity Relationship and Neuroprotective Profile of Benzodiazepinone Derivatives of Neurounina-1 as Modulators of the Na+/Ca2+ Exchanger Isoforms
Source: J Med Chem. 2021 Nov 30;64(24):17901–19. doi: 10.1021/acs.jmedchem.1c01212 (PMC8713167; doi:10.1021/acs.jmedchem.1c01212)
Supplement: Supplementary file 1 — jm1c01212_si_001.pdf [file jm1c01212_si_001.pdf]

Supporting Information

New insights into Structure-Activity-Relationship (SAR) and neuroprotective profile of benzodiazepinone derivatives of Neurounina-1 as modulators of the Na+/Ca2+ Exchanger isoforms

Elisa Magli,†° Caterina Fattorusso,†° Marco Persico,† Angela Corvino,† Gianluca Esposito,† Ferdinando Fiorino,† Paolo Luciano,† Elisa Perissutti,† Vincenzo Santagada,† Beatrice Severino,† Valentina Tedeschi,‡ Anna Pannaccione,‡ Giuseppe Pignataro,‡ Giuseppe Caliendo,† Lucio Annunziato,§ Agnese Secondo,‡ Francesco Frecentese†*

° These two authors equally contributed to this article.

† *Department of Pharmacy, University of Naples «Federico II», Via D. Montesano 49, 80131, Naples, Italy*

‡ *Department of Neuroscience, Division of Pharmacology, University of Naples «Federico II», via Pansini 5, 80131, Naples, Italy*

§ *IRCCS SD, via F.Crispi 8, 80121, Naples, Italy*

* To whom correspondence should be addressed.

E-mail: frecente@unina.it

Content:

Calculated prevalent ionic forms, clogD and clogP of **Neurounina-1**, compounds **1-19,** ethanol**,** citalopram, nifedipine, and Bay K 8644 (Table S1)……...S2

ΔE_{GM} values (kcal/mol) and torsional angle values (degrees) of conformational families of **Neurounina-1** and compounds **1-19** (Table S2-S21)……...S3-S22

The effect of compounds **1-19** on NCX1 and NCX3 reverse mode activity measured in BHK-NCX1 and BHK-NCX3 loaded by Fura-2 AM (Figure S1) ………………………………………….………. S23

Concentration dependent neuroprotective profile in cortical neurons of compound **1** (Figure S2)……………………………………………………………………………………………..S24

Comparison of the global minimum conformers (ΔE_{GM} = 0 kcal/mol) of **1** (Figure S3)………………..…………………...………………………S25

The α1 and α2 repeat regions identified to be involved in **Neurounina-1** binding to NCX1 (Figure S4)…………………………...……………………………………………………………….….S26

X-ray structures of the BRD4/I-BET and ELIC/flurazepam complexes (Figure S5)……………………………………………………………………………………………..…S27

Molecular interaction model between compound **4** and NCX_mj resulting from our bioinformatics and structural analysis (Figure S6)………………………………………………………………………...S28

Molecular interaction model between compound **14** and NCX_mj resulting from our bioinformatics and structural analysis (Figure S7).……………………………………………………………….…….S29

Topology model of the outward- and inward-facing NCX_mj (Figure S8)……………………………..S30

Predicted protein-protein interaction motifs on TM1, TM2, TM6, and TM7 of NCX_mj and *Canis lupus familiaris* NCX1 (Figure S9)…………………………………...S31

Residues not conserved among the NCX isoforms used in the experimental studies (*Canis lupus familiaris* NCX1, *Rattus* NCX2 and *Rattus* NCX3) mapped on the molecular interaction model between **Neurounina-1** and NCX_Mj (Figure S10)……………………………………………………………...S32

Multiple sequences alignment (excluding the f-loop) of *Canis lupus familiaris* NCX1, *Rattus* NCX2 and *Rattus* NCX3 (Figure S11)……………………………………………………..…..…………S33

Multiple sequences alignment (excluding the f-loop) of *Human* NCX1, NCX2 and NCX3 (Figure S12)…………………………………………...………………………........……….S34

Table S1. Prevalent ionic forms, clogP and clogD.[a]

Compound	Prevalent ionic form (%)		clogD		clogP
	pH 7.4	pH 7.2	pH 7.4	pH 7.2	-
**Neurounina-1**	P (72%)	P (80%)	1.75	1.59	2.43±1.05
**1**	P (72%)	P (80%)	1.93	1.77	2.59±1.08
**2**	P (83%)	P (89%)	2.91	2.74	3.77±0.95
**3**	P (93%)	P (95%)	1.76	1.57	2.99±1.05
**4**	P (96%)	P (98%)	0.96	0.77	2.43±1.04
**5**	P (96%)	P (98%)	1.49	1.30	2.99±1.04
**6**	P (98%)	P (99%)	1.70	1.51	3.48±1.04
**7**	P (97%)	P (98%)	1.95	1.76	3.48±1.04
**8**	N (80%)	N (72%)	1.37	1.33	1.46±1.03
**9**	N (56%)	N (67%)	1.16	1.03	1.54±1.02
**10**	N (72%)	N (61%)	3.37	3.32	3.47±1.08
**11**	N (72%)	N (61%)	4.43	4.33	4.70±1.08
**12**	N (100%)	N (100%)	1.83	1.83	1.83±1.09
**13**	N (100%)	N (100%)	2.40	2.40	2.40±1.09
**14**	N (100%)	N (100%)	2.89	2.89	2.89±1.09
**15**	N (100%)	N (100%)	2.89	2.89	2.89±1.09
**16**	N (100%)	N (100%)	1.33	1.33	1.33±1.03
**17**	N (100%)	N (100%)	1.33	1.33	1.33±1.12
**18**	N (100%)	N (100%)	2.60	2.60	2.60±1.13
**19**	N (100%)	N (100%)	3.83	3.83	3.83±1.13
**Ethanol**	N (100%)	N (100%)	-0.19	-0.19	-0.19±0.18
**Citalopram**	P (99%)	P (100%)	0.39	0.21	2.51±0.74
**Nifedipine**	N (100%)	N (100%)	1.58	1.58	1.58±0.42
**Bay K 8644**	N (100%)	N (100%)	2.39	2.39	2.39±0.42

[a]ACD/Percepta, Advanced Chemistry Development, Inc., Toronto, ON, Canada, 2017, http://www.acdlabs.com. P: protonated form; N: neutral form.

Table S2. ΔE_{GM} values (kcal/mol) and torsional angle values (degrees) of conformational families of **Neurounina-1** considering MM conformers within 5 kcal/mol from the global minimum characterized by negative value of torsional angle $\tau_{flip.}$

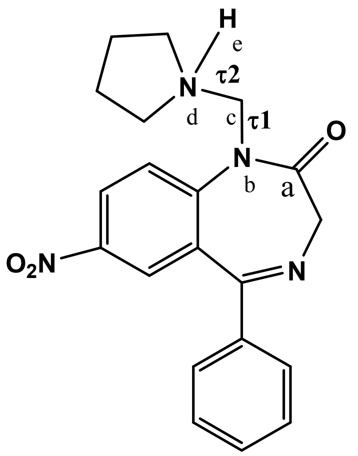

Family	ΔE_{GM}[a] (kcal/mol)	Torsional Angles (°)[b,c]	
		τ1[d]	τ2[e]
**Ia**	0.00	-57.03	55.48
**IIb**	1.43	57.92	-57.10
**IIIb**	1.91	-128.67	-61.65
**IIa**	3.03	85.89	43.07
**IIIc**	3.49	-103.21	173.63
**IVa**	4.52	97.24	45.90

[a]The values reported refer to the lowest and the highest energy conformers of the family. [b]The conformational enantiomer present the same absolute values of torsional angles with the opposite sign. [c]The values reported refer to the lowest energy conformer of the family. [d] τ1: a, b, c, and d atoms. [e] τ2: b, c, d, and e atoms.

Table S3. ΔE_{GM} values (kcal/mol) and torsional angle values (degrees) of conformational families of compound **1** considering MM conformers within 5 kcal/mol from the global minimum characterized by negative value of torsional angle τ_{flip}.

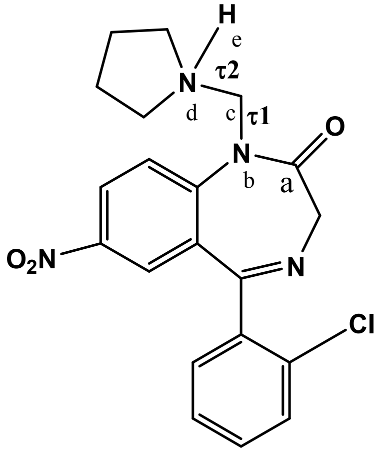

Family	ΔE_{GM}[a] (kcal/mol)	Torsional Angles (°)[b,c]	
		τ1[d]	τ2[e]
**Ia**	0.00-1.34	-55.50	57.67
**IIb**	1.60-2.72	57.62	-58.69
**IIIb**	2.42-3.44	-128.72	-51.58
**IIa**	3.18-4.51	85.59	41.10
**IIIa**	3.34	-111.56	40.90

[a]The values reported refer to the lowest and the highest energy conformers of the family. [b]The conformational enantiomer present the same absolute values of torsional angles with the opposite sign. [c]The values reported refer to the lowest energy conformer of the family. [d] τ1: a, b, c, and d atoms. [e] τ2: b, c, d, and e atoms.

Table S4. ΔE_{GM} values (kcal/mol) and torsional angle values (degrees) of conformational families of compound **2** considering MM conformers within 5 kcal/mol from the global minimum characterized by negative value of torsional angle τ_{flip}.

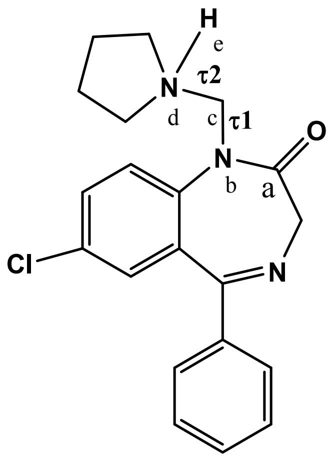

Family	ΔE_{GM}[a] (kcal/mol)	Torsional Angles (°)[b,c]	
		τ1[d]	τ2[e]
**Ia**	0.00	-57.69	59.73
**IIb**	1.48	57.93	-57.92
**IIIb**	1.92	-129.37	-56.87
**IVa**	3.07	97.66	46.26
**IIIc**	3.34	-92.80	173.49

[a]The values reported refer to the lowest and the highest energy conformers of the family. [b]The conformational enantiomer present the same absolute values of torsional angles with the opposite sign. [c]The values reported refer to the lowest energy conformer of the family. [d] τ1: a, b, c, and d atoms. [e] τ2: b, c, d, and e atoms.

Table S5. ΔE_{GM} values (kcal/mol) and torsional angle values (degrees) of conformational families of compound **3** considering MM conformers within 5 kcal/mol from the global minimum characterized by negative value of torsional angle τ_{flip}.

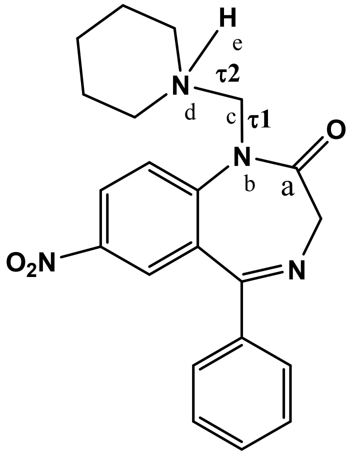

Family	ΔE_{GM}[a] (kcal/mol)	Torsional Angles (°)[b,c]	
		τ1[d]	τ2[e]
**Ia**	0.00	-63.59	55.87
**IIb**	0.77	59.61	-54.85
**IIIb**	1.81	-128.44	-57.62
**IVa**	2.98	90.47	53.51

[a]The values reported refer to the lowest and the highest energy conformers of the family. [b]The conformational enantiomer present the same absolute values of torsional angles with the opposite sign. [c]The values reported refer to the lowest energy conformer of the family. [d] τ1: a, b, c, and d atoms. [e] τ2: b, c, d, and e atoms.

Table S6. ΔE_{GM} values (kcal/mol) and torsional angle values (degrees) of conformational families of compound **4** considering MM conformers within 5 kcal/mol from the global minimum characterized by negative value of torsional angle τ_{flip}.

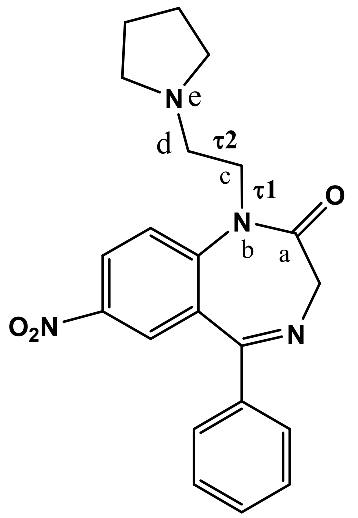

**Family**	**ΔE_{GM}[a] (kcal/mol)**	**Torsional Angles (°)[b,c]**	
		τ1[d]	τ2[e]
**IIc**	0.00-0.92	74.06	-178.96
**IIb**	0.73-1.58	75.65	-81.13
**Ic**	1.02-2.07	-76.10	179.01
**Ia**	1.76-1.79	-85.73	60.63
**Ib**	2.02-3.34	-60.94	-64.41
**IIa**	2.73-4.02	65.19	56.05
**IIIa**	3.18-4.06	-111.49	65.00

[a]The values reported refer to the lowest and the highest energy conformers of the family. [b]The conformational enantiomer present the same absolute values of torsional angles with the opposite sign. [c]The values reported refer to the lowest energy conformer of the family. [d] τ1: a, b, c, and d atoms. [e] τ2: b, c, d, and e atoms.

Table S7. ΔE_{GM} values (kcal/mol) and torsional angle values (degrees) of conformational families of compound **5** considering MM conformers within 5 kcal/mol from the global minimum characterized by negative value of torsional angle τ_{flip}.

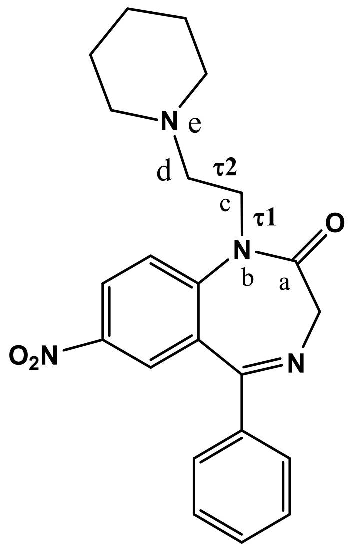

Family	ΔE_{GM}[a] (kcal/mol)	Torsional Angles (°)[b,c]	
		τ1[d]	τ2[e]
**IIc**	0.00-0.82	74.22	-175.05
**IIb**	0.31-1.40	74.82	-79.67
**Ic**	1.00-1.78	-75.38	175.56
**Ib**	1.70-3.19	-61.65	-65.89
**Ia**	2.18-2.54	-89.68	61.91
**IIIa**	2.63-4.01	-101.57	65.64
**IIa**	2.80-4.77	63.47	60.65

[a]The values reported refer to the lowest and the highest energy conformers of the family. [b]The conformational enantiomer present the same absolute values of torsional angles with the opposite sign. [c]The values reported refer to the lowest energy conformer of the family. [d] τ1: a, b, c, and d atoms. [e] τ2: b, c, d, and e atoms.

Table S8. ΔE_{GM} values (kcal/mol) and torsional angle values (degrees) of conformational families of the two enantiomers of compound **6** considering MM conformers within 5 kcal/mol from the global minimum characterized by negative value of torsional angle τ_{flip}.

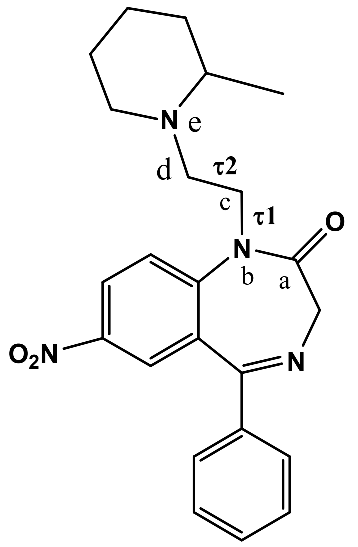

Family	ΔE_{GM}[a] (kcal/mol)	Torsional Angles (°)[b,c]	
		τ1[d]	τ2[e]
**IIc**	0.00-3.02	74.42	-170.61
**Ic**	0.72-4.22	-73.73	179.82
**IIb**	1.09	76.66	-76.68
**Ib**	1.45-4.02	-62.20	-67.38
**Ia**	1.88-2.89	-86.07	68.45
**IIIa**	3.12-3.83	-107.20	74.58
**IIa**	3.98	63.22	62.28
**IIIb**	4.53	-92.30	-82.62

[a]The values reported refer to the lowest and the highest energy conformers of the family. [b]The configurational enantiomer present the same absolute values of torsional angles with the opposite sign. [c]The values reported refer to the lowest energy conformer of the family. [d] τ1: a, b, c, and d atoms. [e]τ2: b, c, d, and e atoms.

Table S9. ΔE_{GM} values (kcal/mol) and torsional angle values (degrees) of conformational families of the two enantiomers of compound **7** considering MM conformers within 5 kcal/mol from the global minimum characterized by negative value of torsional angle τ_{flip}.

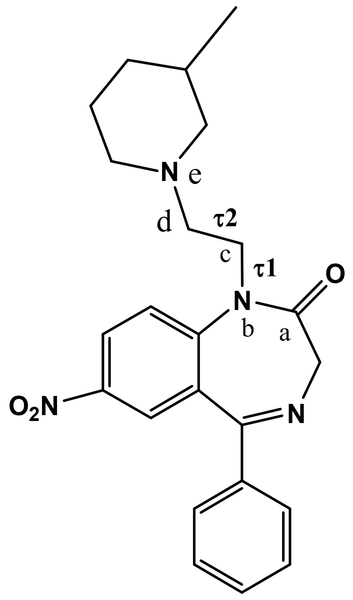

Family	ΔE_{GM}[a] (kcal/mol)	Torsional Angles (°)[b,c]	
		τ1[d]	τ2[e]
**IIc**	0.00-3.00	74.18	-174.93
**IIb**	0.54-3.44	77.33	-73.49
**Ic**	1.08-3.73	-76.86	175.57
**Ib**	1.33-4.82	-58.11	-48.84
**Ia**	2.21	-89.95	62.76
**IIIa**	2.43-4.51	-109.13	71.44
**IIa**	2.69-3.68	63.28	60.24

[a]The values reported refer to the lowest and the highest energy conformers of the family. [b]The configurational enantiomer present the same absolute values oftorsional angles with the opposite sign. [c]The values reported refer to the lowest energy conformer of the family. [d]τ1: a, b, c, and d atoms. [e]τ2: b, c, d, and e atoms.

Table S10. ΔE_{GM} values (kcal/mol) and torsional angle values (degrees) of conformational families of compound **8** considering MM conformers within 5 kcal/mol from the global minimum characterized by negative value of torsional angle τ_{flip}.

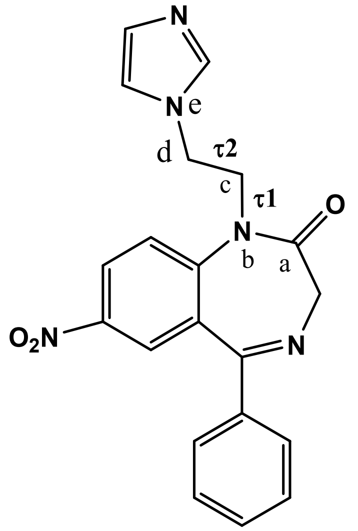

**Family**	**ΔE_{GM}[a] (kcal/mol)**	**Torsional Angles (°)[b,c]**	
		τ1[d]	τ2[e]
**Ib**	0.00-0.11	-67.17	-58.82
**IIa**	0.29-0.38	68.83	61.75
**IIc**	1.30-1.31	74.40	177.57
**IIIa**	1.50	-90.12	63.85
**Ia**	1.63	-88.84	63.50
**IIb**	1.51-1.58	82.79	-63.27
**Ic**	2.15-2.17	-75.04	-179.96

[a]The values reported refer to the lowest and the highest energy conformers of the family. [b]The conformational enantiomer present the same absolute values of torsional angles with the opposite sign. [c]The values reported refer to the lowest energy conformer of the family. [d]τ1: a, b, c, and d atoms. [e]τ2: b, c, d, and e atoms.

Table S11. ΔE_{GM} values (kcal/mol) and torsional angle values (degrees) of conformational families of compound **9** considering MM conformers within 5 kcal/mol from the global minimum characterized by negative value of torsional angle τ_{flip}.

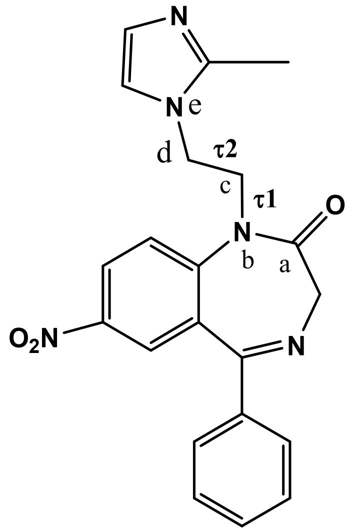

Family	ΔE_{GM}[a] (kcal/mol)	Torsional Angles (°)[b,c]	
		τ1[d]	τ2[e]
**Ib**	0.00-0.01	-67.53	-55.31
**IIa**	0.44-0.66	69.99	67.78
**IIc**	1.50-1.73	74.63	-179.48
**IIb**	1.56-1.89	81.80	-63.05
**Ia**	2.14	-84.60	69.39
**IIIa**	3.32	-128.28	50.97
**Ic**	2.38-2.60	-75.67	-177.90
**IIIb**	4.28	-113.88	-74.11

[a]The values reported refer to the lowest and the highest energy conformers of the family. [b]The conformational enantiomer present the same absolute values of torsional angles with the opposite sign. [c]The values reported refer to the lowest energy conformer of the family. [d]τ1: a, b, c, and d atoms. [e]τ2: b, c, d, and e atoms.

Table S12. ΔE_{GM} values (kcal/mol) and torsional angle values (degrees) of conformational families of compound **10** considering MM conformers within 5 kcal/mol from the global minimum characterized by negative value of torsional angle τ_{flip}.

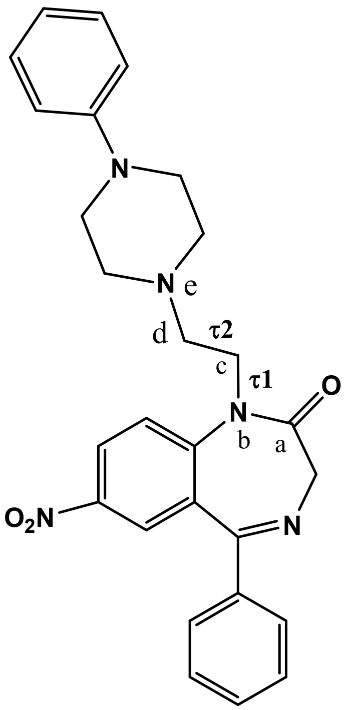

Family	ΔE_{GM}[a] (kcal/mol)	Torsional Angles (°)[b,c]	
		τ1[d]	τ2[e]
**Ib**	0.00-4.77	-80.16	-61.39
**IIIa**	0.93-4.92	-116.94	52.18
**IIa**	1.72-3.78	68.89	56.32
**IIc**	2.04-2.06	74.65	169.96
**IIIb**	2.97-4.17	-103.13	-68.04
**IIb**	3.07-4.94	82.46	-68.99

[a]The values reported refer to the lowest and the highest energy conformers of the family. [b]The conformational enantiomer present the same absolute values of torsional angles with the opposite sign. [c]The values reported refer to the lowest energy conformer of the family. [d]τ1: a, b, c, and d atoms. [e]τ2: b, c, d, and e atoms.

Table S13. ΔE_{GM} values (kcal/mol) and torsional angle values (degrees) of conformational families of compound **11** considering MM conformers within 5 kcal/mol from the global minimum characterized by negative value of torsional angle τ_{flip}.

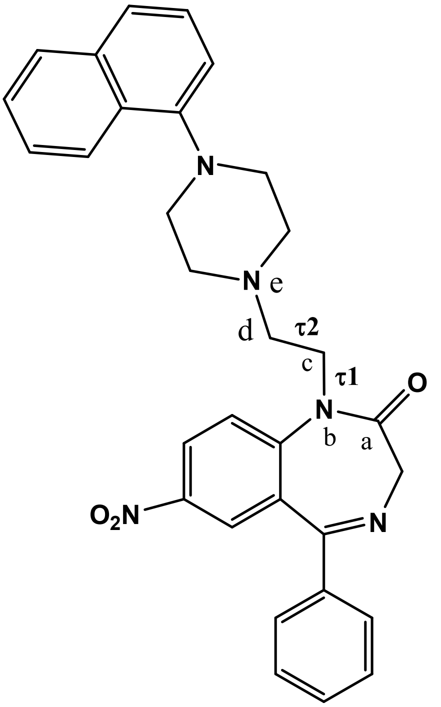

Family	ΔE_{GM}[a] (kcal/mol)	Torsional Angles (°)[b,c]	
		τ1[d]	τ2[e]
**Ib**	0.00-4.56	-75.40	-63.18
**IIIa**	0.60-4.93	-110.00	52.61
**IIIb**	0.68-4.87	-91.62	-62.75
**IIa**	2.18-2.26	68.68	57.80

[a]The values reported refer to the lowest and the highest energy conformers of the family. [b]The conformational enantiomer present the same absolute values of torsional angles with the opposite sign. [c]The values reported refer to the lowest energy conformer of the family. [d]τ1: a, b, c, and d atoms. [e]τ2: b, c, d, and e atoms.

Table S14. ΔE_{GM} values (kcal/mol) and torsional angle values (degrees) of conformational families of compound **12** considering MM conformers within 5 kcal/mol from the global minimum characterized by negative value of torsional angle τ_{flip}.

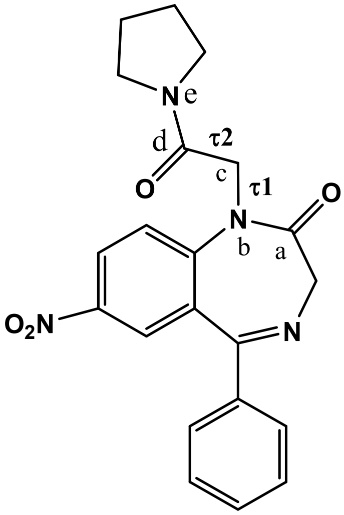

Family	ΔE_{GM}[a] (kcal/mol)	Torsional Angles (°)[b,c]	
		τ1[d]	τ2[e]
**IIIc**	0.00-0.31	-132.61	-164.85
**IIc**	0.50-0.60	86.83	171.75
**IIIa**	2.64-2.97	-138.11	65.53

[a]The values reported refer to the lowest and the highest energy conformers of the family. [b]The conformational enantiomer present the same absolute values of torsional angles with the opposite sign. [c]The values reported refer to the lowest energy conformer of the family. [d]τ1: a, b, c, and d atoms. [e]τ2: b, c, d, and e atoms.

Table S15. ΔE_{GM} values (kcal/mol) and torsional angle values (degrees) of conformational families of compound **13** considering MM conformers within 5 kcal/mol from the global minimum characterized by negative value of torsional angle τ_{flip}.

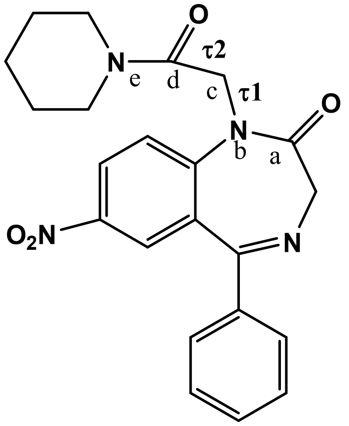

Family	ΔE_{GM}[a] (kcal/mol)	Torsional Angles (°)[b,c]	
		τ1[d]	τ2[e]
**IIIc**	0.00-0.35	-129.60	-167.60
**IIc**	0.42-0.52	86.17	175.34
**IIIa**	2.82-3.02	-142.12	61.26

[a]The values reported refer to the lowest and the highest energy conformers of the family. [b]The conformational enantiomer present the same absolute values of torsional angles with the opposite sign. [c]The values reported refer to the lowest energy conformer of the family. [d]τ1: a, b, c, and d atoms. [e]τ2: b, c, d, and e atoms.

Table S16. ΔE_{GM} values (kcal/mol) and torsional angle values (degrees) of conformational families of the two enantiomers of compound **14** considering MM conformers within 5 kcal/mol from the global minimum characterized by negative value of torsional angle τ_{flip}.

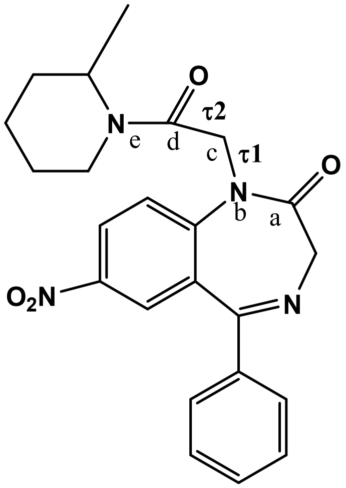

Family	ΔE_{GM}[a] (kcal/mol)	Torsional Angles (°)[b,c]	
		τ1[d]	τ2[e]
**IIIc**	0.00-0.63	-129.71	-168.83
**IIc**	0.28-4.81	84.92	177.24
**IIIa**	4.99	-143.66	49.12

[a]The values reported refer to the lowest and the highest energy conformers of the family. [b]The configurational enantiomer present the same absolute values of torsional angles with the opposite sign. [c]The values reported refer to the lowest energy conformer of the family. [d]τ1: a, b, c, and d atoms. [e]τ2: b, c, d, and e atoms.

Table S17. ΔE_{GM} values (kcal/mol) and torsional angle values (degrees) of conformational families of the two enantiomers of compound **15** considering MM conformers within 5 kcal/mol from the global minimum characterized by negative value of torsional angle τ_{flip}.

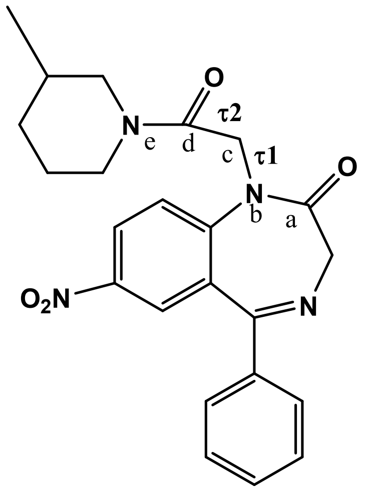

Family	ΔE_{GM}[a] (kcal/mol)	Torsional Angles (°)[b,c]	
		τ1[d]	τ2[e]
**IIIc**	0.00-0.56	-128.24	-165.77
**IIc**	0.42-0.73	86.28	176.86
**IIIa**	2.75	-133.39	87.47

[a]The values reported refer to the lowest and the highest energy conformers of the family. [b]The configurational enantiomerpresent the same absolute values oftorsional angles with the opposite sign. [c]The values reported refer to the lowest energy conformer of the family. [d]τ1: a, b, c, and d atoms. [e]τ2: b, c, d, and e atoms.

Table S18. ΔE_{GM} values (kcal/mol) and torsional angle values (degrees) of conformational families of compound **16** considering MM conformers within 5 kcal/mol from the global minimum characterized by negative value of torsional angle τ_{flip}.

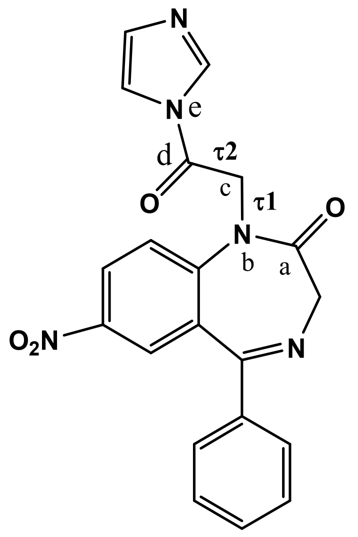

Family	ΔE_{GM}[a] (kcal/mol)	Torsional Angles (°)[b,c]	
		τ1[d]	τ2[e]
**IIIa**	0.00-0.97	-142.99	59.18
**IIIe**	1.26-2.06	-130.55	-124.51
**IId**	2.61-3.45	86.49	-3.39
**Ib**	3.56-4.28	-53.62	-45.81
**IVf**	3.60-4.54	92.70	138.72

[a]The values reported refer to the lowest and the highest energy conformers of the family. [b]The conformational enantiomer present the same absolute values of torsional angles with the opposite sign. [c]The values reported refer to the lowest energy conformer of the family. [d]τ1: a, b, c, and d atoms. [e]τ2: b, c, d, and e atoms.

Table S19. ΔE_{GM} values (kcal/mol) and torsional angle values (degrees) of conformational families of compound **17** considering MM conformers within 5 kcal/mol from the global minimum characterized by negative value of torsional angle τ_{flip}.

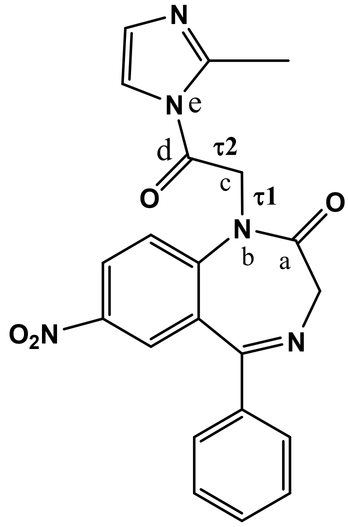

Family	ΔE_{GM}[a] (kcal/mol)	Torsional Angles (°)[b,c]	
		τ1[d]	τ2[e]
**IIIa**	0.00	-143.27	59.74
**IIIe**	1.30	-131.11	-124.73
**Ib**	3.92	-53.46	-49.39
**IVf**	4.03	93.54	137.93

[a]The values reported refer to the lowest and the highest energy conformers of the family. [b]The conformational enantiomer present the same absolute values of torsional angles with the opposite sign. [c]The values reported refer to the lowest energy conformer of the family. [d]τ1: a, b, c, and d atoms. [e]τ2: b, c, d, and e atoms.

Table S20. ΔE_{GM} values (kcal/mol) and torsional angle values (degrees) of conformational families of compound **18** considering MM conformers within 5 kcal/mol from the global minimum characterized by negative value of torsional angle τ_{flip}.

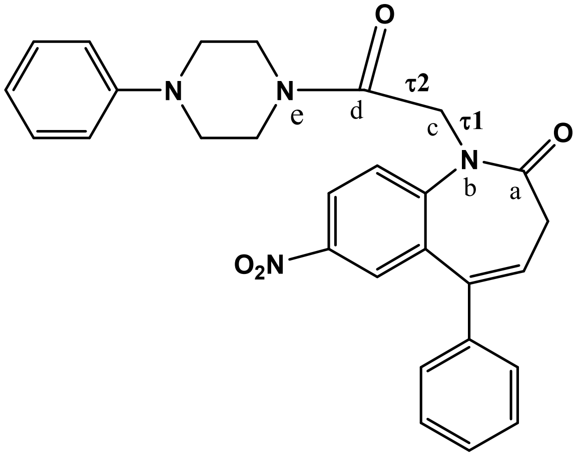

Family	ΔE_{GM}[a] (kcal/mol)	Torsional Angles (°)[b,c]	
		τ1[d]	τ2[e]
**IIIc**	0.00-3.51	-126.75	-171.60
**IIc**	0.34-1.69	84.97	175.28
**IIIa**	2.47-4.40	-125.97	91.78

[a]The values reported refer to the lowest and the highest energy conformers of the family. [b]The conformational enantiomer present the same absolute values of torsional angles with the opposite sign. [c]The values reported refer to the lowest energy conformer of the family. [d]τ1: a, b, c, and d atoms. [e]τ2: b, c, d, and e atoms.

Table S21. ΔE_{GM} values (kcal/mol) and torsional angle values (degrees) of conformational families of compound **19** considering MM conformers within 5 kcal/mol from the global minimum characterized by negative value of torsional angle τ_{flip}.

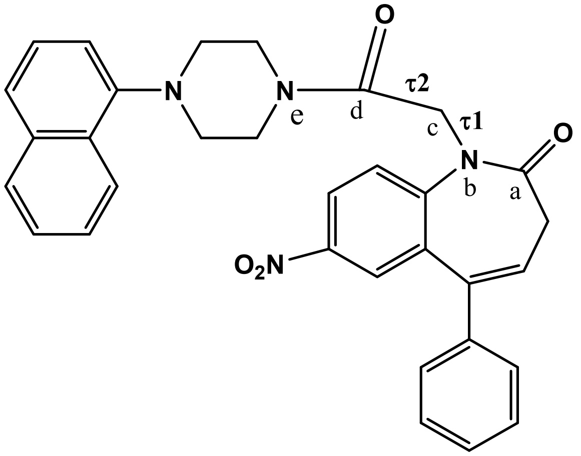

**Family**	**ΔE_{GM}[a] (kcal/mol)**	**Torsional Angles (°)[b,c]**	
		τ1[d]	τ2[e]
**IIIc**	0.00-4.97	-110.80	-145.47
**IIc**	0.52-1.88	84.84	175.69
**IIIa**	1.89-4.43	-124.72	93.36

[a]The values reported refer to the lowest and the highest energy conformers of the family. [b]The conformational enantiomer present the same absolute values of torsional angles with the opposite sign. [c]The values reported refer to the lowest energy conformer of the family. [d]τ1: a, b, c, and d atoms. [e]τ2: b, c, d, and e atoms.

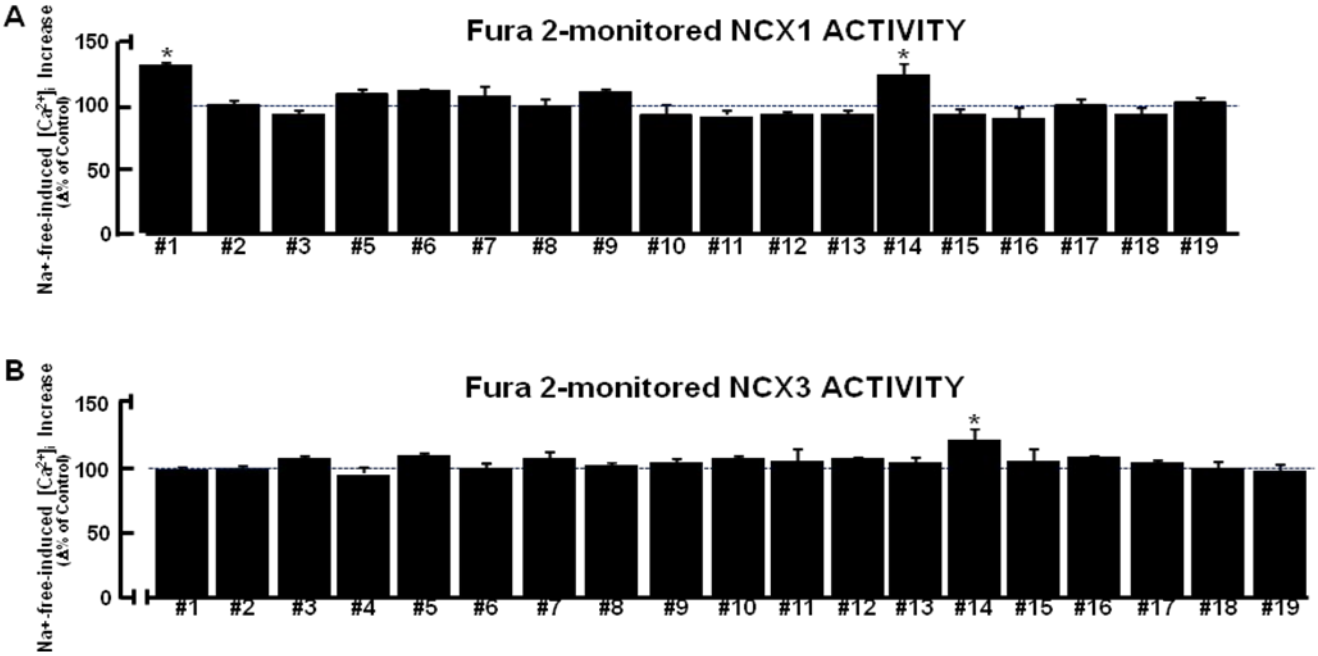

Figure S1. The effect of the compounds **1-19** on NCX1 and NCX3 reverse mode activity measured in BHK-NCX1 and BHK-NCX3 loaded by Fura-2 AM.

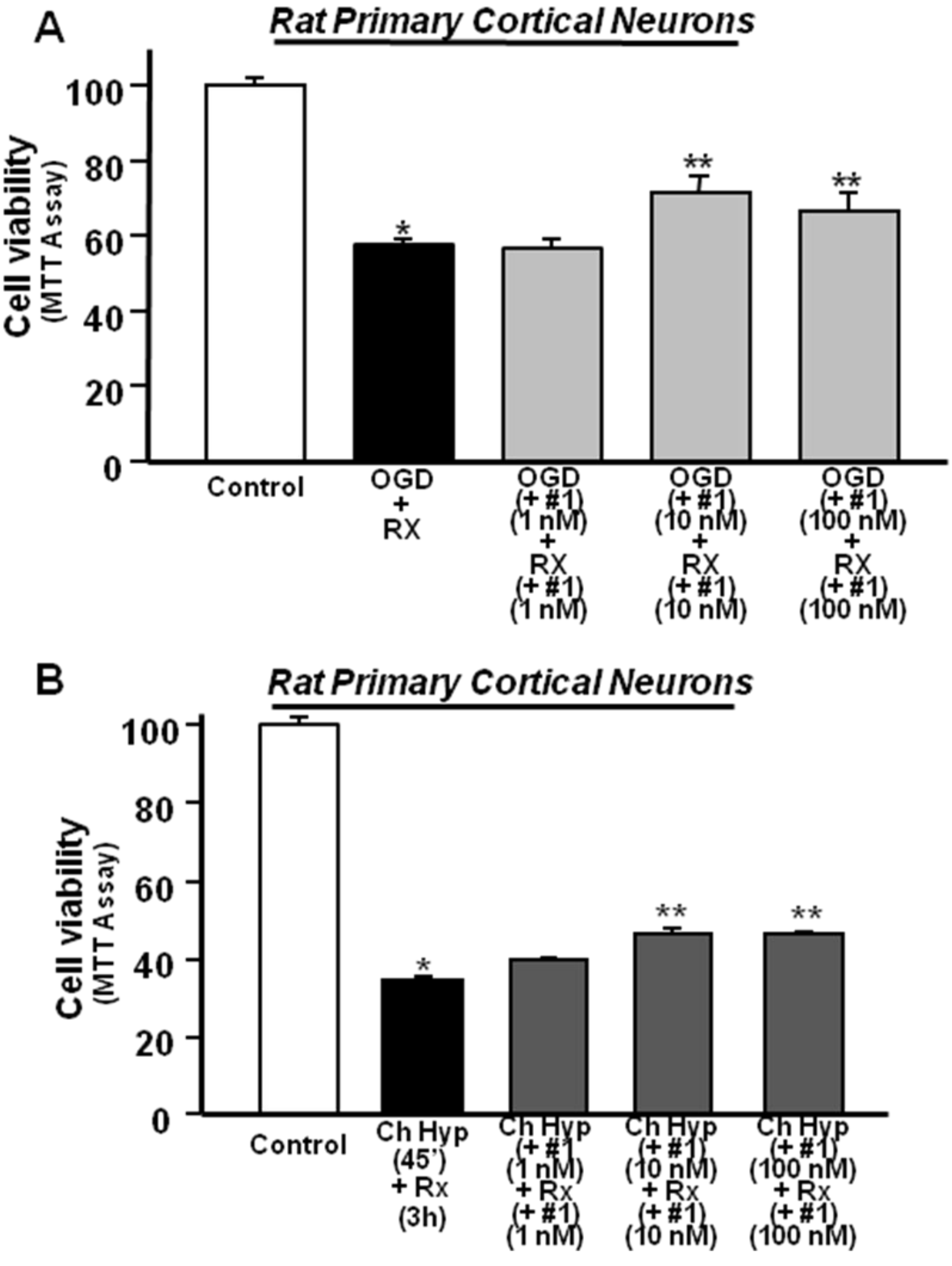

Figure S2. Concentration dependent neuroprotective profile in cortical neurons of compound **1**

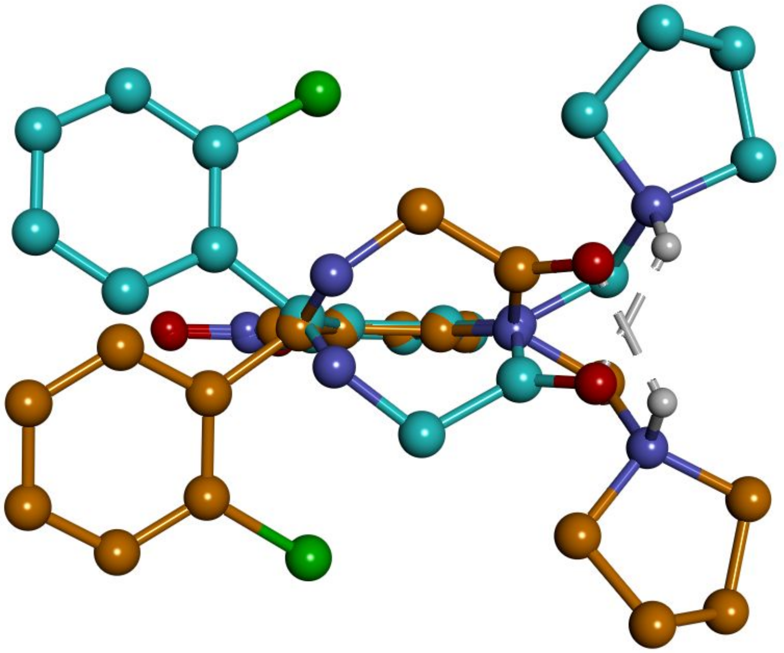

Figure S3. Comparison of the global minimum conformers (ΔE_{GM} = 0 kcal/mol) of **1** having the opposite flip of the benzodiazepinone ring**.** Structures were superimposed by the sp^2 heavy atoms of the benzodiazepinone ring and are displayed as ball and sticks. Hydrogens are omitted for clarity of presentation with the exception of those involved inhydrogen bonds. Hydrogen bonds are displayed as white dashed lines.

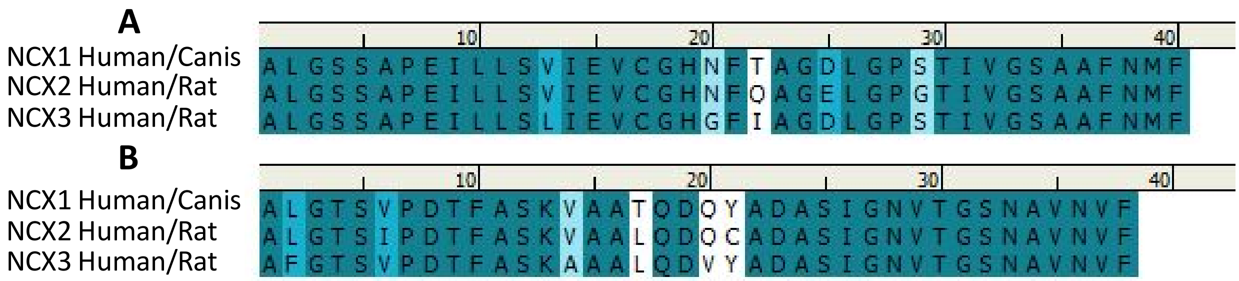

Figure S4. (A) The α1 repeat region identified to be involved in Neurounina-1 binding to NCX1. (B) The α2 repeat region identified to be involved in Neurounina-1 binding to NCX1.

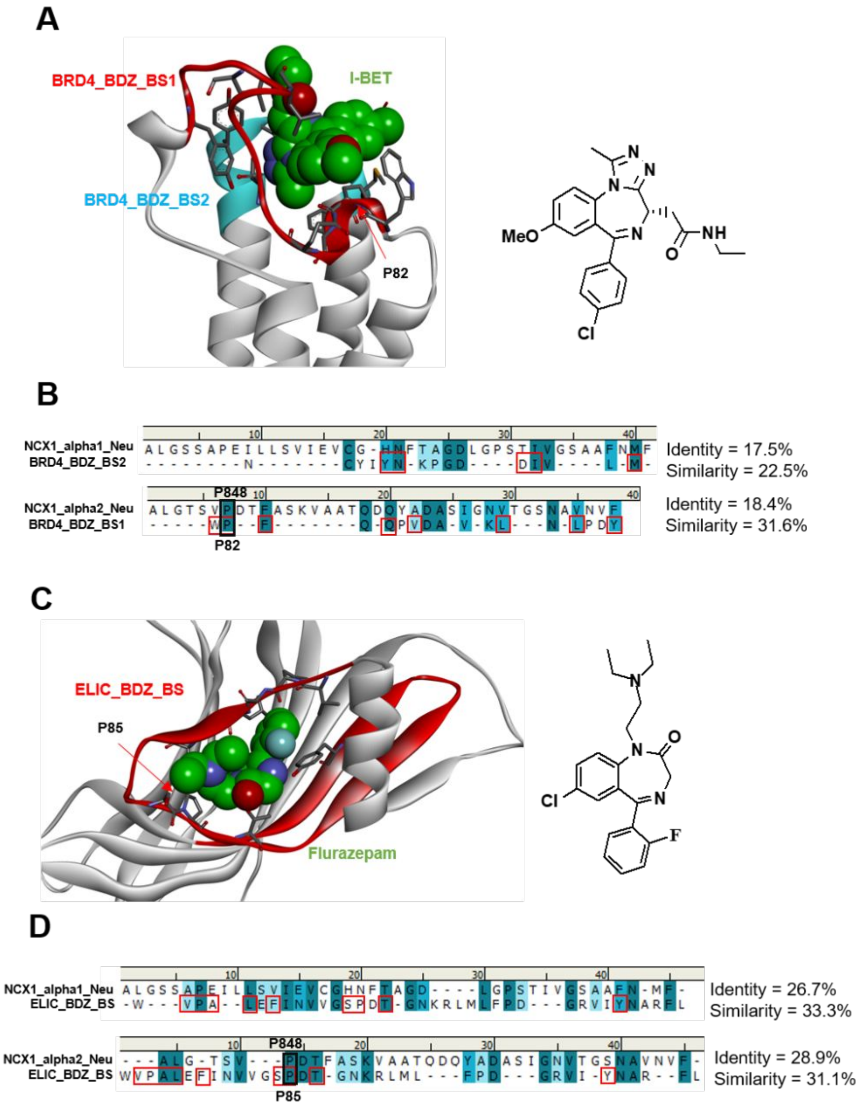

Figure S5. (A) X-ray structure of the BRD4 bromodomain in complex with I-BET (PDB ID : 3P5O). The binding site is colored in red (BRD4_BDZ_BS_1) and cyan (BRD4_BDZ_BS_2). The BRD4 residues establishing interactions with I-BET are displayed as sticks, the ligand is displayed as CPK. (B) Sequence alignments of the α1 and α2 repeat regions suggested to be involved in Neurounina binding with the BRD4 alprazolam binding site. BRD4 residues establishing interactions with the benzodiazepine ligand are evidenced with red squares. P848 (NCX1) and P85 (BRD4) are labelled.

(C) X-ray structure of the ELIC in complex with flurazepam (PDB ID : 2YOE). The binding site is colored in red (ELIC_BDZ_BS). The ELIC residues establishing interactions with flurazepam are displayed as sticks, the ligand is displayed as CPK. (D) Sequence alignments of the α1 and α2 repeat regions suggested to be involved in Neurounina binding with the ELIC flurazepam binding site. ELIC residues establishing interactions with the benzodiazepine ligand are evidenced with red squares. P848 (NCX1) and P85 (ELIC) are labelled.

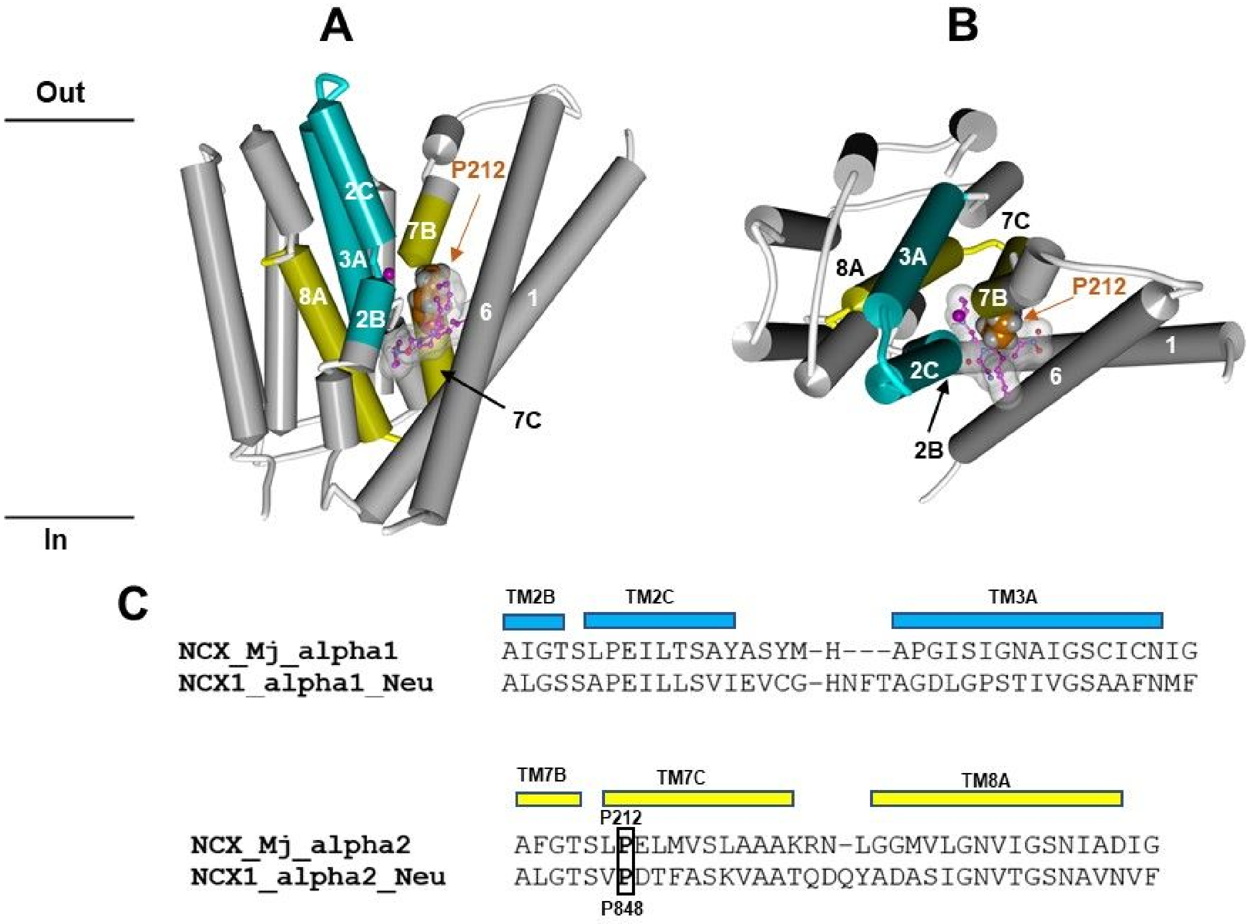

Figure S6. Longitudinal (A) and transverse (B) views of the molecular interaction model between compound **4** and NCX_mj resulting from our bioinformatics and structural analysis. The X-ray structure of NCX_mj transporter in the calcium loaded occluded conformation (PDB ID: 5HXR) is colored in grey with NCX1_alpha1_Neu and NCX1_alpha2_Neu evidenced in cyan and yellow, respectively. The protein structure is displayed as follows: helical structures as wide cylinders, beta-sheets as arrows, and coil and turn regions as tubes. The calcium atoms are displayed in ball&stick and colored in magenta. The putative bioactive conformer of **4** is displayed in ball&stick and colored by atoms (C = pink, O = red, and N = blue). Compound **4** solvent accessible surface is showed and colored in white/transparent. Proline P212 is evidenced in CPK and colored in orange. (C) Sequence alignments of the α1 and α2 repeat regions of human NCX1 suggested to be involved in neurounina-1 binding with the corresponding segments of NCX_Mj (ref. 31 in main text). NCX_Mj P212 and NCX1 P848 proline residues are evidenced and labeled.

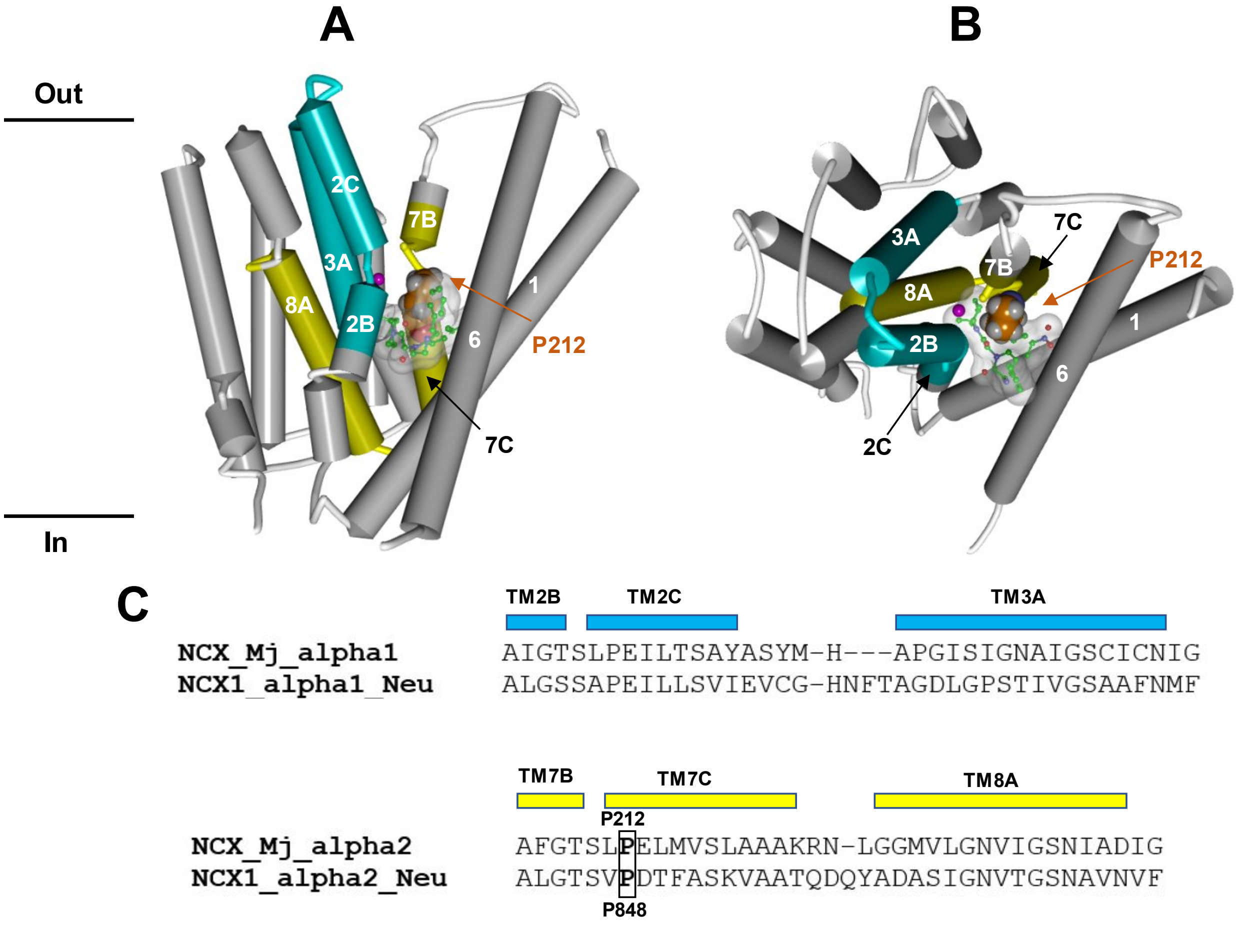

Figure S7. Longitudinal (A) and transverse (B) views of the molecular interaction model between the compound **14** and NCX_mj resulting from our bioinformatics and structural analysis. The X-ray structure of NCX_mj transporter in the calcium loaded semiopen conformation (PDB ID: 5JDF) is colored in grey with NCX1_alpha1_Neu and NCX1_alpha2_Neu evidenced in cyan and yellow, respectively. The protein structure is displayed as follows: helical structures as wide cylinders, beta-sheets as arrows, and coil and turn regions as tubes. The calcium atom is displayed in ball&stick and colored in magenta. The putative bioactive conformer of **14** is displayed in ball&stick and colored by atoms (C = green, O = red, and N = blue). Compound **14** solvent accessible surface is showed and colored in white/transparent. Proline P212 is evidenced in CPK and colored in orange. (C) Sequence alignments of the α1 and α2 repeat regions of human NCX1 suggested to be involved in neurounina-1 binding with the corresponding segments of NCX_Mj (ref. 31 in main text). NCX_Mj P212 and NCX1 P848 proline residues are evidenced and labeled.

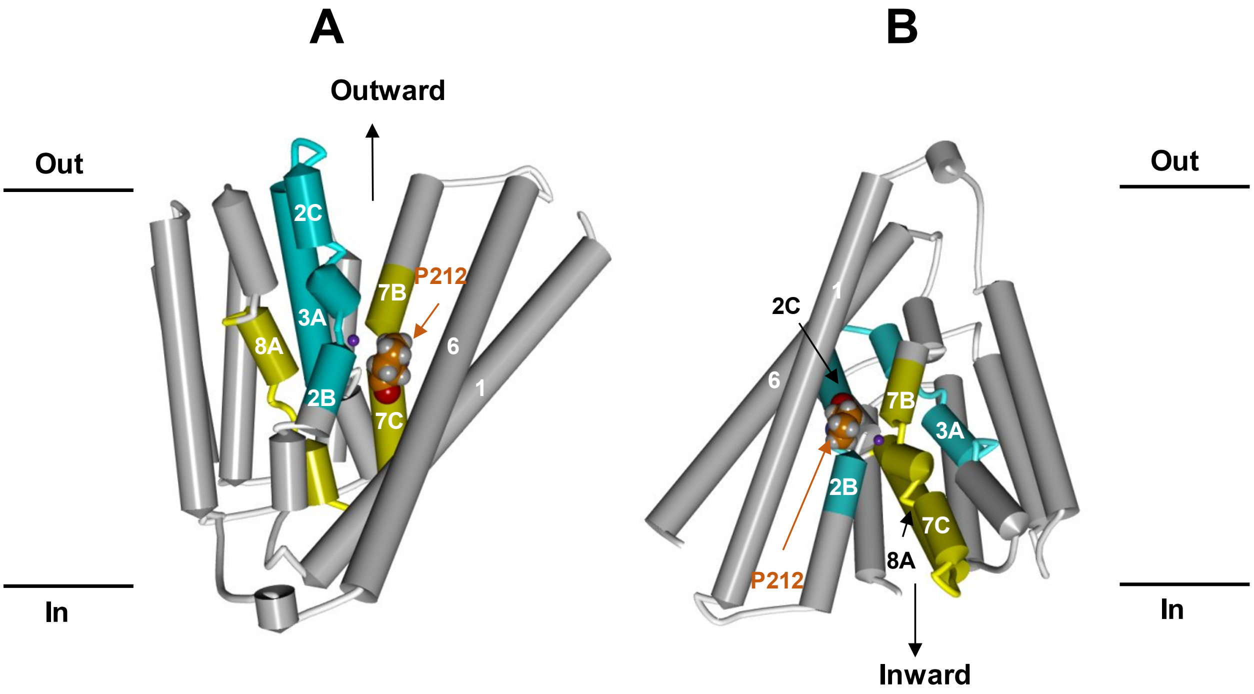

Figure S8. Topology model of the outward- (A) and inward-facing (B)NCX_mj. The X-ray structure of NCX_mj transporter in the sodium loaded semiopen conformation (PDB ID: 5HWY) is colored in white with NCX1_alpha1_Neu and NCX1_alpha2_Neu evidenced in cyan and yellow, respectively. The protein structure is displayed as follows: helical structures as wide cylinders, beta-sheets as arrows, and coil and turn regions as tubes. The sodium atoms are displayed in ball&stick and colored in violet. The proline P212 is evidenced in CPK and colored in orange.

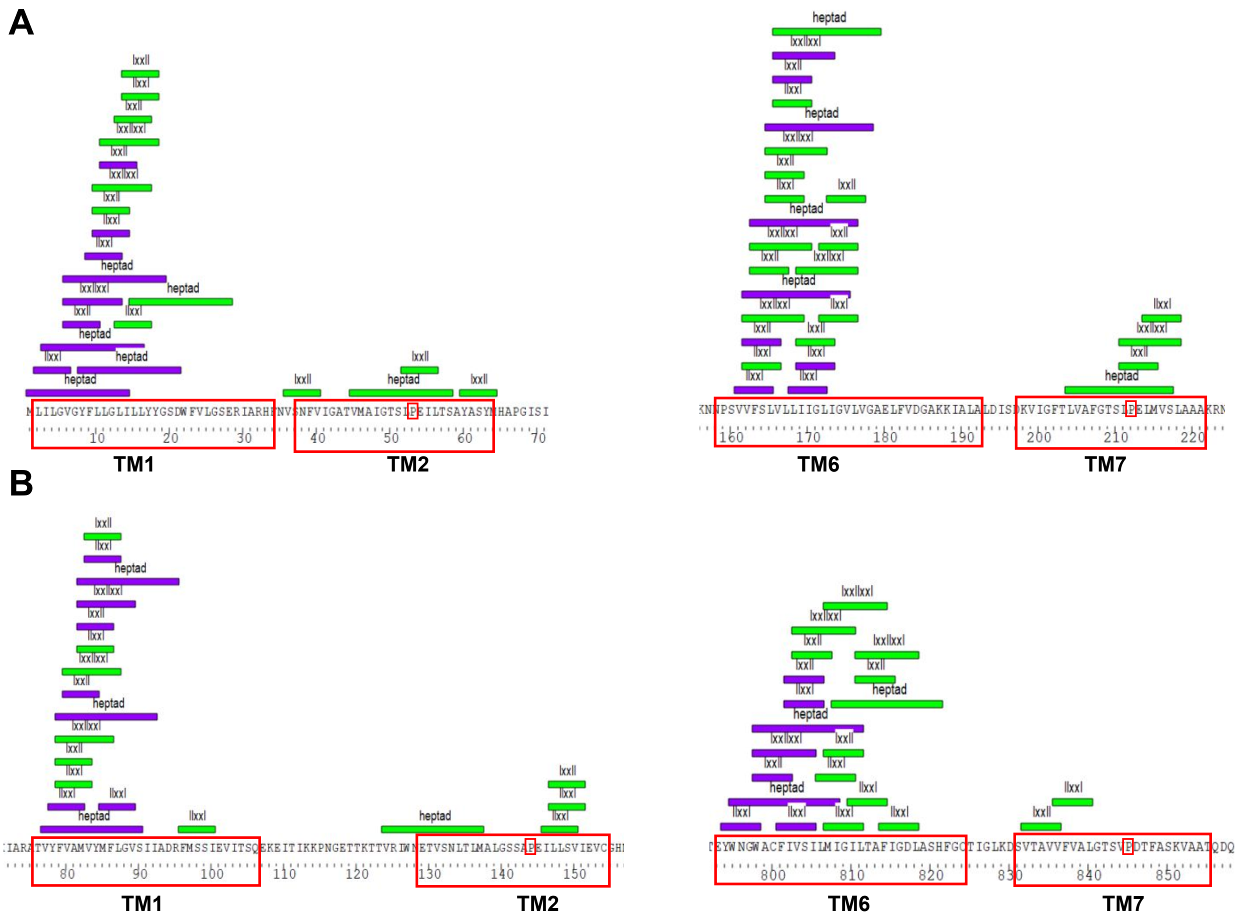

Figure S9. Predicted protein-protein interaction motifs (LxxLL-like and heptad repeats) on TM1, TM2, TM6 and TM7 of A) *Methanocaldococcus jannaschii* NCX and B) *Canis lupus familiaris* NCX1. The motifs conserved between the two species are coloured in violet while those not conserved are coloured in green. The proline residues on TM2C and TM7C are evidenced with a red square.

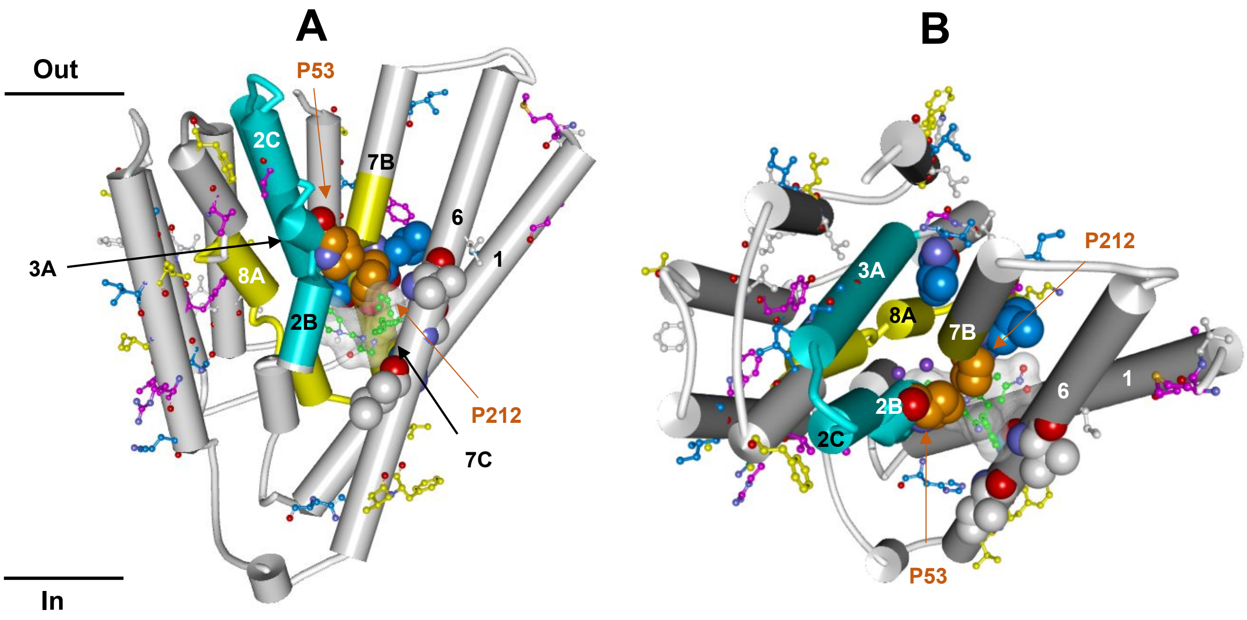

Figure S10. Longitudinal (A) and transverse (B) views of the molecular interaction model between **Neurounina-1** and NCX_Mj (PDB ID: 5HWY). Residues not conserved among *Canis lupus familiaris* NCX1, *Rattus* NCX2 and *Rattus* NCX3 are shown and colored as follows: white (residues not conserved among NCX1, NCX2 and NCX3); yellow (residues mutated only in NCX1); blue (residues mutated only in NCX2); magenta (residues mutated only in NCX3). Those within 5 Å from **Neurounina-1**, **4** and **14** are evidenced in CPK while the others are displayed in ball&stick. The protein structure is displayed as wide cylinders (helices) and tubes (coils and turns) and colored in white. NCX1_alpha1 and NCX1_alpha2 are evidenced in cyan and yellow, respectively. The putative bioactive conformer of **Neurounina-1** is displayed in ball&stick and colored by atoms (C = green, O = red, and N = blue). The solvent accessible surface of the ligand is colored in white/transparent. The conserved proline residues on TM2C and TM7C (P53 and P212; respectively) are coloured by atoms (C = orange) and displayed in CPK.

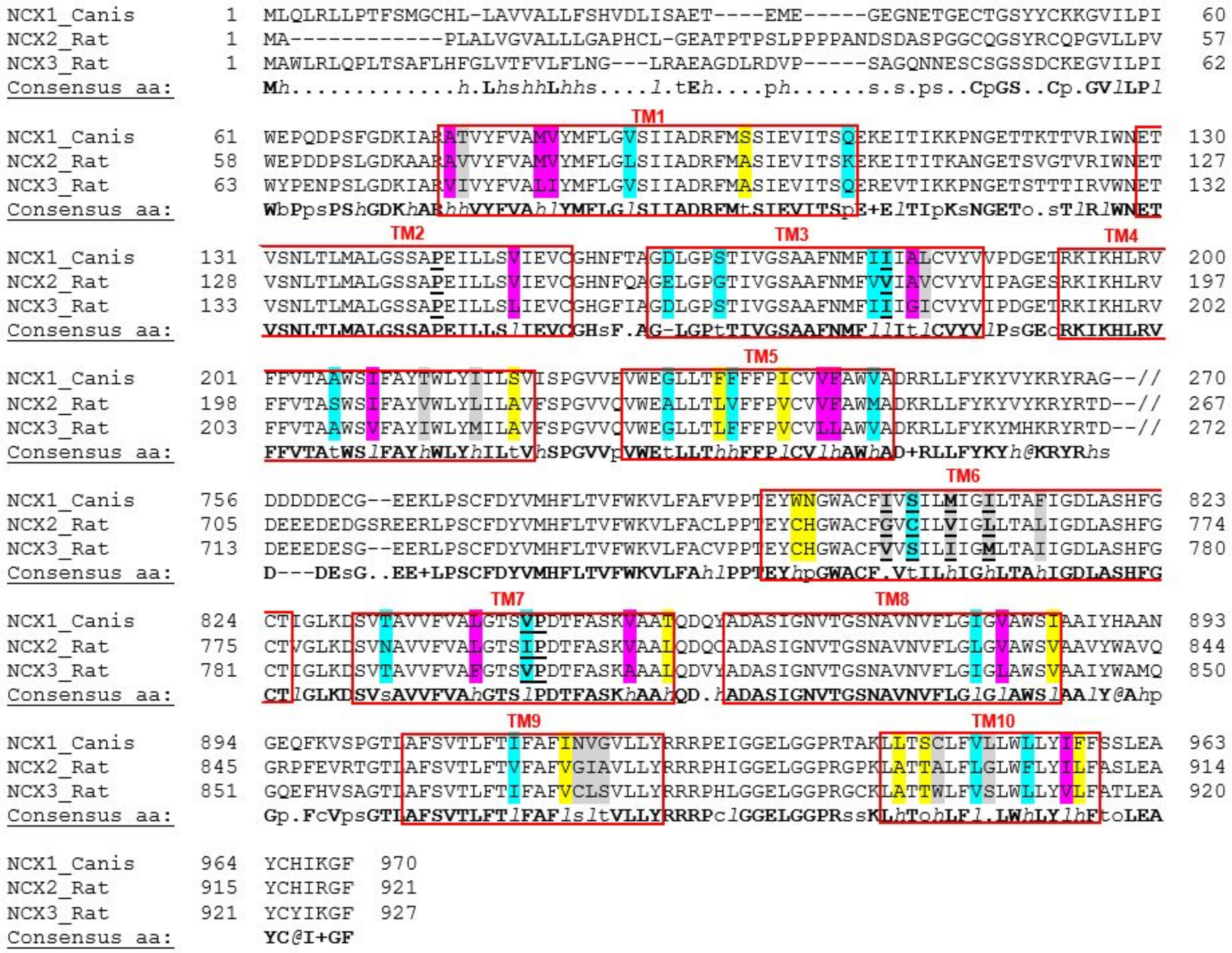

Figure S11. Multiple sequences alignment of *Canis lupus familiaris* NCX1, *Rattus* NCX2 and *Rattus* NCX3, excluding the f-loop. Residues not conserved among the three isoforms are highlighted in grey; residues mutated only in NCX1 are highlighted in yellow; residues mutated only in NCX2 are highlighted in cyan, residues mutated only in NCX3 are highlighted in magenta. The proline residues on TM2C and TM7C as well as the non-conserved residues within 5 Å from **Neurounina-1**, **4** and **14** are underlined and evidenced in bold.

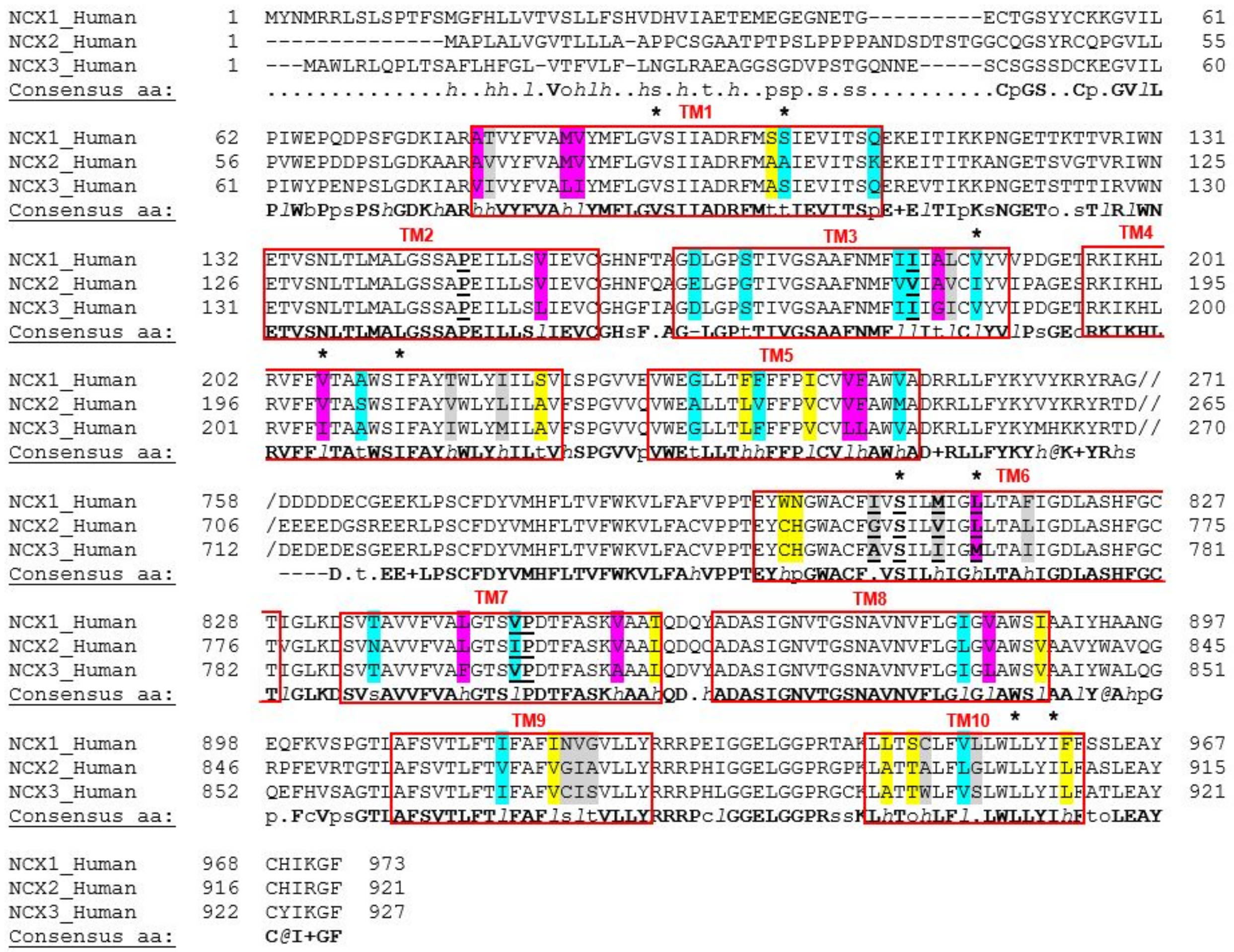

Figure S12. Multiple sequences alignment of *Human* NCX1-3, excluding the f-loop. Residues not conserved among the three isoforms are highlighted in grey; residues mutated only in NCX1 are highlighted in yellow; residues mutated only in NCX2 are highlighted in cyan, residues mutated only in NCX3 are highlighted in magenta; residues mutated with respect to *Canis lupus familiaris* (NCX1), *Rattus* (NCX2) and *Rattus* (NCX3) are labelled with an asterisk. The proline residues on TM2C and TM7C as well as the non-conserved residues within 5 Å from **Neurounina-1**, **4** and **14** are underlined and evidenced in bold.
